# Iso‐ and Benzisothiazolinone Inhibitors of Monoacylglycerol Lipase: Exploring On‐Target Activity Through Scaffold Decoration and In Situ Warhead Generation

**DOI:** 10.1002/cmdc.70414

**Published:** 2026-08-25

**Authors:** Edoardo Rocca, Annalida Bedini, Laura Scalvini, Francesca Galvani, Riccardo Castelli, Lorenzo Sarcone, Adriano Recchia, Fabiola Fanini, Adren Tran, Silvia Rivara, Gilberto Spadoni, Daniele Piomelli, Marco Mor

**Affiliations:** ^1^ Dipartimento di Scienze degli Alimenti e del Farmaco Università degli Studi di Parma Parma Italy; ^2^ Dipartimento di Scienze Biomolecolari Università degli Studi di Urbino “Carlo Bo” Urbino Italy; ^3^ Department of Anatomy and Neurobiology University of California Irvine California USA

**Keywords:** allosteric regulation, benzisothiazolinone, drug design, endocannabinoid system, medicinal chemistry, monoacylglycerol lipase

## Abstract

Monoacylglycerol lipase (MGL) is an intracellular serine hydrolase that regulates endocannabinoid signaling by hydrolyzing 2‐arachidonoylglycerol (2‐AG), thereby controlling cannabinoid receptor activity and related physiological processes. Targeting MGL through allosteric mechanisms is a promising strategy to modulate 2‐AG levels while avoiding limitations associated with active site–directed inhibitors. In this study, we aimed to develop selective MGL inhibitors based on isothiazolinone (ITZ) and benzisothiazolinone (BTZ) scaffolds that covalently target the regulatory cysteine residues Cys201 and Cys208 forming disulfide adducts. Two complementary approaches were explored. First, a mechanism‐based strategy was designed to exploit MGL‐mediated hydrolysis of *O*‐substituted (benz)isothiazol‐3‐ol derivatives, enabling localized release of the reactive warhead in proximity to the target cysteines. Second, to enhance molecular recognition, tailored substituents were introduced on the BTZ scaffold to promote selective interactions with residues surrounding the allosteric site. Although several compounds displayed nanomolar inhibitory activity, structure–activity relationship analysis indicated that inhibitory potency is primarily driven by intrinsic warhead reactivity rather than specific protein–ligand interactions. Overall, these findings highlight both the potential and the challenges of developing selective covalent allosteric MGL inhibitors, emphasizing the need for strategies that better balance reactivity and molecular recognition.

## Graphical abstract

We explore covalent allosteric targeting of MGL regulatory cysteines using iso‐ and benzisothiazolinone scaffolds. Combining mechanism‐based warhead release with structure‐guided scaffold decoration to improve molecular recognition, this study probes strategies to achieve selective on‐target engagement beyond the catalytic site.

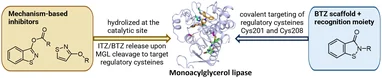

## 1 Introduction

Monoacylglycerol lipase (MGL) is a serine hydrolase that cleaves medium‐ and long‐chain monoacylglycerols into free fatty acids and glycerol [1, 2, 3]. It is widely expressed throughout the body, with particularly high levels in the central nervous system (CNS) [4, 5], where it is located at presynaptic nerve terminals [6]. MGL serves as the main intracellular enzyme responsible for the degradation of 2‐arachidonoyl‐*sn*‐glycerol (2‐AG), the most abundant endocannabinoid in the mammalian brain [7, 8]. Through this function, MGL is involved in several physiological and pathological processes, including analgesia [9, 10, 11, 12, 13], neuroprotection [14, 15, 16, 17], stress, and anxiety [18, 19]. 2‐AG is an endogenous agonist of CB1 and CB2 cannabinoid receptors [7, 8]. In neurons, it acts in a retrograde manner, being released from postsynaptic neurons to modulate presynaptic activity via CB1 receptors located on axon terminals [6, 20, 21]. Activating CB2 receptors, which are predominantly expressed in immune cells, 2‐AG also exerts immunomodulatory and antiinflammatory effects by suppressing proinflammatory cytokine release and regulating immune cell activation. Outside the CNS, MGL is broadly expressed and influences several peripheral functions, including immune system activity [22, 23], regulation of energy homeostasis and lipid metabolism [24], and cancer cell proliferation [12, 25], largely through its control of 2‐AG levels and arachidonic acid–derived eicosanoid signaling.

**FIGURE 1:**
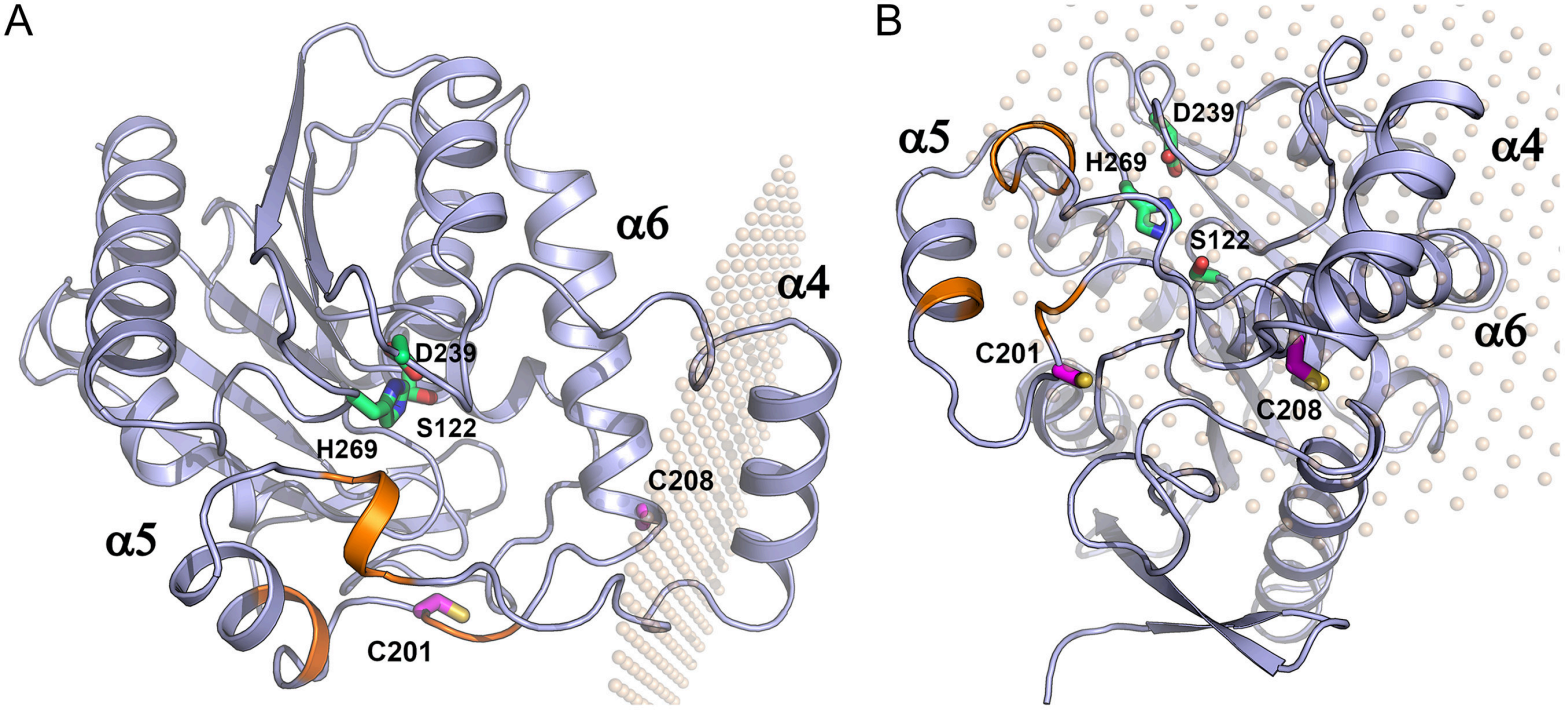
Side view (panel A) and top‐down view (panel B) of the X‐ray structure of human MGL with the lid domain in its open conformation (PDB ID: 3HJU, light‐blue cartoon). The positions of the phosphate groups of the membrane bilayer are shown as beige spheres (OPM database [28]). The catalytic triad residues Ser122, His269, and Asp239, together with the regulatory cysteines Cys201 and Cys208, are depicted as sticks with green and pink carbon atoms, respectively. Residues lining the proposed glycerol exit route are highlighted in orange.

MGL is a peripheral membrane‐associated serine hydrolase [26] characterized by a catalytic triad consisting of Ser122, His269, and Asp239 (numbering referred to human MGL) [27], placed at the extremity of a lipophilic substrate recognition cavity, enclosed by the three helices α4–6 and the disordered loops therein (Figure 1). This cavity ends with a “lid domain,” a regulatory U‐shaped region mainly composed of the α4 helix and the 4–5 loop. While this portion, and the α4 helix in particular, serves as anchoring point to the membrane, from which the substrate is recruited, the lid domain works as a flexible portal regulating the access to the catalytic site, being able to adopt open or closed conformations [29]. On the opposite side of the binding site and beyond the catalytic triad, hydrophilic residues shape a polar channel for glycerol allowing its release after substrate hydrolysis. The catalytic activity of MGL is tightly regulated by the redox state of Cys201 and Cys208, two residues located outside the catalytic site near the lid domain that act as key allosteric modulators [30]. In particular, hydrogen peroxide–mediated sulfenylation of these cysteines leads to redox‐dependent inhibition of MGL catalytic activity, serving as a physiological switch able to modulate MGL activity and, in turn, cellular 2‐AG levels in response to the redox state of the cell [30].

**FIGURE 2:**
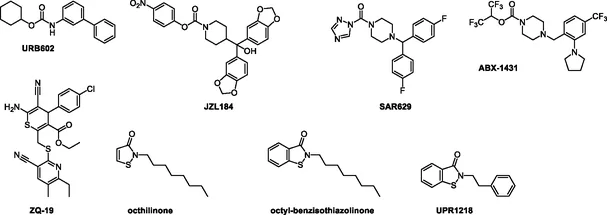
Structures of representative MGL inhibitors.

The pivotal role of MGL in regulating cannabinoid‐dependent processes makes it a promising therapeutic target in various disease conditions. Numerous MGL inhibitors have been developed, displaying diverse mechanisms of action [31, 32]. Starting from URB602, the first inhibitor reported to occupy the active site and inhibit MGL through a partially reversible mechanism [33], additional active site–directed inhibitors have been identified (Figure 2). These include covalent inhibitors, such as JZL184 [34], SAR629 [35], and ABX‐1431 [36], which carbamoylate the catalytic serine, as well as reversible inhibitors based on hexahydro‐pyrido‐oxazinone [37] or ortho‐hydroxyanilide scaffolds [38]. Recently, ZQ‐19, characterized by a nitrile warhead, has also been described [39]. An efficient alternative strategy for MGL inhibition is based on the covalent modification of regulatory cysteine residues Cys201 and Cys208. Isothiazolinone (ITZ) and benzisothiazolinone (BTZ) derivatives (Figure 2) have been identified as potent MGL allosteric inhibitors capable of forming disulfide adducts with regulatory cysteines [40]; these adducts are predicted to favor a closed conformation of the lid domain, hampering substrate recruitment [29]. Unlike active site–directed inhibitors, ITZs and BTZs act allosterically and therefore do not compete with 2‐AG, whose concentrations in the CNS can reach high micromolar levels; at the same time, by avoiding direct engagement of the catalytic serine, they may circumvent the excessive 2‐AG accumulation associated with irreversible MGL inhibitors and the consequent desensitization of CB1 receptors. The BTZ UPR1218 displayed nanomolar inhibitory activity, demonstrated in vitro neuroprotective activity, and elevated brain 2‐AG levels in mice [41]. These findings highlight the relevance of developing small‐molecule allosteric MGL inhibitors that mimic the ability of the intracellular second messenger hydrogen peroxide to modulate MGL activity and enhance 2‐AG‐mediated signaling. Although UPR1218 does not significantly affect the activity of other cysteine‐rich proteins (TRPV1 and TRPA1) or several key components of the endocannabinoid system (DGL‐α, ABHD6, and FAAH), the BTZ class is highly reactive toward cysteines and has been shown to covalently modify multiple enzymes [42, 43, 44, 45, 46].

In the present study, we sought to achieve targeted activity of ITZ and BTZ derivatives toward the regulatory cysteine residues of MGL through two complementary approaches. The first is a mechanism‐based strategy that exploits MGL‐mediated processing to release the active warhead in close proximity to the target cysteines. The second approach focuses on enhancing molecular recognition by appropriately decorating the BTZ scaffold to promote specific interactions with residues on the MGL surface, thereby minimizing nonspecific reactivity and limiting off‐target engagement.

## 2 Results and Discussion

### 2.1 Chemistry

**SCHEME 1:**
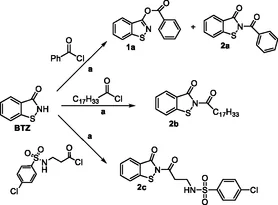
Synthesis of *O*‐acyl and *N*‐acyl benzisothiazolinone derivatives. Reagents and conditions: (a) ZnCl_2_, TEA, acetone, 0 °C, 30 min (10 min for **2b**).

The synthesis of *O*‐ or *N*‐acyl‐1,2‐benzisothiazoles **1a** and **2a–c** (Scheme 1) was carried out using reaction conditions previously described for *O*‐acylation of commercially available benzisothiazolinone (BTZ) [47]. In our hands, the benzoylation of this substrate yielded an 8:2 mixture of *O*‐ (**1a**) and *N*‐ (**2a**) isomers that were separated by silica gel column chromatography.

Acylation of BTZ with oleoyl chloride (for **2b**) or with 3‐[(4‐chlorophenyl)sulfonamido]propanoyl chloride [48] (for **2c**) afforded a mixture of *O*‐acyl and *N*‐acyl derivatives in an approximate ratio of 1:2, as determined by ^1^H NMR analysis of the crude reaction mixture. However, it was not possible to isolate the *O*‐acylated products due to their very rapid rearrangement to the corresponding *N*‐acylated compounds **2b**,**c** (Scheme 1).

**SCHEME 2:**
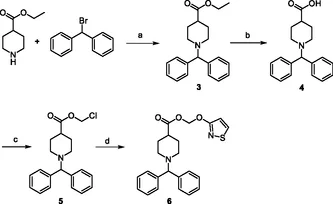
Synthesis of compound **6**. Reagents and conditions: (a) K_2_CO_3_, DMF, 80 °C, 18 h; (b) NaOH, MeOH/H_2_O, 60 °C, 18 h; (c) chloromethyl chlorosulfate, NaHCO_3_, (*n‐*Bu)_4_NHSO_4_, DCM/H_2_O, r.t., 18 h; and (d) isothiazolinone, K_2_CO_3_, (*n‐*Bu)_4_NHSO_4_, DCM/H_2_O, r.t., 20 h.

The target compound **6** (Scheme 2) was obtained by treatment of isothiazolinone with the chloromethyl ester **5**, in the presence of K_2_CO_3_ and tetrabutylammonium bisulfate. In turn, the intermediate **5** was prepared by alkylation of commercially available ethyl 4‐piperidinecarboxylate with benzhydryl bromide in the presence of K_2_CO_3_, followed by base‐promoted hydrolysis of ester **3** and treatment of the corresponding acid **4** with chloromethyl chlorosulfate, tetrabutylammonium bisulfate, and sodium bicarbonate.

**SCHEME 3:**
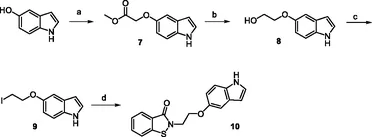
Synthesis of compound **10**. Reagents and conditions: (a) methyl chloroacetate, NaH 60%, DMF, −10 °C to r.t., 16 h; (b) LiAlH_4_, THF, 0 °C, 1.5 h; (c) I_2_, imidazole, (Ph)_3_P, DCM, 0 °C to r.t., 24 h; and (d) benzisothiazolinone, K_2_CO_3_, CH_3_CN, reflux, 24 h.

The synthetic procedure used to prepare the *N*‐alkylated benzisothiazolinone derivative **10** is illustrated in Scheme 3. Briefly, *O*‐alkylation of commercially available 5‐hydroxyindole with methyl chloroacetate in the presence of NaH and subsequent reduction of the intermediate ester **7** with LiAlH_4_ provided the alcohol **8** that was converted to the corresponding iodide (**9**) by treatment with iodine in the presence of triphenylphosphine and imidazole. Alkylation of BTZ with 5‐(iodoethoxy)indole (**9**) in the presence of K_2_CO_3_ gave a mixture of *N*‐alkylated (**10**) and *O*‐alkylated products that were separated by silica gel column chromatography.

**SCHEME 4:**
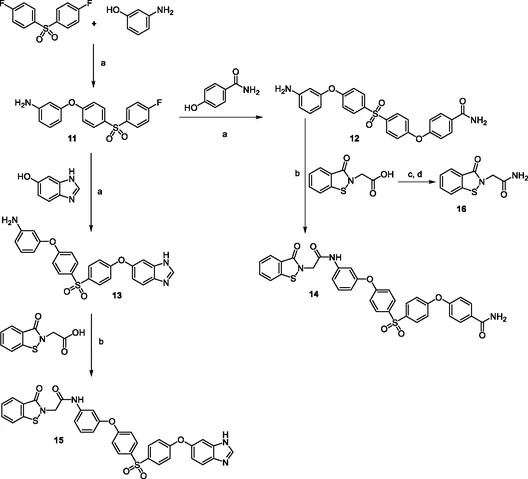
Synthesis of compounds **14**, **15**, and **16**. Reagents and conditions: (a) Cs_2_CO_3_, DMF, 60 °C, 2 h; (b) EDCI, HOBT, DMF, 60 °C, 16 h for **14** and r.t, 6 h for **15**; (c) oxalyl chloride, DMF, DCM, 0 °C to r.t., 2 h; and (d) conc. NH_4_OH, DCM, r.t., 15 min.

The target compounds **14** **–16** were synthesized as depicted in Scheme 4. Briefly, nucleophilic aromatic substitution of the commercially available 4,4′‐difluorodiphenyl sulfone with 3‐aminophenol in the presence of cesium carbonate afforded the intermediate **11**, which was submitted to a further nucleophilic aromatic substitution by treatment with 4‐hydroxybenzamide or 6‐hydroxy‐1*H*‐benzimidazole to give the aniline derivatives **12** and **13**, respectively. The desired final compounds **14** and **15** were obtained via amide coupling reaction of the commercially available 2‐(3‐oxobenzo[*d*]isothiazol‐2(3*H*)‐yl)acetic acid with anilines **12** and **13**, respectively, in the presence of EDCI/HOBT as coupling agent. An analogous synthetic strategy was used to prepare the target compound **16** by reacting 2‐(3‐oxobenzo[*d*]isothiazol‐2(3*H*)‐yl)acetic acid with oxalyl chloride in the presence of a catalytic amount of DMF, followed by treatment of the intermediate acyl chloride with an aqueous solution of ammonia (Scheme 4).

**SCHEME 5:**
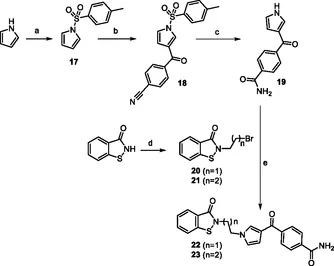
Synthesis of compounds **22** and **23**. Reagents and conditions: (a) *p*‐toluenesulfonyl chloride, NaH 60%, THF, 0 °C, 1 h; (b) 4‐cyanobenzoyl chloride, AlCl_3_, DCM, r.t., 6 h; (c) H_2_O_2_, NaOH, DMSO/MeOH 9:1, 0 °C, 0.5 h; (d) 1,2‐dibromoethane or 1,3‐dibromopropane, K_2_CO_3_, DMF, 60 °C, 1 h; and (e) NaH, DMF, r.t., 1 h.

The BTZ derivatives **22** and **23** were synthesized following the strategy depicted in Scheme 5. Pyrrole was at first protected with a *p*‐toluenesulfonyl group [49]. Friedel–Crafts acylation of **17** with 4‐cyanobenzoyl chloride, mediated by aluminum trichloride, furnished intermediate **18** [50], whose subsequent *N*‐deprotection and conversion of the nitrile group to the corresponding carboxyamide in a single step yielded intermediate **19**. Derivatives **20** and **21** were obtained by reacting benzisothiazolinone under basic conditions (potassium carbonate in acetonitrile) with 1,2‐dibromoethane or 1,3‐dibromopropane [51] and then employed to functionalize the nitrogen atom of the pyrrole ring of **19**, affording compounds **22** and **23**, respectively.

### 2.2 Structure–Activity Relationships

#### 2.2.1 Mechanism‐Based MGL Inhibitors

**SCHEME 6:**
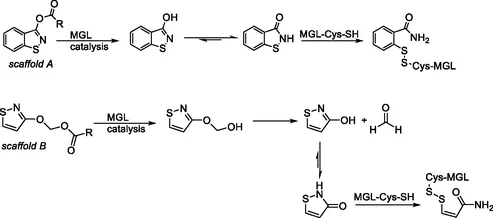
Proposed mechanism‐based activation of *O*‐substituted (benz)isothiazol‐3‐ol derivatives.

As a first approach, we investigated isothiazolinone‐ and benzisothiazolinone‐based compounds designed to undergo MGL‐mediated activation, thereby releasing the active inhibitor in close proximity to the regulatory residues Cys201 and Cys208. We hypothesized that either a 3‐acyloxy‐1,2‐benzisothiazole (scaffold A) or a 3‐acyloxymethyloxy‐isothiazole (scaffold B) could serve this purpose (Scheme 6). These *O*‐substituted (benz)isothiazol‐3‐ol derivatives are characterized by an aromatic benzisothiazole or isothiazole ring unable to react with cysteines. Upon MGL‐mediated cleavage of the ester group, tautomerism (for scaffold A) or spontaneous release of formaldehyde followed by tautomerism (for scaffold B) would generate cysteine‐reactive BTZ or ITZ just close to the target cysteines, thus minimizing the risk to react with off‐targets (Scheme 6).

To investigate whether benzisothiazole‐ and isothiazole‐based compounds could theoretically be activated by MGL, we performed molecular docking studies in the catalytic site of the enzyme in its open conformation.

To validate the noncovalent docking protocol, the cocrystallized 2*H*‐benzo[*b*][1,4]oxazin‐3(4*H*)‐one inhibitor (pdb: 7L4T) was redocked into the MGL substrate‐binding site. The predicted binding mode closely matched the crystallographic pose, with an RMSD of 1.10 Å calculated over the ligand heavy atoms (Figure S1A). We evaluated a simple benzoyl derivative, as a proof‐of‐concept compound (**1a**) and a benzhydrylpiperidine derivative (**6**) with a Y‐shaped scaffold characteristic of selective MGL inhibitors like SAR629 and JZL184 in Figure 2. The piperazine ring of these carbamoylating agents was replaced with a piperidine to prevent covalent inactivation of the enzyme, while the oxymethylene linker was introduced to avoid spontaneous hydrolysis of aryl esters, which was a risk for **1a** and, in general, for scaffold A.

**FIGURE 3:**
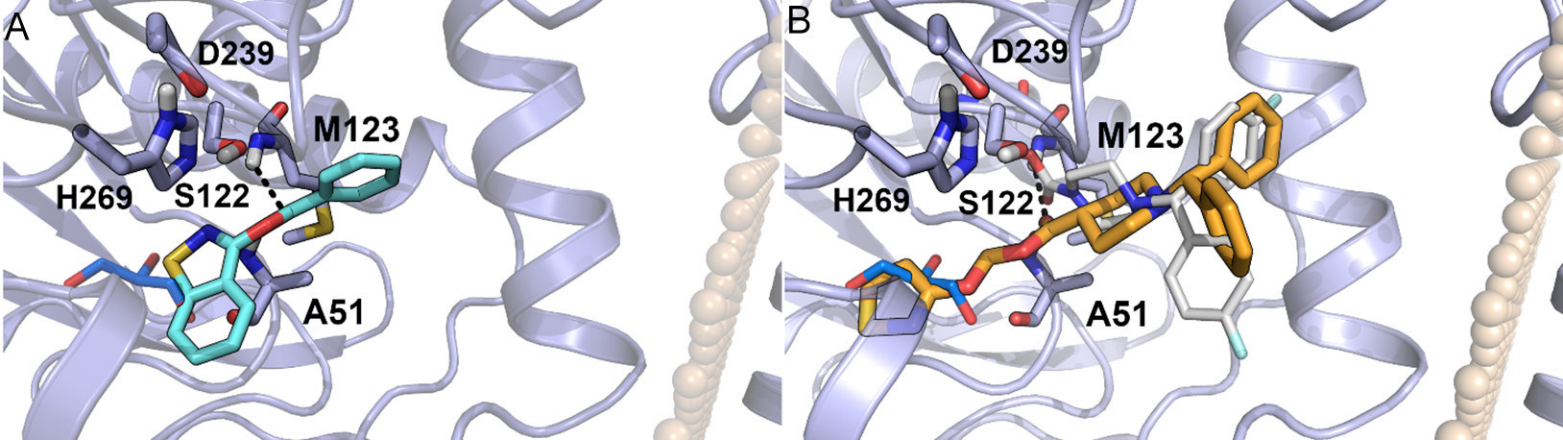
Docking poses of compound **1a** (cyan carbon atoms, panel A) and of **6** (orange carbon atoms, panel B) within the catalytic site of MGL (light‐blue cartoon). Hydrogen bonds formed with the oxyanion residues are shown as black dashed lines. The docking poses of **1a** and **6** are overlaid with either the crystallographic coordinates of a glycerol molecule (blue carbon atoms; PDB ID: 3HJU) or with those of the inhibitor SAR629 (gray carbon atoms) covalently bound to the catalytic Ser122 (PDB ID: 3JWE). Beige spheres represent the positions of the phosphate groups of the membrane bilayer.

Analysis of the docking poses showed that the benzo[*d*]isothiazol‐3‐yl benzoate **1a** suitably fits the enzyme binding site. The ester group to be hydrolyzed was positioned in proximity of the catalytic triad, with the carbonyl oxygen taking hydrogen bonds with the oxyanion hole residues, Met123 and Ala51, and the carbonyl carbon located at an approximate distance of 3 Å from the catalytic Ser122, consistent with a geometry prompting a nucleophilic attack. The benzisothiazole fragment is positioned in the slightly hydrophilic pocket that accommodates the leaving groups of covalent inhibitors and that in a crystal structure is occupied by a glycerol molecule (PDB id: 3HJU, Figure 3A) [52, 53]. This pocket is continuous with the glycerol “exit hole” which connects the enzyme active site and the cytosolic environment in close proximity to Cys201 and near Cys208 [34]. The phenyl ring of the compound is accommodated in the distal end of the hydrophobic substrate entry channel, the region that likely accommodates the arachidonoyl chain of 2‐AG.

The benzhydrylpiperidine derivative **6** was predicted to adopt a similar binding mode for the ester portion. The oxymethylene spacer (═OCH_2_═), derived from formaldehyde, allowed good accommodation of the smaller isothiazole fragment within the glycerol‐binding pocket. In this way, the Y‐shaped benzhydrylpiperidine fragment occupied the acyl chain‐binding channel adopting an arrangement consistent with that assumed by the irreversible inhibitor SAR629 covalently bound to the catalytic Ser122 (PDB id: 3JWE, Figure 3B).

**FIGURE 4:**
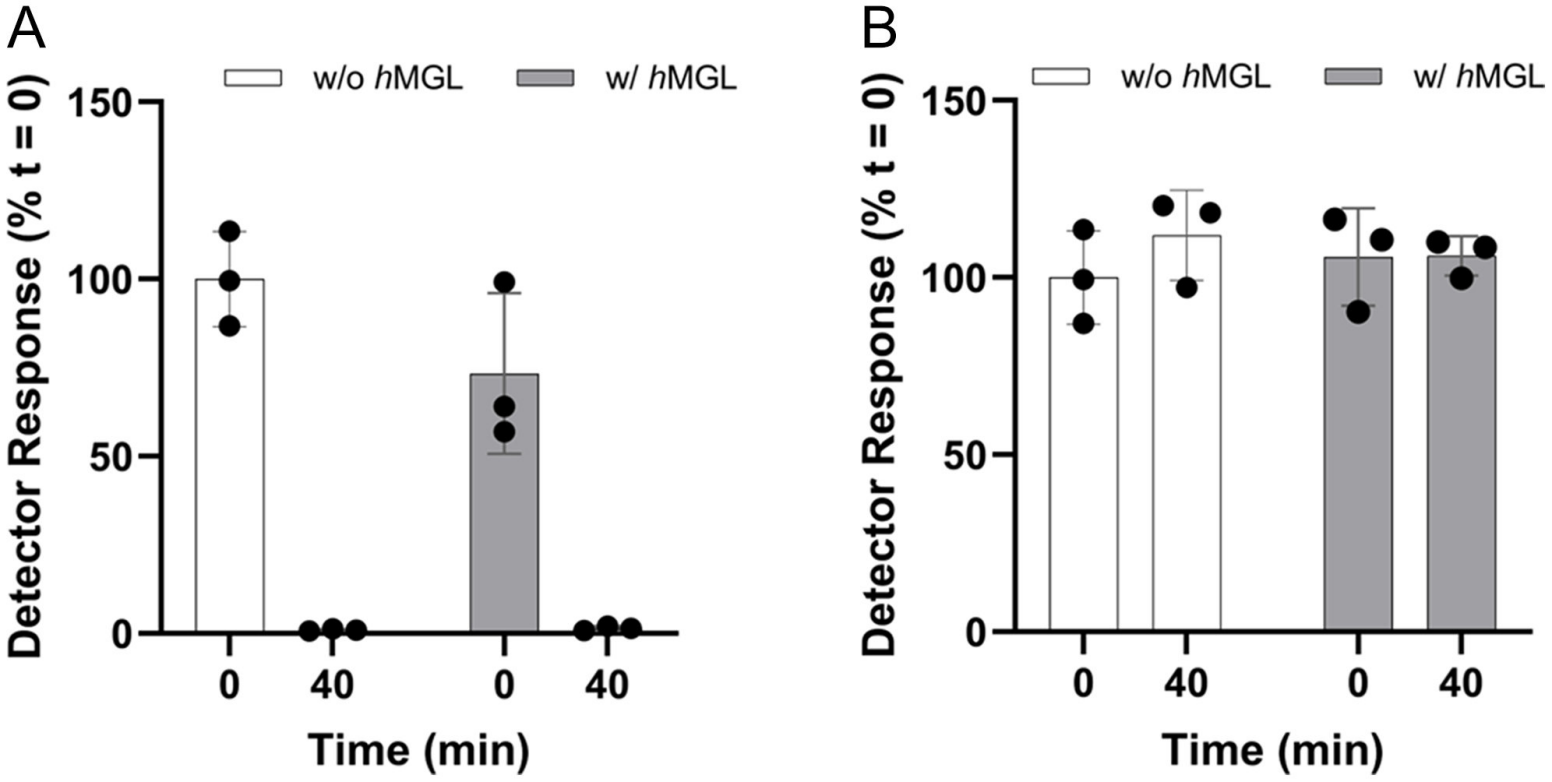
Stability of compounds **1a** (panel A) and **6** (panel B) at 100 nM concentration in assay buffer (enzyme‐free, white bars) and in the presence of hMGL (gray bars), evaluated by LC–MS/MS detector response at *t* = 0 and *t* = 40 min. Data are normalized to the detector response obtained at *t* = 0 in the assay buffer.

Before evaluating the ability of **1a** and **6** to inhibit MGL, we assessed their stability. To this end, the compounds were dissolved in the MGL assay buffer, in the absence of the enzyme. Whereas **1a** proved to be highly unstable, with complete disappearance after 40 min of incubation, **6** showed complete stability (Figure 4). The same experiment was then performed in the presence of MGL. After 40 min, no decrease in the concentration of **6** was observed, indicating that the compound is not hydrolyzed by MGL.

Therefore, the high IC_50_ value observed for **1a** (1672 nM, *n* = 2) can reasonably be attributed to the formation, under the assay conditions, of BTZ capable of reacting with cysteine residues (Table 1). In contrast, the chemical and enzymatic stability of **6** resulted in an IC_50_ > 10 μM. The compound was tested up to 10 μM concentration, giving an average inhibition of MGL activity, at 10 μM, of ≈10% (*n* = 2, Figure S2). The inability of MGL to hydrolyze **6** may be attributed to the local environment of the ester moiety, which could hinder nucleophilic attack by the catalytic serine. Indeed, whereas the carbonyl carbon of the substrate ester is linked to an alkyl chain, in **6**, it is bonded to a tertiary carbon, potentially generating steric hindrance. This hypothesis is supported by a QM/MM investigation of the carbamoylation mechanism of the catalytic serine by a covalent 4‐benzhydrylpiperazinyl triazolyl urea inhibitor. Indeed, this reaction, which corresponds to the acylation step of nucleophilic serine by our putative substrate, had been shown [54] to depend on rotation of the piperazine ring to achieve productive covalent bond formation. Such a conformational rearrangement may be hindered by the tertiary carbon of the piperidine scaffold in compound **6**. Accordingly, reducing the steric constraints around this region, for example, by introducing a flexible methylene spacer, might facilitate the conformational adaptation required for nucleophilic attack and thereby enable enzymatic hydrolysis.

**TABLE 1:** Inhibition of human MGL by iso‐ and benzisothiazolinone derivatives.^a^

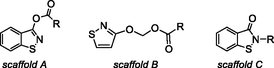			
Compound	Scaffold	R	IC_50_, nM
**1a**	A	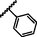	1672 (*n* = 2)^b^, ^c^
**2a**	C	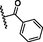	45.7 ± 13.6
**2b**	C	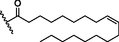	57.8 ± 11.1
**2c**	C	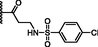	55.7 ± 22.8
**6**	B	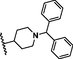	>10,000 (*n* = 2)^c^
**10**	C	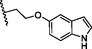	178.9 ± 58.6
**14**	C	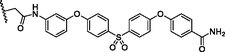	136.9 ± 43.4
**15**	C	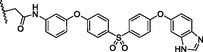	91.0 ± 24.4
**16**	C	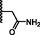	7.8 ± 1.6
**22**	C	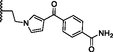	163.3 ± 71.7
**23**	C	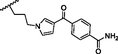	232.5 ± 60.3
**UPR1218**	C	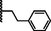	92.7 ± 32.7

a IC_50_ values were calculated from dose−response inhibition curves of 2‐oleoylglycerol (2‐OG) hydrolysis on *h*MGL and a 10‐min preincubation with the test compound. Results are expressed as mean ± SEM (*n* = 3) except for **1a** (*n* = 2).

b The two independent IC_50_ determinations were 1569 and 1775 nM.

c Given the low potency observed, only two experiments were performed.

Moreover, it cannot be excluded that the isothiazole exhibits suboptimal complementarity with the glycerol‐binding site. In any case, given the inability of these *O*‐substituted (benz)isothiazol‐3‐ol derivatives to inhibit MGL, we decided to halt the synthesis of further compounds and focused on the design of *N*‐substituted benzisothiazolinones with the aim to improve recognition by MGL and thus affinity and selectivity.

#### 2.2.2 *N*‐Substituted Benzisothiazolinones

Numerous BTZs have been described as potent MGL inhibitors, displaying IC_50_ values in the nanomolar range [40, 41]. However, these compounds typically bear small and structurally simple substituents on the nitrogen atom, not intended to confer selectivity for MGL and may allow interactions with other macromolecules containing accessible cysteines. **UPR1218**, which carries a phenethyl substituent, exhibits an IC_50_ of 93 nM (Table 1). To promote selective interaction with MGL, a series of BTZs substituted at the nitrogen atom with tailored groups was designed, with the aim of establishing specific interactions with amino acid residues located in the vicinity of Cys201 or Cys208. A challenge of this approach lies in the relatively flat surface of MGL in the region of interest, which lacks any well‐defined solvent‐exposed pocket. For the design of the new compounds, we adopted a covalent docking strategy to evaluate the interactions established with functional groups on the MGL surface following disulfide bond formation with Cys201 or Cys208. To validate the covalent docking protocol, we reproduced the arrangement of BTZ derivatives in two different settings observed in crystallized structures: In the first case (pdb: 8A1K) [55], the ligand is accommodated inside a well‐defined, structured binding site; in the second one (pdb: 9REY) [56], the covalently modified cysteine is more solvent‐exposed. The best poses were essentially superimposable to the crystallized ones. For docking into the defined region, we obtained an RMSD = 0.33 Å calculated over ligand heavy atoms, while in the case of solvent‐exposed cysteine, the RMSD was 1.10 Å (Figure S1B,C). In both cases, the orientation of the cysteine sulfur atom and of the thiophenol moiety generated upon BTZ ring opening was accurately reproduced.

**FIGURE 5:**
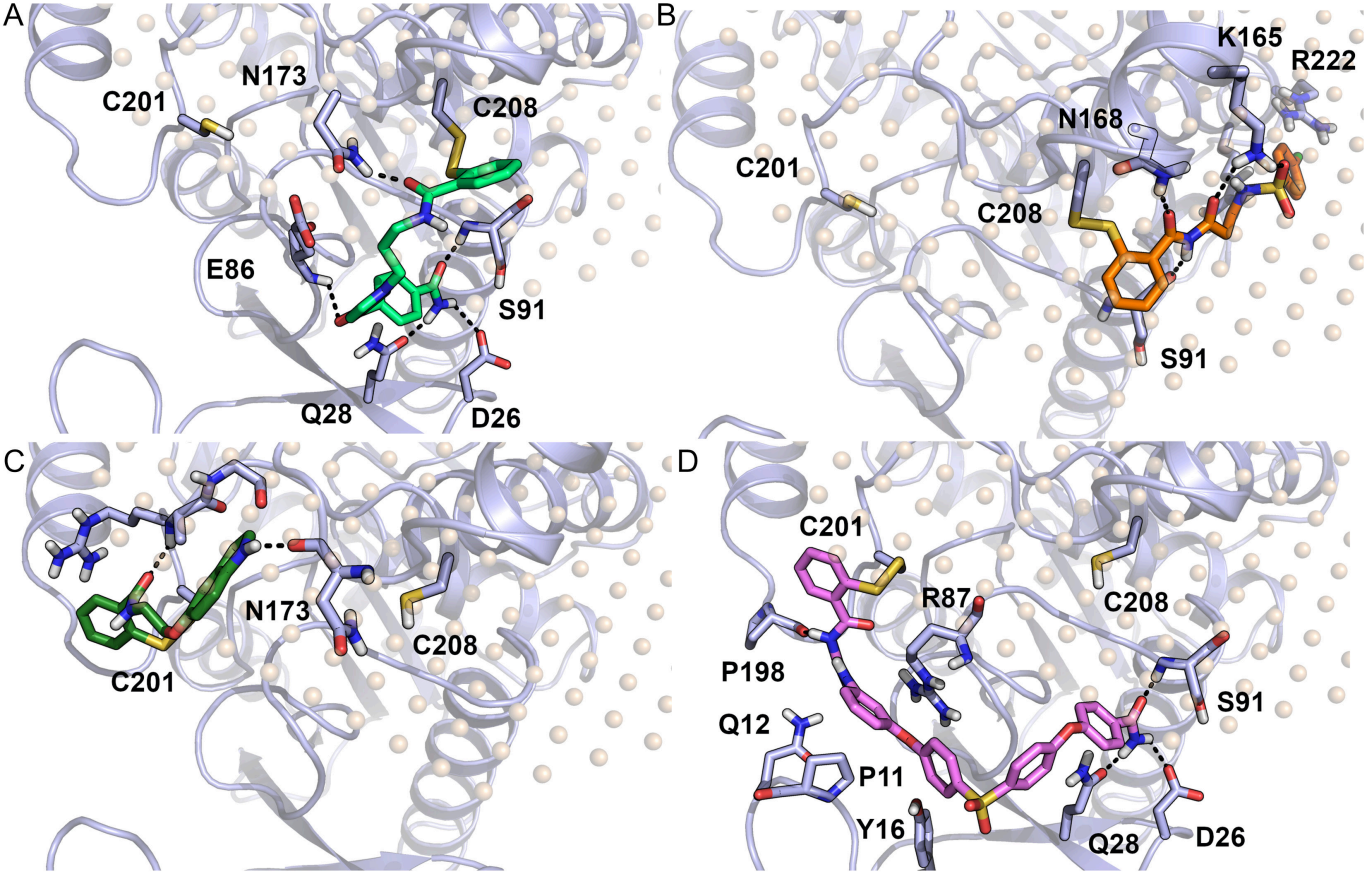
Covalent docking of compounds **23** (green carbon atoms, panel A), **2c** (orange carbon atoms, panel B), **10** (dark‐green carbon atoms, panel C), and **14** (pink carbon atoms**,** panel D) on Cys208 (panels A and B) or Cys201 (panels C and D) of human MGL (PDB 3HJU). The protein is represented in light‐blue cartoon, and beige spheres represent the positions of the phosphate groups of the membrane bilayer. Hydrogen bonds are shown as black dashed lines.

Compounds **22**, **23**, and **2c** were designed to form a covalent adduct with Cys208. Analysis of docking poses obtained for **22** and **23** showed a similar binding mode in which the amide group, generated upon BTZ ring opening, established polar interactions with the side chain of Asn173. The *N*‐propyl‐ and *N*‐ethyl‐pyrrole moieties were oriented toward the space beneath Cys208, allowing the benzamide moiety to extend into and engage with a small hydrophilic cleft defined by Asp26, Gln28, and Ser91 (Figure 5A). Compound **2c** assumed a different binding mode, closer to the membrane and oriented toward a basic groove, forming a covalent adduct stabilized by polar contacts with residues on helix α4 (Lys165 and Asn168) and helix α6 (Arg219 and Arg222) (Figure 5B). The two pyrrolic derivatives unfortunately failed to establish productive interactions with MGL, displaying reduced potency, whereas **2c** proved to be slightly more potent than **UPR1218**.

Compounds designed to target Cys201—namely the shorter **10** and the more elongated **14** and **15**—were conceived to adopt two distinct binding orientations. Compound **10** occupied the region between Cys201 and Cys208, positioning its indole ring within the shallow cleft bordered by Asn173, Ala203, and Gly204, thereby enabling the formation of a hydrogen bond between the indole NH and the backbone of Asn173 (Figure 5C). The elongated scaffolds of **14** and **15** positioned the amide group, generated upon BTZ opening, within a small cavity that is occupied by water molecules in crystal structures. The central portion of the molecule lies along a superficial channel bordered by Pro11, Gln12, Tyr16, and Arg87, allowing the terminal benzimidazole (**15**) and benzamide (**14**) fragments to extend into and engage the same hydrophilic cleft described for **22** and **23** (Figure 5D). However, none of these compounds showed improved inhibitory potency.

Several factors may account for the failure of compounds designed to establish multiple, specific interactions with MGL. On the one hand is the intrinsic difficulty of engaging a protein surface that is largely devoid of well‐defined regions, with shape and polarity suitable for achieving strong stereoelectronic complementarity. On the other hand, the covalent docking approach prioritized the formation of interactions with the protein after disulfide adduct formation, with the aim—at least in our design strategy—of maintaining a stable interaction. This approach might have overlooked the relevance of productive interactions between the intact BTZ and MGL prior to the covalent bond formation. In fact, the formation of specific interactions during the initial approach and recognition between inhibitor and protein may be more relevant; yet, the nature and location of such interactions remain even less predictable.

To evaluate whether the substituents introduced on the BTZ nitrogen atom could facilitate the initial recognition of MGL and promote positioning of the reactive warhead in proximity to the target cysteine, molecular dynamics (MD) simulations were carried out on the complexes of MGL with compound **16**, lacking a dedicated recognition moiety, and compound **15**, designed to establish polar interactions with residues on the MGL surface. Starting from the covalent docking poses, the disulfide bond with Cys201 was cleaved and the benzisothiazolinone warhead was restored. The resulting noncovalent complexes were subjected to two independent 500 ns MD simulations for each compound.

In both cases, the complexes proved to be only moderately stable, with the inhibitors departing from the interaction region predicted by covalent docking within approximately 50–100 ns in both simulation replicas (Figure S3). Nevertheless, inspection of the early stages of the trajectories revealed distinct behaviors for the two compounds. In the case of **15**, the phenylsulfonylphenoxybenzimidazole recognition moiety remained associated with the MGL surface through polar interactions, whereas the reactive benzisothiazolinone warhead rapidly drifted away from Cys201 and only rarely returned to a geometry compatible with disulfide bond formation. These observations indicate that, although the recognition moiety can contribute to the initial association with the protein, the conformations adopted by the inhibitor during the early recognition phase are generally not favorable for productive interaction between the reactive sulfur atom and Cys201. In contrast, compound **16** displayed greater mobility on the protein surface but more frequently sampled conformations in which the sulfur atom of the BTZ warhead remained at shorter distances from Cys201, potentially increasing the probability of covalent adduct formation. Overall, these simulations highlight the intrinsic challenge of simultaneously achieving productive molecular recognition and optimal positioning of the reactive warhead when targeting shallow, solvent‐exposed protein surfaces.

In practice, inhibitor potency proved to be largely independent of the structure and properties of the substituent on the BTZ nitrogen atom, consistently clustering around ≈100 nM, as observed for the phenethyl derivative UPR1218. The observed differences in IC_50_ values are within the experimental variability (SEM) and therefore do not indicate meaningful differences in inhibitory potency among these derivatives. The most potent compounds, with IC_50_ values of approximately 50 nM irrespective of the different side chains, bear an acyl substituent (**2a**, **2b**, **2c**), in agreement with reactivity as the primary factor governing inhibitor potency. Supporting the predominant role of the reactive warhead, **16**, lacking a specific recognition moiety, was synthesized and turned out to be the most potent derivative (IC_50_ = 7.8 nM). Nevertheless, its inhibitory potency may still be partially influenced by favorable hydrogen‐bond interactions with residues located in the vicinity of Cys201, including Pro198, Arg87, and Gly81.

Overall, the BTZ class appears difficult to modulate in terms of both reactivity [41] and molecular recognition, posing inherent challenges for the rational design of more potent and, especially, selective MGL inhibitors. Given the essentially flat structure–activity relationships and the lack of any improvement in inhibitory potency over the reference compound **UPR1218**, these derivatives were not progressed to further biological characterization. Consequently, their selectivity toward potential off‐targets was not investigated.

## 3 Conclusion

Attempts to optimize the BTZ scaffold to retain high inhibitory potency while promoting selective action on MGL to reduce the likelihood of off‐target reactivity have yielded overall unsatisfactory results. On the one hand, the mechanism‐based approach produced two inactive compounds, although the strategy of releasing the (benz)isothiazolinone moiety through enzyme‐mediated activation in proximity to the cysteine residues involved in covalent adduct formation remains potentially viable. Further development may be achieved through the design of stable compounds with greater substrate‐like features to facilitate the hydrolytic reaction. On the other hand, efforts to decorate the BTZ core to enhance specific recognition by MGL did not result in improved potency, supporting the notion that the intrinsic reactivity of the heterocyclic ring is the predominant and only marginally tunable factor. Accordingly, the development of BTZ derivatives optimized for a specific target may represent a challenging and not readily attainable objective.

## 4 Experimental Section

### 4.1 Chemistry

#### 4.1.1 General Methods

Unless otherwise noted, reagents and solvents were purchased from commercial suppliers and were used without purification. The progress of the reactions was monitored by thin‐layer chromatography with F254 silica gel–precoated sheets (Merck, Darmstadt, Germany). UV light and a solution of ammonium molybdate and ceric sulfate in aqueous sulfuric acid (5% v/v) were used for detection. Silica gel column chromatography was performed using Merck silica gel 60 (Si 60, 40–63 μm, 230–400 mesh ASTM). Melting points were determined on a Buchi B‐540 capillary melting point apparatus. The ^1^H NMR and ^13^C NMR spectra were recorded on a Bruker Avance 400 spectrometer (400 MHz) or a JEOL ECZ600R (600 MHz) or a Bruker Avance Neo Ascend 600 (600 MHz); chemical shifts (*δ* scale) are reported in parts per million (ppm). ^1^H NMR spectra are reported in the following order: multiplicity, approximate coupling constants (*J* value) in Hertz (Hz) and number of protons; signals are characterized as s (singlet), d (doublet), dd (double doublets), ddd (doublet of doublet of doublets), t (triplet), q (quartet), m (multiplet), and brs (broad signal). Mass spectra were recorded on a Waters Micromass ZQ instrument or on Advion Expression CMS coupled with Plate Express TLC‐plate Reader. High‐resolution mass spectrometry (HRMS) analysis was performed on Orbitrap Exploris 240 Mass Spectrometer (Thermo Fisher Scientific) or Vanquish Flex UHPLC system (Thermo Scientific, Waltham, MA, USA) coupled to an Orbitrap Exploris 120 mass spectrometer (Thermo Scientific, Waltham, MA, USA), equipped with a heated electrospray ionization (H‐ESI) ion source. The purity of tested compounds, determined by HPLC, was greater than 95%. These analyses were performed on a Waters HPLC/DAD/MS system (separation module Alliance HT2795, Photo Diode Array Detector 2996, mass detector Micromass ZQ; software: MassLynx 4.1) or an Agilent 1260 Infinity II (Agilent, Santa Clara, CA, United States) equipped with a UV–Vis diode‐array detector (DAD, model G7115A).

#### 4.1.2 General Procedure for Acylation of Benzisothiazolinone

The suitable acyl chloride (0.5 mmol) was added to a stirred, ice–cooled mixture of benzisothiazolinone (75 mg, 0.5 mmol), ZnCl_2_ (70 mg, 0.5 mmol), and TEA (0.14 mL, 1 mmol) in anhydrous acetone (3 mL) monitoring reaction progress by TLC.

##### 4.1.2.1 Benzo[*d*]isothiazol‐3‐yl benzoate (1a) and 2‍‐‍benzoylbenzo[*d*]isothiazol‐3(2*H*)‐one (2a)

**1a** and **2a** were prepared by acylation of BTZ with benzoyl chloride. The reaction was stirred at 0 °C for 30 min and then partitioned between EtOAc and water. The combined organic phases were washed with brine, dried (Na_2_SO_4_), and concentrated under reduced pressure to give a crude residue that was purified by silica gel column chromatography (cyclohexane/EtOAc 95:5 as eluent) and crystallization.

**1a**. White solid, mp 76–77 °C (cyclohexane); 59 mg, 46% yield. ^1^H NMR (400 MHz, CDCl_3_): *δ* = 8.32–8.29 (m, 2H), 7.93 (ddd, *J*_1_ = 8.0, *J*_2_ = *J*_3_ = 1.0 Hz, 1H), 7.85 (ddd, *J*_1_ = 8.0, *J*_2_ = *J*_3_ = 1.0 Hz, 1H), 7.74–7.70 (m, 1H), 7.62–7.56 (m, 3H), 7.46 (ddd, *J* = 8.0, 7.0, 1.0 Hz, 1H). ^13^C NMR (150 MHz, CDCl_3_): *δ* = 163.8, 156.4, 152.7, 134.3, 130.6, 128.8, 128.7, 128.2, 126.5, 125.3, 122.9, 120.5. HPLC‐UV purity: *t*R 4.63 min, 96%. ESI MS (*m/z*): 256 [M+H]^+^. HRMS (ESI): *m/z* calcd for C_14_H_10_NO_2_S^+^: 256.0432 [*M* + H]^+^; found: 256.0424.

**2a**. White solid, mp 168–169 °C (EtOAc‐cyclohexane); 15 mg, 12% yield. ^1^H NMR (400 MHz, CDCl_3_): *δ* = 7.97 (ddd, *J* = 8.0, 1.5, 1.0 Hz, 1H), 7.79–7.74 (m, 2H), 7.73 (ddd, *J* = 8.0, 7.0, 1.0 Hz, 1H), 7.63–7.58 (m, 1H), 7.57 (ddd, *J* = 8.0, *J*_2_ = *J*_3_ = 1.0 Hz, 1H), 7.53–7.47 (m, 2H), 7.41 (ddd, *J* = 8.0, 7.0, 1.0 Hz, 1H). ^13^C NMR (150 MHz, CDCl_3_): *δ*= 169.0, 163.1, 141.5, 134.6, 133.0, 132.6, 129.1, 128.04, 128.01, 126.0, 125.2, 120.6. HPLC‐UV purity: *t*R 3.77 min, 98%. ESI MS (*m/z*): 278 [M+Na]^+^. HRMS (ESI): *m/z* calcd for C_14_H_9_NO_2_S+Na^+^: 278.0252 [*M* + Na]^+^; found: 278.0242.

##### 4.1.2.2 2‐Oleoylbenzo[*d*]isothiazol‐3(2*H*)‐one (2b)

**2b** was prepared by acylation of BTZ with oleoyl chloride. The reaction mixture was stirred at 0 °C for 10 min, the solvent was removed by distillation under reduced pressure, and the crude oily residue was purified by silica gel column chromatography (cyclohexane/EtOAc 95:5 as eluent). Amorphous solid; 77 mg, 37% yield. ^1^H NMR (400 MHz, CDCl_3_): *δ* = 8.02 (ddd, *J*_1_ = 8.0, *J*_2_ = *J*_3_ = 1.0 Hz, 1H), 7.70 (ddd, *J* = 8.0, 7.0, 1.0 Hz, 1H), 7.52 (ddd, *J*_1_ = 8.0, *J*_2_ = *J*_3_ = 1.0 Hz, 1H), 7.41 (ddd, *J* = 8.0, 7.0, 1.0 Hz, 1H), 5.39–5.31 (m, 2H), 3.17 (t, *J* = 7.5 Hz, 2H), 2.05–1.99 (m, 4H), 1.82–1.74 (m, 2H), 1.56–1.27 (m, 20H), 0.91–0.87 (m, 3H). ^13^C NMR (150 MHz, CDCl_3_): *δ* = 173.3, 163.5, 141.1, 134.3, 130.0, 129.8, 127.8, 125.8, 125.7, 120.6, 37.4, 31.9, 29.8, 29.7, 29.5, 29.4, 29.3, 29.2, 29.13, 29.12, 27.23, 27.20, 24.2, 22.7, 14.1. HPLC‐UV purity: *t*R 9.22 min, >95%. ESI MS (*m/z*): 438 [M+Na]^+^. HRMS (ESI): *m/z* calcd for C_25_H_38_NO_2_S^+^: 416.2623 [*M* + H]^+^; found: 416.2623.

##### 4.1.2.3 4‐Chloro‐*N*‐(3‐oxo‐3‐(3‐oxobenzo[*d*]isothiazol‐2(3*H*)‍‐‍yl)‍‍‍propyl)benzenesulfonamide (2c)

**2c** was prepared by acylation of BTZ with 3‐[(4‐chlorophenyl)sulfonamido]propanoyl chloride [48]. The reaction mixture was stirred at 0 °C for 30 min, the solvent was removed by distillation under reduced pressure, and the residue was partitioned between EtOAc and water. The combined organic phases were washed with brine, dried (Na_2_SO_4_), and concentrated under reduced pressure to give a crude residue that was purified by silica gel column chromatography (cyclohexane/EtOAc gradient from 8:2 to 6:4). White solid; 142 mg, 72% yield. ^1^H NMR (400 MHz, DMSO‐*d*_6_): *δ* = 8.02–7.96 (m, 3H), 7.87–7.82 (m 3H), 7.72–7.87 (m, 2H), 7.52 (ddd, 1H, *J* = 8.0, 7.0, 1.0 Hz), 3.30–3.25 (m, 2H), 3.18–3.14 (m, 2H). ^13^C NMR (150 MHz, DMSO‐*d*_6_): *δ* = 171.1, 163.5, 141.4, 139.7, 137.7, 135.1, 129.8, 128.9, 127.5, 126.6, 125.5, 122.6, 38.0, 37.6. HPLC‐UV purity: *t*R 3.80 min, 98%. ESI MS (*m/z*): 397 [M+H]‍‍‍‍^+^. HRMS (ESI): *m/z* calcd for C_16_H_14_ClN_2_O_4_S_2_^+^: 397.0084 [*M* + H]‍^+^; found: 397.0078.

#### 4.1.3 Ethyl 1‐benzhydrylpiperidine‐4‐carboxylate (3)

K_2_CO_3_ (0.88 g, 6.4 mmol) and benzhydryl bromide (1.57 g, 6.4 mmol) were added to a solution of ethyl piperidine‐4‐carboxylate (0.50 g, 3.2 mmol) in anhydrous DMF (16 mL), and the resulting mixture was stirred under nitrogen atmosphere for 18 h at 80 °C. After cooling to room temperature, the reaction was quenched with water and extracted with EtOAc. The combined organic phases were washed with brine, dried (Na_2_SO_4_), and concentrated under reduced pressure to give a crude residue that was purified by silica gel column chromatography (cyclohexane/EtOAc 98:2 as eluent). Oil; 517 mg, 50% yield. ^1^H NMR (400 MHz, CDCl_3_): *δ* = 7.42–7.40 (m, 4H), 7.29–7.25 (m, 4H), 7.20–7.16 (m, 2H), 4.25 (s, 1H), 4.13 (q, *J* = 7.0 Hz, 2H), 2.90–2.85 (m, 2H), 2.33–2.25 (m, 1H), 1.95–1.75 (m, 6H), 1.25 (t, *J* = 7.0 Hz, 3H). ^13^C NMR (150 MHz, CDCl_3_): *δ* = 175.4, 142.9, 128.4, 127.9, 126.8, 76.2, 60.2, 51.5, 41.5, 28.6, 14.2. ESI MS (*m/z*): 324 [M+H]^+^.

#### 4.1.4 1‐Benzhydrylpiperidine‐4‐carboxylic acid (4)

A solution of NaOH (380 mg, 9.5 mmol) in water (5.5 mL) was added to a solution of the ethyl 1‐benzhydrylpiperidine‐4‐carboxylate **3** (1.63 g, 4.74 mmol) in MeOH (11 mL) at room temperature. The reaction mixture was then heated at 60 °C for 18 h and, after cooling to room temperature, concentrated under reduced pressure, neutralized with 1N HCl aqueous solution, and extracted with EtOAc. The combined organic phases were washed with brine, dried (Na_2_SO_4_), and evaporated to give a crude residue that was purified by silica gel column chromatography (EtOAc as eluent) followed by crystallization. White solid, mp 157–158 °C (diethyl ether/cyclohexane); 1.34 g, 96% yield. ^1^H NMR (400 MHz, CDCl_3_): *δ* = 7.41–7.38 (m, 4H), 7.29–7.25 (m, 4H), 7.20–7.16 (m, 2H), 4.26 (s, 1H), 2.90–2.86 (m, 2H), 2.39–2.25 (m, 1H), 2.05–1.77 (m, 6H). ^13^C NMR (100 MHz, CDCl_3_): *δ* = 180.2, 142.7, 128.4, 127.9, 126.9, 76.1, 51.4, 40.9, 28.3. ESI MS (*m/z*): 296 [M+H]^+^.

#### 4.1.5 Chloromethyl 1‐benzhydrylpiperidine‐4‐carboxylate (5)

A solution of chloromethyl chlorosulfate (0.76 mL, 7.5 mmol) in DCM (10 mL) was added dropwise at room temperature to a solution of 1‐benzhydrylpiperidine‐4‐carboxylic acid **4** (1.47 g, 5 mmol), NaHCO_3_ (1.33 g, 15 mmol), and (*n‐*Bu)_4_NHSO_4_ (0.170 g, 0.5 mmol) in water (10 mL). The resulting mixture was vigorously stirred at room temperature for 18 h; then, the organic phase was washed with water, dried (Na_2_SO_4_), and concentrated under reduced pressure to give a crude residue that was purified by silica gel column chromatography (cyclohexane/Et_2_O gradient from 99:1 to 95:5). White solid; 892 mg, 52% yield. ^1^H NMR (400 MHz, CDCl_3_): *δ* = 7.41–7.38 (m, 4H), 7.30–7.25 (m, 4H), 7.21–7.16 (m, 2H), 5.71 (s, 2H), 4.26 (s, 1H), 2.90–2.85 (m, 2H), 2.42–2.34 (m, 1H), 1.97–1.77 (m, 6H). ^13^C NMR (100 MHz, CDCl_3_): *δ* = 173.3, 142.7, 128.4, 127.8, 126.9, 76.1, 68.6, 51.2, 41.1, 28.1. ESI MS (*m/z*): 344 [M+H]^+^.

#### 4.1.6 (Isothiazol‐3‐yloxy)methyl 1‐benzhydrylpiperidine‐4‍‐‍carboxylate (6)

A solution of **5** (450 mg, 1.3 mmol) in DCM (0.8 mL) was added to a mixture of (*n‐*Bu)_4_NHSO_4_ (220 mg, 0.65 mmol) in DCM (0.8 mL) and isothiazol‐3(2*H*)‐one (66 mg, 0.65 mmol) and K_2_CO_3_ (270 mg, 1.95 mmol) in water (1.65 mL). After stirring at room temperature for 20 h, the reaction mixture was diluted with water and extracted with DCM. The combined organic phases were washed with brine, dried (Na_2_SO_4_), and concentrated under reduced pressure to give a crude residue that was purified by silica gel column chromatography (cyclohexane/EtOAc gradient from 95:5 to 7:3). White solid; 77 mg, 29% yield. ^1^H NMR (400 MHz, CDCl_3_): *δ* = 8.48 (d, *J* = 5.0 Hz, 1H), 7.40–7.38 (m, 4H), 7.28–7.24 (m, 4H), 7.20–7.16 (m, 2H), 6.65 (d, *J* = 5.0 Hz, 1H), 6.06 (s, 2H), 4.23 (s, 1H), 2.88–2.83 (m, 2H), 2.42–2.35 (m, 1H), 1.94–1.76 (m, 6H). ^13^C NMR (100 MHz, CDCl_3_): *δ* = 174.5, 167.3, 149.7, 143.0, 128.6, 128.0, 127.0, 112.1, 83.8, 76.3, 51.5, 41.3, 28.4. HPLC‐UV purity: *t*R 1.32 min, >99%. ESI MS (*m/z*): 409 [M+H]^+^. HRMS (ESI): *m/z* calcd for C_23_H_25_N_2_O_3_S^+^: 409.1586 [*M* + H]^+^; found: 409.1580.

#### 4.1.7 Methyl 2‐((1*H*‐indol‐5‐yl)oxy)acetate (7)

NaH (60% in mineral oil, 55 mg, 1.37 mmol) was added to a solution of 5‐hydroxyindole (166 mg, 1.25 mmol) in anhydrous DMF (10 mL) stirred under nitrogen atmosphere at −10 °C. After 30 min, methyl chloroacetate (0.22 mL, 2.5 mmol) was added to the reaction mixture and the stirring continued at room temperature for 16 h. The mixture was poured into ice water and extracted with EtOAc. The combined organic phases were washed with brine, dried (Na_2_SO_4_), and concentrated to give a crude residue that was purified by silica gel column chromatography (DCM/EtOAc 99:1 as eluent). Yellow solid; 154 mg, 60% yield. ^1^H NMR (400 MHz, CDCl_3_): *δ* = 8.26 (brs, 1H), 7.55 (d, *J* = 9.0 Hz, 1H), 7.09 (dd, *J*_1_ = *J*_2_ = 2.5 Hz, 1H), 6.87–6.84 (2H, m), 6.49 (dd, *J*_1_ = *J*_2_ = 2.5 Hz, 1H), 4.67 (s, 2H), 3.82 (s, 3H). ^13^C NMR (150 MHz, CDCl_3_): *δ* = 169.9, 154.6, 136.3, 123.6, 123.1, 121.4, 110.1, 102.5, 96.3, 66.4, 52.2. ESI MS (*m/z*): 206 [M+H]^+^.

#### 4.1.8 2‐((1*H*‐Indol‐5‐yl)oxy)ethan‐1‐ol (8)

A solution of methyl 2‐[(1*H*‐indol‐5‐yl)oxy]acetate **7** (167 mg, 0.81 mmol) in anhydrous THF (5 mL) was added dropwise under nitrogen to an ice‐cooled suspension of LiAlH_4_ (38 mg, 1.0 mmol) in anhydrous THF (5 mL). Upon completion of the addition, the mixture was stirred at 0 °C for 1.5 h, then H_2_O was carefully added to quench the excess of LiAlH_4_, and the resulting mixture was filtered through a pad of celite. The filtrate was concentrated in vacuo, and the residue was partitioned between H_2_O and EtOAc. The combined organic phases were washed with brine, dried (Na_2_SO_4_), and concentrated to yield the crude product that was purified by silica gel column chromatography (cyclohexane/EtOAc 6:4 as eluent). White solid; 107 mg, 75% yield. ^1^H NMR (400 MHz, CDCl_3_): *δ* = 8.09 (brs, 1H), 7.31 (d, *J* = 9.0 Hz, 1H), 7.21 (dd, *J*_1_ = *J*_2_ = 2.5 Hz, 1H), 7.14 (d, *J* = 2.5 Hz, 1H), 6.90 (dd, *J* = 9.0, 2.5 Hz, 1H), 6.50–6.49 (m, 1H), 4.16–4.10 (m, 3H), 4.00–3.98 (m, 2H).

#### 4.1.9 5‐(2‐Iodoethoxy)‐1*H*‐indole (9)

In an oven‐dried round bottom flask, under a flow of nitrogen, triphenylphosphine (184 mg, 0.70 mmol), imidazole (52 mg, 0.76 mmol), and anhydrous DCM (2.5 mL) were added and the resulting solution was stirred for 5 min at 0 °C. Iodine (178 mg, 0.70 mmol) was added to the solution, and the resulting mixture was stirred for 15 min under a nitrogen atmosphere. Then, 2‐[(1*H*‐indol‐5‐yl)oxy]ethan‐1‐ol **8** (104 mg, 0.59 mmol) was added to the reaction, and after 30 min at 0 °C, the stirring continued at room temperature for 24 h. The reaction was quenched with water and extracted with DCM. The combined organic phases were dried (Na_2_SO_4_), and the solvent was removed by distillation under reduced pressure to yield a crude residue that was purified by silica gel column chromatography (cyclohexane/EtOAc 85:15 as eluent). Oil; 162 mg, 96% yield. ^1^H NMR (400 MHz, CDCl_3_): *δ* = 8.09 (brs, 1H), 7.30 (d, *J* = 9.0 Hz, 1H), 7.21 (dd, *J* = 2.5, 3.0 Hz, 1H), 7.14 (d, *J* = 2.5 Hz, 1H), 6.89 (dd, *J* = 9.0, 2.5 Hz, 1H), 6.50–6.49 (m, 1H), 4.30 (t, *J* = 7.0 Hz, 2H), 3.45 (t, *J* = 7.0 Hz, 2H). ESI MS (*m/z*): 288 [M+H]^+^.

#### 4.1.10 2‐(2‐((1*H*)Indol‐5‐yl)oxy)ethylbenzo[*d*]isothiazol‐3(2*H*)‐one (10)

5‐(2‐Iodoethoxy)‐1*H*‐indole **9** (138 mg, 0.48 mmol) was added to a suspension of benzisothiazolinone (73 mg, 0.48 mmol) and K_2_CO_3_ (66 mg, 0.48 mmol) in CH_3_CN (5 mL), and the resulting mixture was refluxed 24 h under nitrogen atmosphere. After removing the solvent by distillation under reduced pressure, the residue was treated with water and extracted with DCM. The combined organic phases were washed with brine, dried (Na_2_SO_4_), and concentrated in vacuo to yield a crude product that was purified by silica gel column chromatography (cyclohexane/EtOAc gradient from 8:2 to 6:4). Orange solid; 52 mg, 35% yield. ^1^H NMR (400 MHz, CDCl_3_): *δ* = 8.08 (brs, 1H), 8.06 (ddd, *J*_1_ = 8.0, *J*_2_ = *J*_3_ = 1.0 Hz, 1H), 7.63–7.54 (m, 2H), 7.40 (ddd, *J* = 8.0, 7.0, 1.0 Hz, 1H), 7.31 (d, *J* = 9.0 Hz, 1H), 7.20 (dd, *J*_1_ = *J*_2_ = 3.0 Hz, 1H), 7.16 (d, *J* = 2.5 Hz, 1H), 6.94 (dd, *J* = 9.0, 2.5 Hz, 1H), 6.49–6.47 (m, 1H), 4.36–4.32 (m, 4H). ^13^C NMR (100 MHz, CDCl_3_): *δ* = 165.5, 152.6, 141.3, 131.8, 131.4, 128.3, 126.6, 125.3, 125.1, 124.1, 120.2, 112.7, 111.7, 104.1, 102.5, 67.6, 43.7. HPLC‐UV purity: *t*R 3.22 min, >99%. ESI MS (*m/z*): 311 [M+H]^+^. HRMS (ESI): *m/z* calcd for C_17_H_15_N_2_O_2_S^+^: 311.0854 [*M* + H]^+^; found: 311.0844.

#### 4.1.11 3‐(4‐((4‐Fluorophenyl)sulfonyl)phenoxy)aniline (11)

Cs_2_CO_3_ (1.89 g, 5.8 mmol) was added under nitrogen atmosphere to a solution of 4,4′‐sulfonylbis(fluorobenzene) (2.21 g, 8.7 mmol) and 3‐amino phenol (0.316 g, 2.9 mmol) in anhydrous DMF (13 mL). The reaction mixture was heated at 60 °C for 2 h, then cooled to room temperature, quenched with water, and extracted with EtOAc. The combined organic phases were washed with brine, dried (Na_2_SO_4_), and evaporated to give a crude residue that was purified by silica gel column chromatography (DCM/Et_2_O 98:2 as eluent). White solid; 2.57 g, 86% yield. ^1^H NMR (400 MHz, CDCl_3_): *δ* = 7.97–7.93 (m, 2H), 7.88–7.84 (m, 2H), 7.21–7.16 (m, 3H), 7.06–7.02 (m, 2H), 6.63 (ddd, *J* = 8.0, 2.0, 1.0 Hz, 1H), 6.49 (ddd, *J* = 8.0, 2.0, 1.0 Hz, 1H), 6.47 (dd,, *J*_1_ = *J*_2_ = 2.0 Hz, 1H). ^13^C NMR (150 MHz, CDCl_3_): *δ* = 165.3 (d, *J* = 254.0 Hz), 162.3, 156.0, 148.4, 138.2 (d, *J* = 3.2 Hz), 134.6, 130.8, 130.2 (d, *J* = 9.2 Hz), 129.8, 117.8, 116.5 (d, *J* = 22.2 Hz), 111.8, 110.1, 106.8. ESI MS (*m/z*): 344 [M+H]^+^.

#### 4.1.12 4‐(4‐((4‐(3‐‍‍‍‍Aminophenoxy)phenyl)sulfonyl)phenoxy)benzamide (12)

Cs_2_CO_3_ (0.71 g, 2.2 mmol) was added under nitrogen atmosphere to a solution of aniline **11** (0.41 g, 1.2 mmol) and 4‐hydroxybenzamide (0.15 g, 1.1 m‍mol) in anhydrous DMF (5 mL). The reaction mixture was heated at 60 °C for 2 h; then, it was diluted with water and extracted with EtOAc. The combined organic phases were washed with brine, dried (Na_2_SO_4_), and concentrated in vacuo to give a crude residue that was purified by silica gel column chromatography (DCM/MeOH 95:5 as eluent). White solid; 334 mg, 66% yield. ^1^H NMR (400 MHz, acetone‐*d*_6_): *δ* = 8.08–8.02 (m, 4H), 8.01–7.97 (m, 2H), 7.49 (brs, 1H), 7.25–7.19 (m, 4H), 7.16–7.12 (m, 3H), 6.65 (brs, 1H), 6.59 (ddd, *J* = 8.0, 2.0, 1.0 Hz, 1H), 6.43 (dd, *J*_1_ = *J*_2_ = 2.0 Hz, 1H), 6.33 (ddd, *J* = 8.0, 2.0, 1.0 Hz, 1H), 4.93 (brs, 2H). ^13^C NMR (150 MHz, DMSO‐*d*_6_): *δ* = 167.4, 162.2, 161.0, 157.6, 155.9, 151.3, 136.4, 135.1, 131.3, 130.9, 130.4, 130.3, 130.2, 119.9, 119.0, 118.2, 111.3, 107.3, 105.4. ESI MS (*m/z*): 461 [M+H]^+^.

#### 4.1.13 3‐(4‐((4‐((1*H*‐Benzo[*d*]imidazole‐6‐ol)oxy)phenyl)sulfonyl)phenoxy)aniline (13)

Cs_2_CO_3_ (2.35 g, 7.2 mmol) was added under nitrogen atmosphere to a solution of aniline **11** (0.41 g, 1.2 mmol) and 1*H*‐benzo[*d*]imidazole‐6‐ol (482 mg, 3.6 mmol) in anhydrous DMF (5 mL). The reaction mixture was heated at 60 °C for 2 h; then, it was diluted with water and extracted with EtOAc. The combined organic phases were washed with brine, dried (Na_2_SO_4_), and concentrated in vacuo to give a crude residue that was purified by two consecutive silica gel column chromatography (EtOAc/MeOH 95:5 as eluent). White solid; 132 mg, 24% yield. ^1^H NMR (400 MHz, acetone‐*d*_6_): *δ* = 8.08 (s, 1H), 7.83–7.78 (m, 4H), 7.55 (d, *J* = 8.5 Hz, 1H), 7.24 (d, *J* = 2.5 Hz, 1H), 7.00–6.93 (m, 5H), 6.88 (dd, *J* = 8.5, 2.5 Hz, 1H), 6.41 (ddd, *J* = 8.0, 2.0, 1.0 Hz, 1H), 6.25 (dd, *J*_1_ = *J*_2_ = 2.0 Hz, 1H), 6.16 (ddd, *J* = 8.0, 2.0, 1.0 Hz, 1H), 4.75 (brs, 2H). ESI MS (*m/z*): 458 [M+H]^+^.

#### 4.1.14 General Procedure for the Synthesis of Compounds 14 and 15

A solution of 2‐(3‐oxobenzo[*d*]isothiazol‐2(3*H*)‐yl)acetic acid (50 mg, 0.24 mmol), EDCI (48 mg, 0.31 mmol), and HOBT (35 mg, 0.26 mmol) in DMF (1.2 mL) was stirred for 15 min at room temperature. Then, intermediate **12** or **13** (0.29 mmol) was added, and the reaction was stirred for the indicated times (16 h at 60 °C for compound **14** and 6 h at room temperature for **15**). The reaction was quenched with water and extracted with EtOAc. The combined organic layers were washed with water and brine, dried over Na_2_SO_4_, and concentrated by distillation to give a crude residue that was purified by silica gel column chromatography (eluent: EtOAc for **14** and EtOAc/MeOH 95:5 for **15**).

##### 4.1.14.1 4‐(4‐((4‐(3‐(2‐(3‐Oxobenzo[*d*]isothiazol‐‍‍‍‍‍‍2(3*H*)‐yl)acetamido)phenoxy)phenyl)sulfonyl)phenoxy)benzamide (14)

White solid; 45 mg, 29% yield. ^1^H NMR (400 MHz, DMSO‐*d*_6_): *δ* = 10.49 (s, 1H), 8.00–7.92 (m, 8H), 7.89 (ddd, *J* = 8.0, *J*_2_ = *J*_3_ = 1.0 Hz, 1H), 7.71 (ddd, *J* = 8.0, 2.0, 1.0 Hz, 1H), 7.47–7.40 (m, 4H), 7.38 (brs, 1H), 7.20–7.13 (m, 6H), 6.88–6.85 (m, 1H), 4.67 (s, 2H). ^13^C NMR (100 MHz, DMSO‐*d*_6_): *δ* = 167.4, 166.2, 165.5, 161.6, 161.1, 157.5, 155.3, 141.9, 140.8, 136.2, 135.7, 132.5, 131.3, 131.2, 130.4, 126.1, 125.9, 123.8, 122.6, 119.9, 119.0, 118.6, 116.0, 115.6, 111.0, 46.6. HPLC‐UV purity: *t*R 3.35 min, >99%. ESI MS (*m/z*): 652 [M+H]^+^. HRMS (ESI): *m/z* calcd for C_34_H_26_N_3_O_7_S_2_^+^: 652.1212 [*M* + H]^+^; found: 652.1206.

##### 4.1.14.2 *N*‐(3‐(4‐((4‐((1*H*‐Benzo[*d*]imidazol‐6‐yloxy)phenyl)sulfonyl)phenoxy)phenyl)‐2‐(3‐oxobenzo[*d*]isothiazol‐2(3*H*)‐yl)acetamide (15)

White solid; 98 mg, 63% yield. ^1^H NMR (400 MHz, DMSO‐*d*_6_): *δ* = 12.66 (brs, 1H), 10.47 (s, 1H), 8.30 (s, 1H), 7.98 (ddd, *J* = 8.0, *J*_2_ = *J*_3_ = 1.0 Hz, 1H), 7.94–7.88 (m, 5H), 7.71 (ddd, *J* = 8.0, 2.0, 1.0 Hz, 1H), 7.66 (d, *J* = 8.5 Hz, 1H), 7.45 (ddd, *J* = 8.0, 2.0, 1.0 Hz, 1H), 7.44–7.40 (m, 3H), 7.37 (d, *J* = 2.5 Hz, 1H), 7.16–7.12 (m, 2H), 7.09–7.05 (m, 2H), 6.99 (dd, *J* = 8.5, 2.5 Hz, 1H), 6.87–6.84 (m, 1H), 4.67 (s, 2H). ^13^C NMR (150 MHz, DMSO‐*d*_6_): *δ* = 166.2, 165.5, 163.1, 161.5, 155.4, 149.9, 143.7, 141.9, 140.8, 139.0, 136.0, 134.9, 132.5, 131.2, 130.3, 126.1, 125.9, 123.8, 122.2, 118.6, 117.6, 117.0, 116.0, 115.5, 111.0, 107.7, 46.6. HPLC‐UV purity: *t*R 8.00 min, >99%. ESI MS (*m/z*): 649 [M+H]^+^. HRMS (ESI): *m/z* calcd for C_34_H_25_N_4_O_6_S_2_^+^: 649.1216 [M+H]^+^; found: 649.1201.

#### 4.1.15 2‐(3‐Oxobenzo[*d*]isothiazol‐2(3*H*)‐yl)acetamide (16)

Oxalyl chloride (0.060 mL, 0.72 mmol) and one drop of anhydrous DMF were added to a solution of 2‐(3‐oxobenzo[*d*]isothiazol‐2(3*H*)‐yl)acetic acid (100 mg, 0.48 mmol) in anhydrous DCM (1.5 mL) at 0 °C, and the resulting reaction mixture was stirred 2 h at room temperature. The solvent and the excess oxalyl chloride were removed by distillation under reduced pressure. The residue was dissolved in DCM (2 mL), a concentrated aqueous solution of ammonia was added until basic pH was achieved, and the resulting mixture was vigorously stirred at room temperature for 15 min. The organic solvent was concentrated and the residue partitioned between EtOAc and water. The combined organic phases were washed with brine, dried (Na_2_SO_4_), and concentrated by distillation in vacuo to give a crude residue that was purified by silica gel column chromatography (EtOAc/MeOH 97:3) followed by crystallization. White solid, mp 226–228 °C dec. (MeOH); 20 mg, 20% yield. ^1^H NMR (400 MHz, DMSO‐*d*_6_): *δ* = 7.99 (ddd, *J* = 8.0, *J*_2_ = *J*_3_ = 1.0 Hz, 1H), 7.92 (ddd, *J* = 8.0, *J*_2_ = *J*_3_ = 1.0 Hz, 1H), 7.73 (ddd, *J* = 8.0, 7.0, 1.0 Hz, 1H), 7.65 (brs, 1H), 7.47 (ddd, *J* = 8.0, 7.0, 1.0 Hz, 1H), 7.29 (brs, 1H), 4.45 (s, 2H). ^13^C NMR (150 MHz, DMSO‐*d*_6_): *δ* = 169.0, 165.3, 141.8, 132.3, 126.1, 125.7, 124.0, 122.1, 45.7. HPLC‐UV purity: *t*R 2.05 min, >99%. ESI MS (*m/z*): 209 [M+H]^+^. HRMS (ESI): *m/z* calcd for C_9_H_8_N_2_O_2_S+Na^+^: 231.0204 [*M* + Na]^+^; found: 231.0197.

#### 4.1.16 1‐Tosyl‐1*H*‐pyrrole (17)

To a stirred solution of pyrrole (1.00 g, 15 mmol) in anhydrous THF (20 mL) at 0 °C, sodium hydride (60% in mineral oil, 1.79 g, 45 mmol) was slowly added, and the resulting mixture was stirred at 0 °C for 15 min. Then, a solution of tosyl chloride (3.41 g, 18 mmol) in anhydrous THF (3 mL) was added dropwise at 0 °C, and the resulting reaction mixture was stirred at room temperature for 1 h. Water was slowly added at 0 °C, and the mixture was partitioned between water and EtOAc. The combined organic phases were washed with brine, dried (Na_2_SO_4_), and concentrated by distillation in vacuo to give a crude residue that was purified by silica gel column chromatography (cyclohexane/EtOAc 9:1 as eluent). White solid; 2.82 g, 85% yield. ^1^H NMR (400 MHz, CDCl_3_): *δ* = 7.74 (d, *J* = 8.3 Hz, 2H), 7.28 (d, *J* = 8.2 Hz, 2H), 7.19–7.13 (m, 2H), 6.30–6.26 (m, 2H), 2.39 (s, 3H).^13^C NMR (101 MHz, CDCl_3_): *δ* = 145.04, 136.28, 130.06, 126.92, 120.81, 113.60, 21.67. HPLC‐UV purity: *t*R 12.2 min, >99%. HRMS (ESI): *m/z* calcd for C_11_H_12_NO_2_S^+^: 222.0589 [*M* + H]^+^; found: 222.0584.

#### 4.1.17 4‐(1‐Tosyl‐1*H*‐pyrrole‐3‐carbonyl)benzonitrile (18)

To a stirred solution of 4‐cyanobenzoyl chloride (0.89 g, 5.4 mmol) in anhydrous DCM (7.5 mL) under nitrogen atmosphere, AlCl_3_ (1.20 g, 9.0 mmol) was slowly added, and the mixture was stirred for 15 min. Then, a solution of **17** (1.00 g, 4.5 mmol) in anhydrous DCM (7.5 mL) was added dropwise over 6 h. Water was slowly added, and the mixture was partitioned between water and EtOAc. The combined organic phases were washed with brine, dried (Na_2_SO_4_), and concentrated by distillation in vacuo to give a crude residue that was purified by silica gel column chromatography (cyclohexane/EtOAc 85:15 as eluent). White solid; 346 mg, 22% yield. ^1^H NMR (600 MHz, CDCl_3_): *δ* = 7.89–7.84 (m, 2H), 7.82–7.76 (m, 4H), 7.62 (t, *J* = 1.8 Hz, 1H), 7.34 (d, *J* = 8.1 Hz, 2H), 7.22 (dd, *J* = 3.3, 2.1 Hz, 1H), 6.77 (dd, *J* = 3.3, 1.6 Hz, 1H), 2.43 (s, 3H). ^13^C NMR (151 MHz, CDCl_3_): *δ* = 188.47, 146.44, 142.17, 134.99, 132.55, 130.59, 129.45, 127.49, 127.17, 126.43, 122.07, 118.10, 115.83, 113.80, 21.86. HPLC‐UV purity: *t*R 12.6 min, 97%. HRMS (ESI): *m/z* calcd for C_19_H_15_N_2_O_3_S^+^: 351.0798 [*M* + H]^+^; found: 351.0800.

#### 4.1.18 4‐(1*H*‐Pyrrole‐3‐carbonyl)benzamide (19)

To a stirred solution of **18** (343 mg, 1.0 mmol) in DMSO/MeOH (9:1, 3 mL) at 0 °C, H_2_O_2_ (0.80 mL, 9.2 mmol) was added dropwise, followed by NaOH (184 mg, 4.6 mmol). The mixture was stirred at 0 °C for 30 min; then, 1 M HCl was added to quench the reaction. The volatile materials were removed under reduced pressure, and the crude residue was purified by silica gel column chromatography (DCM/MeOH 95:5 as eluent). White solid; 184 mg, 86% yield. ^1^H NMR (400 MHz, DMSO‐*d*_6_): *δ* = 11.65 (s, 1H), 8.11 (s, 1H), 8.01–7.93 (m, 2H), 7.82–7.72 (m, 2H), 7.50 (s, 1H), 7.39 (dt, *J* = 3.2, 1.7 Hz, 1H), 6.93 (td, *J* = 2.6, 1.8 Hz, 1H), 6.55 (td, *J* = 2.6, 1.5 Hz, 1H). ^13^C NMR (151 MHz, DMSO‐*d*_6_): *δ* = 188.94, 167.32, 142.16, 136.48, 128.18, 127.48, 126.41, 123.42, 120.27, 109.17. HPLC‐UV purity: *t*R 7.1 min, 99%. HRMS (ESI): *m/z* calcd for C_12_H_11_N_2_O_2_^+^: 215.0815 [*M* + H]^+^; found: 215.0812.

#### 4.1.19 2‐(2‐Bromoethyl)benzo[*d*]isothiazol‐3(2*H*)‐one (20)

To a solution of benzisothiazolinone (1.00 g, 6.6 mmol) in DMF (20 mL), potassium carbonate (1.82 g, 13 mmol) was added at room temperature. Then, 1,2‐dibromoethane (0.74 mL, 8.6 mmol) was added, and the mixture was stirred at 60 °C for 1 h. Water was slowly added, and the mixture was extracted with EtOAc. The combined organic phases were washed with brine, dried (Na_2_SO_4_), and concentrated by distillation in vacuo to give a crude residue that was purified by silica gel column chromatography (cyclohexane/EtOAc 85:15 as eluent). White solid; 135 mg, 8% yield. ^1^H NMR (600 MHz, CDCl_3_): *δ* = 8.05 (m, 1H), 7.63 (m, 1H), 7.56 (m, 1H), 7.46–7.39 (m, 1H), 4.27 (t, *J* = 6.6 Hz, 2H), 3.65 (t, *J* = 6.6 Hz, 2H). ^13^C NMR (151 MHz, CDCl_3_): *δ* = 165.65, 140.63, 132.32, 126.95, 125.81, 124.08, 120.52, 45.81, 29.27. HPLC‐UV purity: *t*R 10.0 min, 99%. HRMS (ESI): *m/z* calcd for C_9_H_9_BrNOS^+^: 257.9583 [*M* + H]^+^; found: 257.9580.

#### 4.1.20 2‐(3‐Bromopropyl)benzo[*d*]isothiazol‐3(2*H*)‐one (21)

To a solution of benzisothiazolinone (1.00 g, 6.6 mmol) in DMF (20 mL), potassium carbonate (1.82 g, 13.2 mmol) was added at room temperature. Then, 1,3‐dibromopropane (0.97 mL, 8.6 mmol) was added, and the mixture was stirred at 60 °C for 1 h. Then, water was slowly added, and the mixture was extracted with EtOAc. The combined organic phases were washed with brine, dried (Na_2_SO_4_), and concentrated by distillation in vacuo to give a crude residue that was purified by silica gel column chromatography (cyclohexane/EtOAc 85:15 as eluent). White solid; 232 mg, 13% yield. ^1^H NMR (600 MHz, CDCl_3_): *δ* = 8.04 (m, 1H), 7.62 (m, 1H), 7.56 (m, 1H), 7.41 (m, 1H), 4.05 (t, *J* = 6.7 Hz, 2H), 3.47 (t, *J* = 6.5 Hz, 2H), 2.37–2.30 (m, 2H). ^13^C NMR (151 MHz, CDCl_3_): *δ* = 165.66, 140.41, 132.08, 126.84, 125.78, 124.61, 120.51, 42.62, 32.30, 30.05. HPLC‐UV purity: *t*R 10.6 min, 99%. HRMS (ESI): *m/z* calcd for C_10_H_11_BrNOS^+^: 271.9739 [*M* + H]^+^; found: 271.9736.

#### 4.1.21 4‐(1‐(2‐(3‐Oxobenzo[*d*]isothiazol‐2(3*H*)‐yl)ethyl)‐1*H*‐pyrrole‐3‐carbonyl)benzamide (22)

NaH (60% in mineral oil, 17.3 mg, 0.43 mmol) was added to an ice‐cooled solution of **19** (62.0 mg, 0.30 mmol) in DMF (10 mL) under nitrogen atmosphere, and the mixture was stirred for 15 min at 0 °C. Then, **20** (75 mg, 0.30 mmol) was added, and the mixture was stirred at room temperature for 1 h. After addition of water, the mixture was extracted with EtOAc, and the combined organic phases were washed with brine, dried (Na_2_SO_4_), and concentrated by distillation in vacuo. The crude residue was purified by silica gel column chromatography (DCM/MeOH 95:5 as eluent). White solid; 8.2 mg, 7% yield. ^1^H NMR (600 MHz, DMSO‐*d*_6_): *δ* = 8.00 (s, 1H), 7.96 (d, *J* = 8.2 Hz, 1H), 7.86 (d, *J* = 7.8 Hz, 1H), 7.84 (d, *J* = 8.1 Hz, 2H), 7.68 (t, *J* = 7.7 Hz, 1H), 7.60 (d, *J* = 8.0 Hz, 2H), 7.44 (t, *J* = 7.5 Hz, 1H), 7.38 (s, 1H), 7.30 (t, *J* = 2.4 Hz, 1H), 6.92 (t, *J* = 2.5 Hz, 1H), 6.51 (t, *J* = 2.3 Hz, 1H), 4.32 (t, *J* = 5.7 Hz, 2H), 4.22 (t, *J* = 5.6 Hz, 2H). ^13^C NMR (151 MHz, DMSO‐*d*_6_): *δ* = 188.48, 167.23, 164.46, 141.89, 140.88, 136.49, 131.96, 129.47, 128.02, 127.37, 125.61, 125.53, 123.60, 123.55, 123.54, 121.97, 109.86, 48.03, 43.94. HPLC‐UV purity: *t*R 8.5 min, 99%. HRMS (ESI): *m/z* calcd for C_21_H_18_N_3_O_3_S^+^: 392.1063 [*M* + H]^+^; found: 392.1057.

#### 4.1.22 4‐(1‐(3‐(3‐Oxobenzo[*d*]isothiazol‐2(3*H*)‐yl)propyl)‐1*H*‐pyrrole‐3‐carbonyl)benzamide (23)

NaH (60% in mineral oil, 7.50 mg, 0.19 mmol) was added to an ice‐cooled solution of **19** (25.0 mg, 0.12 mmol) in DMF (5 mL) under nitrogen atmosphere, and the mixture was stirred for 15 min at 0 °C. Then, **21** (34.0 mg, 0.12 mmol) was added, and the mixture was stirred at room temperature for 1 h. After addition of water, the mixture was extracted with EtOAc, and the combined organic phases were washed with brine, dried (Na_2_SO_4_), and concentrated by distillation in vacuo. The crude residue was purified by silica gel column chromatography (DCM/MeOH 95:5 as eluent). White solid; 12 mg, 25% yield. ^1^H NMR (400 MHz, DMSO‐*d*_6_): *δ* = 8.10 (s, 1H), 7.98 (m, 3H), 7.87 (d, *J* = 7.8 Hz, 1H), 7.79 (d, *J* = 8.3 Hz, 2H), 7.68 (m, 1H), 7.52 (t, *J* = 1.9 Hz, 1H), 7.50 (s, 1H), 7.44 (t, *J* = 7.4 Hz, 1H), 7.03 (dd, *J* = 2.9, 2.0 Hz, 1H), 6.54 (dd, *J* = 3.0, 1.7 Hz, 1H), 4.04 (t, *J* = 6.9 Hz, 2H), 3.83 (t, *J* = 6.8 Hz, 2H), 2.16 (p, *J* = 6.9 Hz, 2H). ^13^C NMR (101 MHz, DMSO‐*d*_6_): *δ* = 188.44, 167.27, 164.47, 141.98, 140.44, 136.47, 131.84, 129.28, 128.23, 127.48, 125.63, 125.55, 124.13, 123.22, 123.16, 121.95, 109.83, 46.52, 40.47, 30.67. HPLC‐UV purity: *t*R 9.1 min, 99%. HRMS (ESI): *m/z* calcd for C_22_H_20_N_3_O_3_S^+^: 406.1220 [*M* + H]^+^; found: 406.1213.

### 4.2 Molecular Modeling Studies

Molecular modeling studies were conducted using the Schrodinger 2023‐2 suite. The ligands were built in Maestro 11.6 [57] and prepared with Ligprep [58]. The protein structure was prepared and refined using the Protein Preparation Wizard tool. Docking and covalent docking studies were performed using Glide 7.9 [59, 60].

#### 4.2.1 Protein Preparation

The crystallographic structure of human MGL (PDB 3HJU, chain A) [53], with the lid domain in its open conformation, was used as starting model. Missing hydrogen atoms were added, and the network of intramolecular hydrogen bonds was optimized by sampling the conformation of histidine, asparagine, and glutamine side chains, as well as the orientation of hydroxyl and thiol groups. Basic and acidic amino acids were modeled in their charged forms, while histidine residues were maintained in their neutral form. The resulting structure was submitted to a restrained minimization conducted with the OPLS3e force field [61], during which only the hydrogen atoms were free to move. A second minimization was finally performed, in which also heavy atoms were free to move up to an RMSD value of 0.3 Å.

#### 4.2.2 Docking Studies

The docking grid was centered on the center of mass of residues Ala51, Ser122, Met123, and His269, and the inner and outer box dimensions were set, respectively, to 16 and 36 Å. Docking studies were performed using the standard precision (SP), applying all the parameters as default. Ten covalent docking poses were generated for each ligand, ranked according to their Gscore values. The top‐ranked poses were subjected to energy minimization to an energy gradient of 0.05 kJ mol^−1^Å^−1^, during which the coordinates of the protein α carbon atoms were fixed, performed using MacroModel [62] by applying OPLS3e force field.

To assess the suitability of the docking protocol, we evaluated its ability to reproduce the crystallographic binding mode of a reversible ligand bound to MGL in its open conformation. A benzoxazinone derivative cocrystallized with the human enzyme (PDB ID: 7L4T) [52, 53] was thus rebuilt in Maestro and redocked into the grid described above. The heavy‐atom root‐mean‐square deviation (RMSD) between the top‐ranked docking pose and the crystallographic ligand conformation was then calculated after alignment of the protein Cα atoms to the reference crystal structure. Noncovalent complexes of MGL with **1a** and **6** were generated applying the same protocol.

#### 4.2.3 Covalent Docking Studies

Covalent adducts between **22**, **23**, **2c**, **14**, **10**, and **15** and either Cys201 or Cys208 were generated using the covalent docking module of Glide [63], modeling disulfide bond formation under default parameters. Under these conditions, conformational sampling was restricted to the side chain of the cysteine involved in the covalent bond formation, while all other surrounding amino acid residues were held fixed. Ten covalent docking poses were generated for each ligand and ranked according to their GlideScore. The top‐ranked poses were subjected to energy minimization to an energy gradient of 0.05 kJ mol^−1^Å^−1^, during which the coordinates of the protein α carbon atoms were fixed, performed using MacroModel by applying OPLS3e force field.

To assess the ability of the covalent docking protocol to reproduce experimentally determined covalent binding modes of BTZ derivatives forming disulfide bonds with cysteine residues in different structural environments, two representative crystal structures were selected. A *Mycobacterium tuberculosis* transpeptidase Ldt_Mt2_ (PDB ID: 8A1K) [55], featuring a cysteine buried within a pocket shielded from the solvent, and a microbial urease (PDB ID: 9REY) [56], in which the reactive cysteine is solvent‐exposed, similar to Cys201 of MGL. The crystallographic ligands were redocked using the Glide Covalent Docking protocol under the same conditions described above, and the heavy‐atom RMSD values between the top‐ranked docking pose and the corresponding crystallographic adduct were calculated.

#### 4.2.4 MD Simulations of Noncovalent Complexes

MD simulations were performed to evaluate the stability of the complexes between MGL and the benzisothiazolinone derivative **15** or **16**. The noncovalent complexes were generated by cleaving the disulfide bond of the covalent adduct with Cys201 and restoring the intact benzisothiazolinone ring. The resulting complexes were energy‐minimized to an energy gradient of 0.05 kJ mol^−1^ Å^−1^ using MacroModel and the OPLS4 force field [64], while restraining the protein Cα atoms to their crystallographic coordinates. The minimized complexes were embedded in a POPC lipid bilayer according to the orientation of the enzyme reported in the Orientation of Proteins in Membranes (OPM) [65] database. The protein–membrane systems were subsequently solvated with approximately 12,200 TIP3P water molecules in a simulation box measuring 73 × 67 × 121 Å^3^. System neutrality was achieved by adding one Na^+^ ion. MD simulations were performed with Desmond 8.3 [66] using the OPLS4 force field. Bond lengths involving hydrogen atoms were constrained using the M‐SHAKE algorithm. Short‐range electrostatic interactions were truncated at 9 Å, whereas long‐range electrostatic interactions were treated using the Smooth Particle Mesh Ewald method. Integration of the equations of motion was performed using the RESPA multiple time‐step integrator with a 2 fs time step, while long‐range electrostatic interactions were evaluated every 6 fs. Each system was equilibrated for 20 ns by gradually increasing the temperature to 300 K while progressively releasing positional restraints on the heavy atoms. Two independent 500‐ns‐long MD simulations were performed for each ligand–protein complex in the NPγT ensemble using a Langevin thermostat.

### 4.3 MGL Enzyme Assay

In vitro MGL activity was measured as previously described with minor modifications [33, 67]. Briefly, *h*MGL (Cayman Chemicals, Ann Arbor, MI) was preincubated with the indicated concentrations of test compounds or vehicle (DMSO, 1% v/v) for 10 min at 37 °C in assay buffer (50 mM Tris−HCl, pH 8.0, containing 0.5 mg/mL fatty‐acid‐free BSA). The substrate 2‐oleoylglycerol (2‐OG) was then added to the mixture to a final concentration of 10 μM for a total reaction volume of 500 μL and incubated for an additional 30 min at 37 °C. Reactions were quenched by adding chloroform/methanol (2:1, v/v), containing 50 ng/mL of *cis*‐10‐heptadecenoic acid as an internal standard. After centrifugation at 2000 × g at 4 °C for 15 min, the bottom organic layer was collected and dried under a stream of N_2_. The lipid extracts were reconstituted in 250 μL of methanol and analyzed by LC−MS/MS.

An Agilent 1200 LC system coupled to a 6410 triple quadrupole MS system (Agilent Technologies, Santa Clara, CA, United States) was used for the analysis. The mobile phase consisted of 0.1% formic acid in water (A) and 0.1% formic acid in methanol (B). An Agilent Eclipse XDB C18 column (1.8 µm, 2.1 × 50 mm), equipped with a C18 guard column (2.1 × 3 mm), was used and thermostated at 40 °C. An isocratic elution with 87% solvent B was applied for 3.5 min, followed by a column flush with 95% solvent B for 1 min. The column was then reequilibrated to the initial conditions for 1.5 min, resulting in a total run time of 6 min. The flow rate was set to 0.3 mL/min. To minimize carryover, the injector needle was washed for 15 s in a solution of 10% acetone in water/methanol/isopropanol/acetonitrile (1:1:1:1, v/v/v/v) before each injection. MS detection was performed using ESI in negative ionization mode, with a capillary voltage of 4.0 kV and a fragmentor voltage of 120 V. Nitrogen was used as the drying gas at a flow rate of 12 L/min and a temperature of 330 °C. The nebulizer pressure was set at 35 psi. Quantification of o‍‍‍‍leic acid, the product of 2‐OG hydrolysis by MGL, was performed using an isotope dilution method, monitoring the [M – H]^−^ ion in the selected ion monitoring (SIM) mode for oleic acid (*m/z* 281.2) and *cis*‐10‍‐‍heptadecenoic acid (*m/z* 267.2). The linear calibration range was 5−2000 ng/mL, with a 1/x weighted *R* ^2^ of >0.98. Detection and analysis were conducted using MassHunter Workstation software (Agilent). IC_50_ values were calculated by nonlinear regression analysis using GraphPad Prism 10 (GraphPad Software Inc., CA, United States).

### 4.4 Stability of Compounds 1a and 6

Stability under enzyme‐free conditions was evaluated by incubating 100 nM of the compound under investigation in MGL assay buffer (final volume 500 µL) at 37 °C. Samples were collected at time 0 and after 40 min of incubation. Following incubation, chloroform/methanol (2:1, v/v) was added for liquid–liquid extraction. Samples were centrifuged (2000 × g, 4 °C, 15 min), and the lower organic phase was collected, dried under a stream of N_2_, and reconstituted in 500 μL of methanol prior to LC–MS/MS analysis. To evaluate hydrolysis by MGL, 100 nM of the compound under investigation was incubated with MGL under the same conditions used for the enzyme assay described above. Samples were collected at time 0 and after 40 min of incubation. Following reaction quenching, the organic phase was collected, dried under a stream of N_2_, and reconstituted in 500 μL of methanol prior to LC–MS/MS analysis.

An Agilent 1200 LC system coupled to a 6410 triple quadrupole MS system (Agilent Technologies, Santa Clara, CA, United States) was used for the analysis. The mobile phase consisted of 0.1% formic acid in water (A) and 0.1% formic acid in methanol (B). An Agilent Eclipse XDB C18 column (1.8 µm, 2.1 × 50 mm), equipped with a C18 guard column (2.1 × 3 mm), was used, both thermostated at 35 °C. **1a** was eluted using a gradient starting at 30% B and increasing to 95% B over 3 min, followed by 1 min at 95% B and reequilibration, for a total run time of 5 min. **6** was eluted using a gradient starting at 10% B and increasing to 95% B over 4 min, followed by 1 min at 95% B and reequilibration, for a total run time of 6 min. The flow rate was set at 0.3 mL/min. To minimize carryover, the injector needle was washed as described previously. MS detection was performed using ESI in positive ion mode, with a capillary voltage of 0.8 kV. Fragmentor voltages of 66 and 72 V and collision energies of 9 and 17 eV were used for **1a** and **6**, respectively. Nitrogen was used as the drying gas at a flow rate of 6 L/min and a temperature of 300 °C. The nebulizer pressure was set at 30 psi. Compound stability and hydrolysis were assessed by comparing LC–MS/MS detector responses at time 0 and after 40 min of incubation under the specified conditions, monitoring MRM transitions *m/z* 409.16 → 167.1 for **6** and *m/z* 256.1 → 105.0 for **1a**. Data acquisition and analysis were performed using MassHunter Workstation software (Agilent).

## Author Contributions

**Edoardo Rocca**: investigation, data curation, validation, writing – original draft. **Annalida Bedini**: investigation, data curation, writing – original draft. **Laura Scalvini**: investigation, data curation, formal analysis, visualization, writing – original draft. **Francesca Galvani**: investigation, data curation, formal analysis, validation, writing – review and editing. **Riccardo Castelli**: investigation, data curation, writing – review and editing. **Lorenzo Sarcone**: investigation, validation, writing – review and editing. **Adriano Recchia**: investigation, validation. **Fabiola Fanini**: investigation, validation. **Adren Tran**: investigation, validation. **Silvia Rivara**: project administration, supervision, writing – original draft. **Gilberto Spadoni**: conceptualization, supervision, writing – review and editing. **Daniele Piomelli**: conceptualization, writing – review and editing, supervision, funding acquisition. **Marco Mor**: conceptualization, supervision, writing – review and editing.

## Funding

NIH Project “Allosteric control of monoacylglyceride lipase (MGL) activity”; PTE federal award no. DA053358 to DP.

## Conflicts of Interest

The authors declare no conflicts of interest.

## Supporting information

Supplementary Material

## Data Availability

The data that support the findings of this study are available in the supplementary material of this article.
