# Supplementary material for: Iso‐ and Benzisothiazolinone Inhibitors of Monoacylglycerol Lipase: Exploring On‐Target Activity Through Scaffold Decoration and In Situ Warhead Generation
Source: ChemMedChem. 2026 Aug 25;21(16):e70414. doi: 10.1002/cmdc.70414 (PMC13504407; doi:10.1002/cmdc.70414)
Supplement: Supplementary file 1 [file CMDC-21-e70414-s001.pdf]

Iso- and Benzisothiazolinone Inhibitors of Monoacylglycerol Lipase: Exploring On-Target Activity through Scaffold Decoration and In Situ Warhead Generation

Edoardo Rocca,[1] Annalida Bedini,[2] Laura Scalvini,[1] Francesca Galvani,[3] Riccardo Castelli,[1] Lorenzo Sarcone,[1] Adriano Recchia,[2] Fabiola Fanini,[2,#] Adren Tran,[3] Silvia Rivara,[1,*] Gilberto Spadoni,[2] Daniele Piomelli,[3,^] Marco Mor[1,^]

[1]Dipartimento di Scienze degli Alimenti e del Farmaco, Università degli Studi di Parma, Parco Area delle Scienze 27/A, 43124, Parma, Italy
[2]Dipartimento di Scienze Biomolecolari, Università degli Studi di Urbino "Carlo Bo", Piazza Rinascimento 6, 61029, Urbino, Italy
[3]Department of Anatomy and Neurobiology, University of California, Irvine, California, USA.
[#]Current address: Department of Experimental Vascular Medicine, Amsterdam UMC, Location AMC, 1105 AZ Amsterdam, The Netherlands
^MM and DP share last authorship

Supplementary data

Table of contents:

1. Figures **Page S2**

2. ^{1}H NMR, ^{13}C NMR and HRMS spectra of target compounds **Page S3**

3. HPLC purity of target compounds **Page S32**

1. Figures

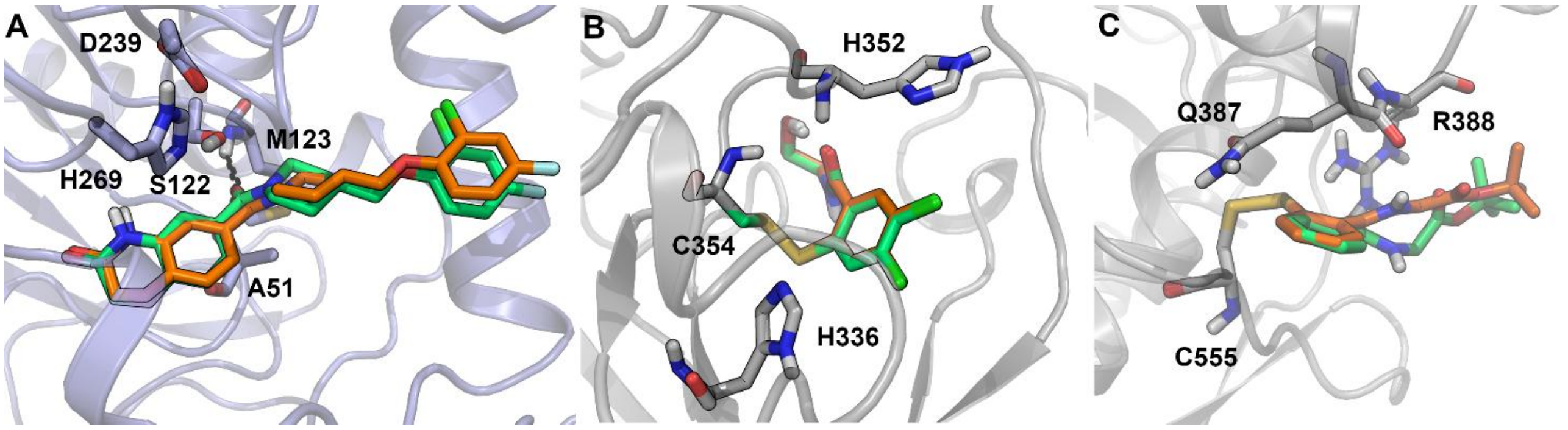

Figure S1. Superposition of the crystallographic ligand pose (green carbon atoms) with the corresponding top-ranked docking pose (orange carbon atoms). Panel A: non-covalent re-docking of a benzoxazinone inhibitor into human MGL (PDB ID: 3HJU). Panel B: covalent docking of a benzisothiazolinone derivative into microbial urease (PDB ID: 9REY). Panel C: covalent docking of a benzisothiazolinone derivative into *Mycobacterium tuberculosis* transpeptidase LdtMt2 (PDB ID: 8A1K). Protein residues are shown as grey sticks, and the protein secondary structure is represented as a grey cartoon.

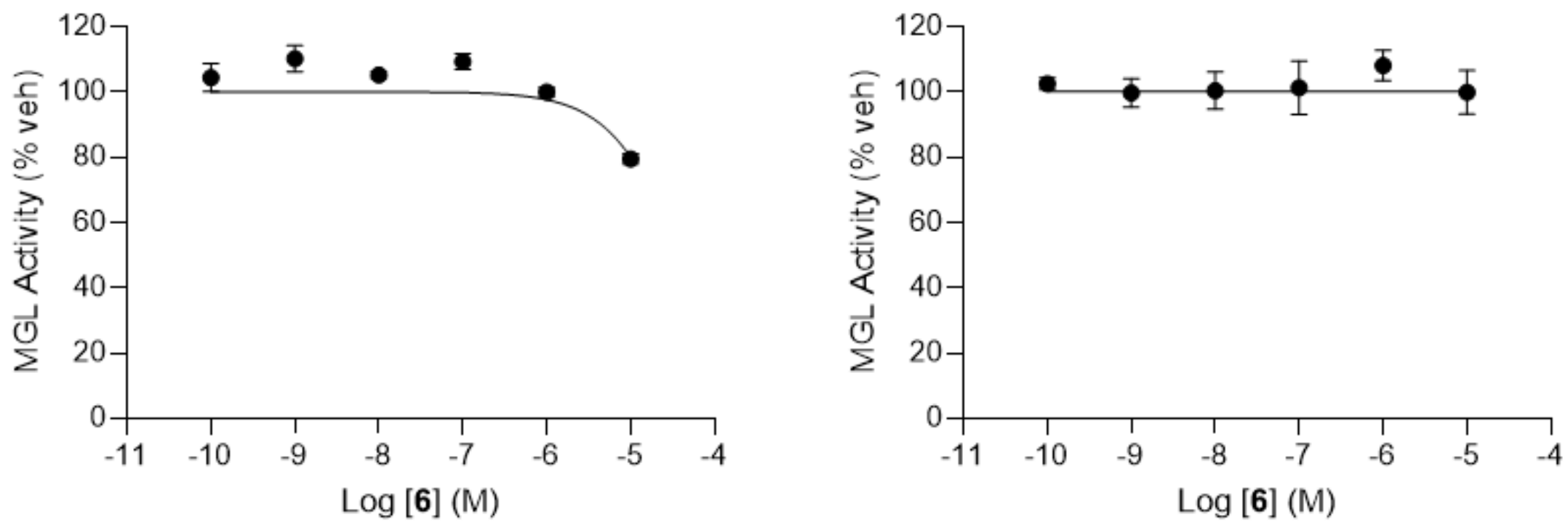

Figure S2. Effect of compound **6** on activity of human monoacylglycerol lipase.

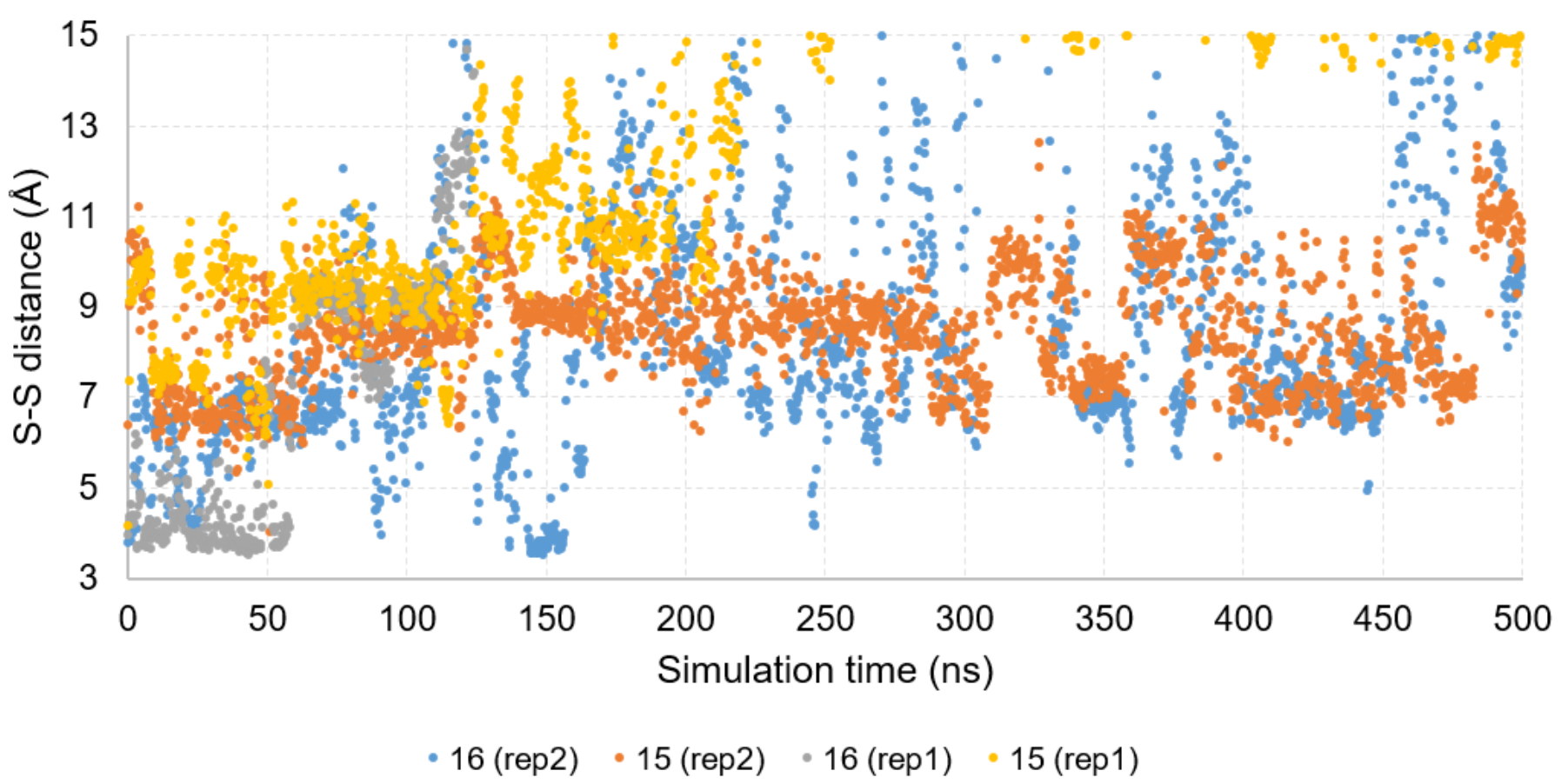

Figure S3. Time evolution of the S–S distance between the sulfur atom of Cys201 and the sulfur atom of the benzisothiazolinone moiety during two independent replicas (rep1 and rep2) of 500 ns molecular dynamics simulations of the non-covalent complexes of MGL with **15** (orange and yellow) or **16** (blue and gray). Distances were averaged over consecutive 250 ps intervals.

2. ^{1}H NMR, ^{13}C NMR and HRMS spectra of target compounds

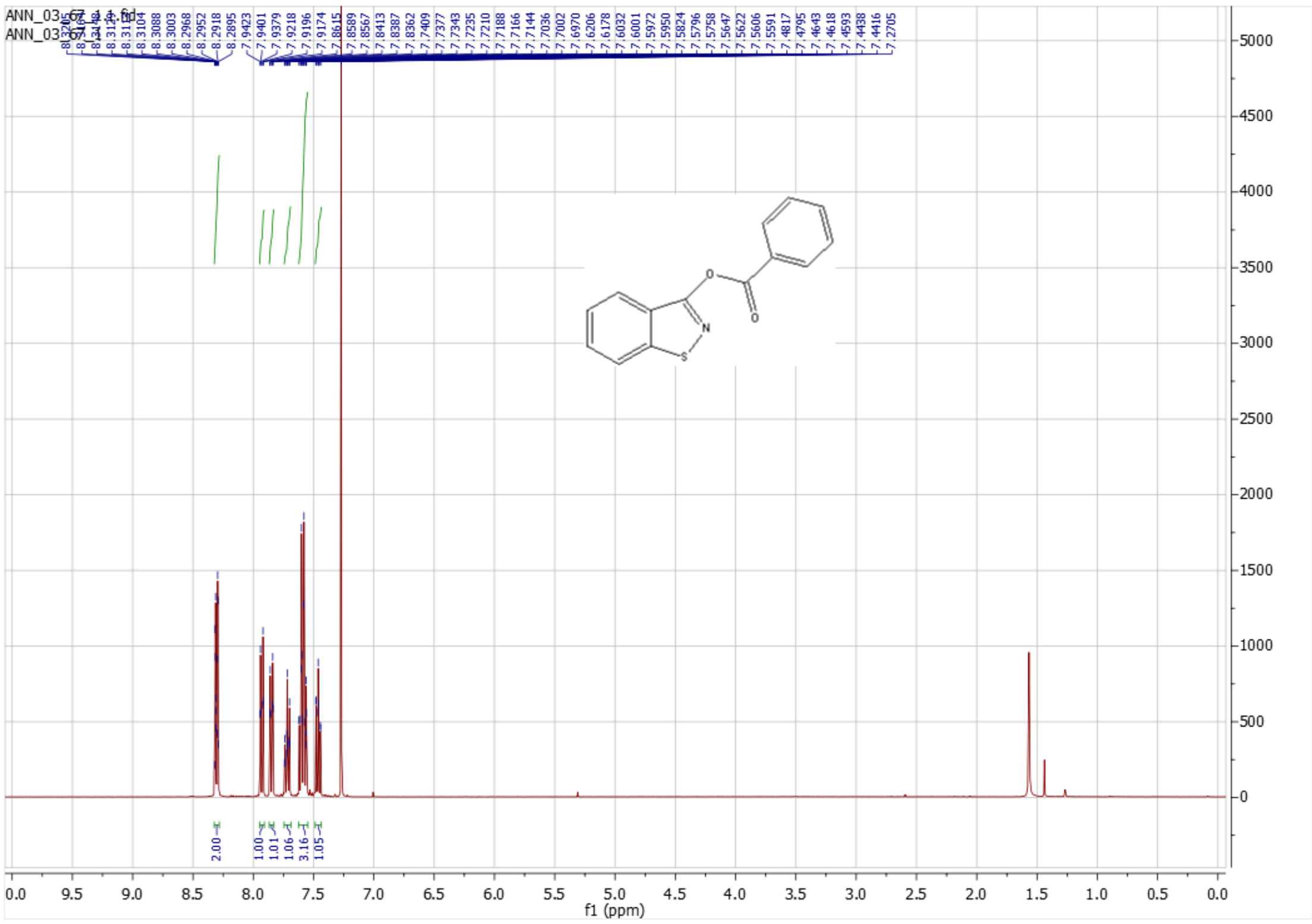

^{1}H NMR spectrum (400 MHz, $CDCl_3$) of compound **1a**.

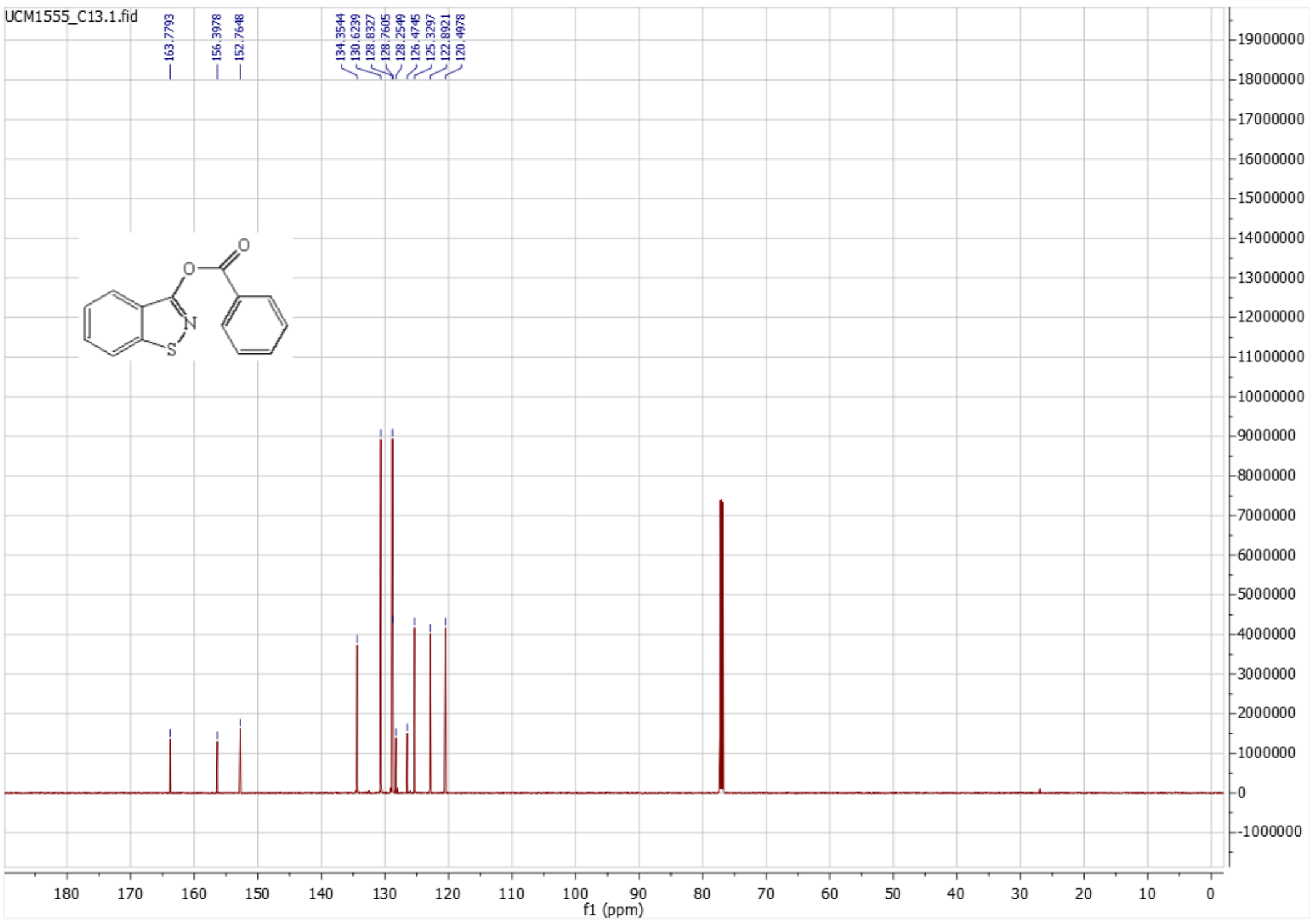

^{13}C NMR spectrum (150 MHz, $CDCl_3$) of compound **1a**.

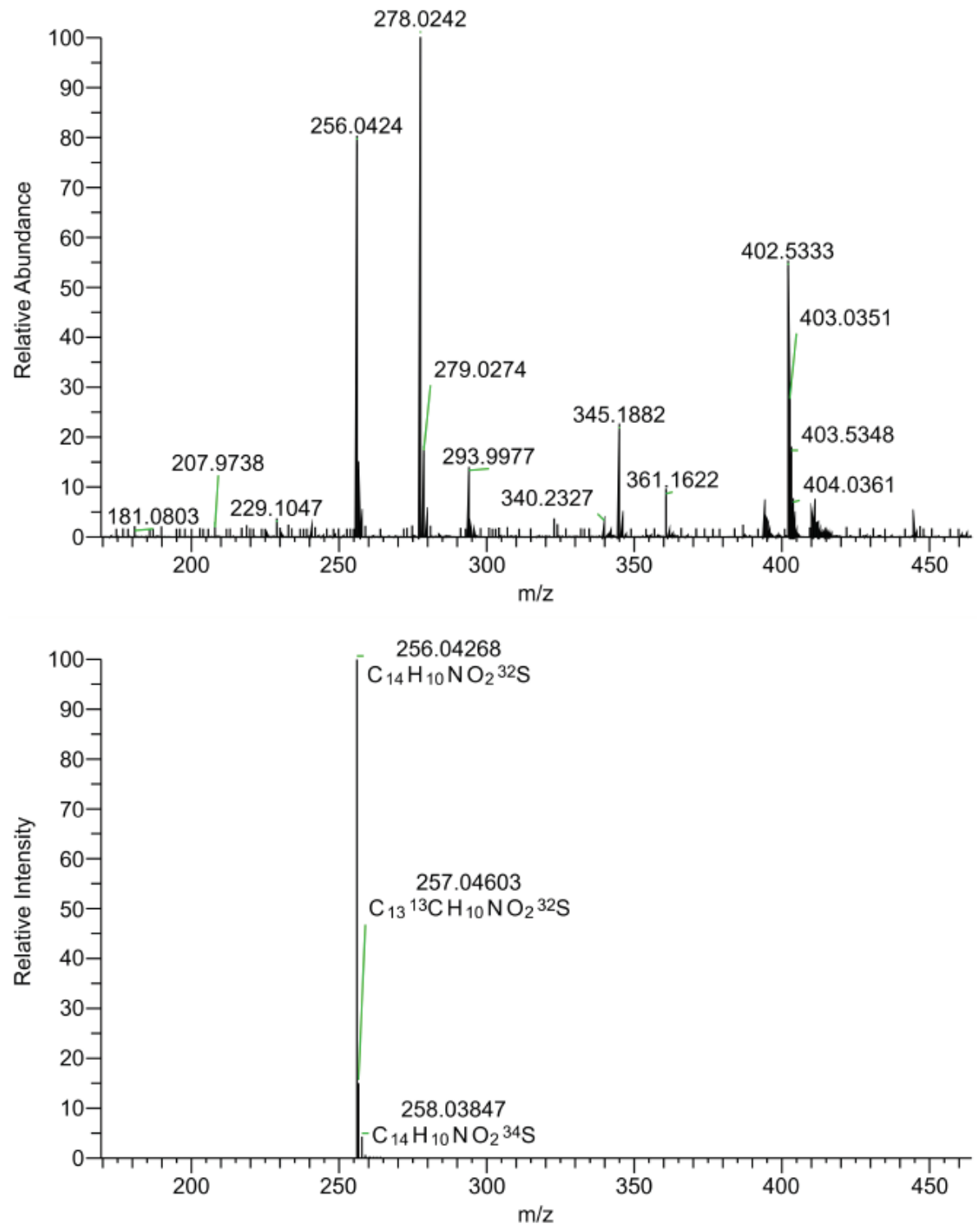

HRMS of compound **1a**.

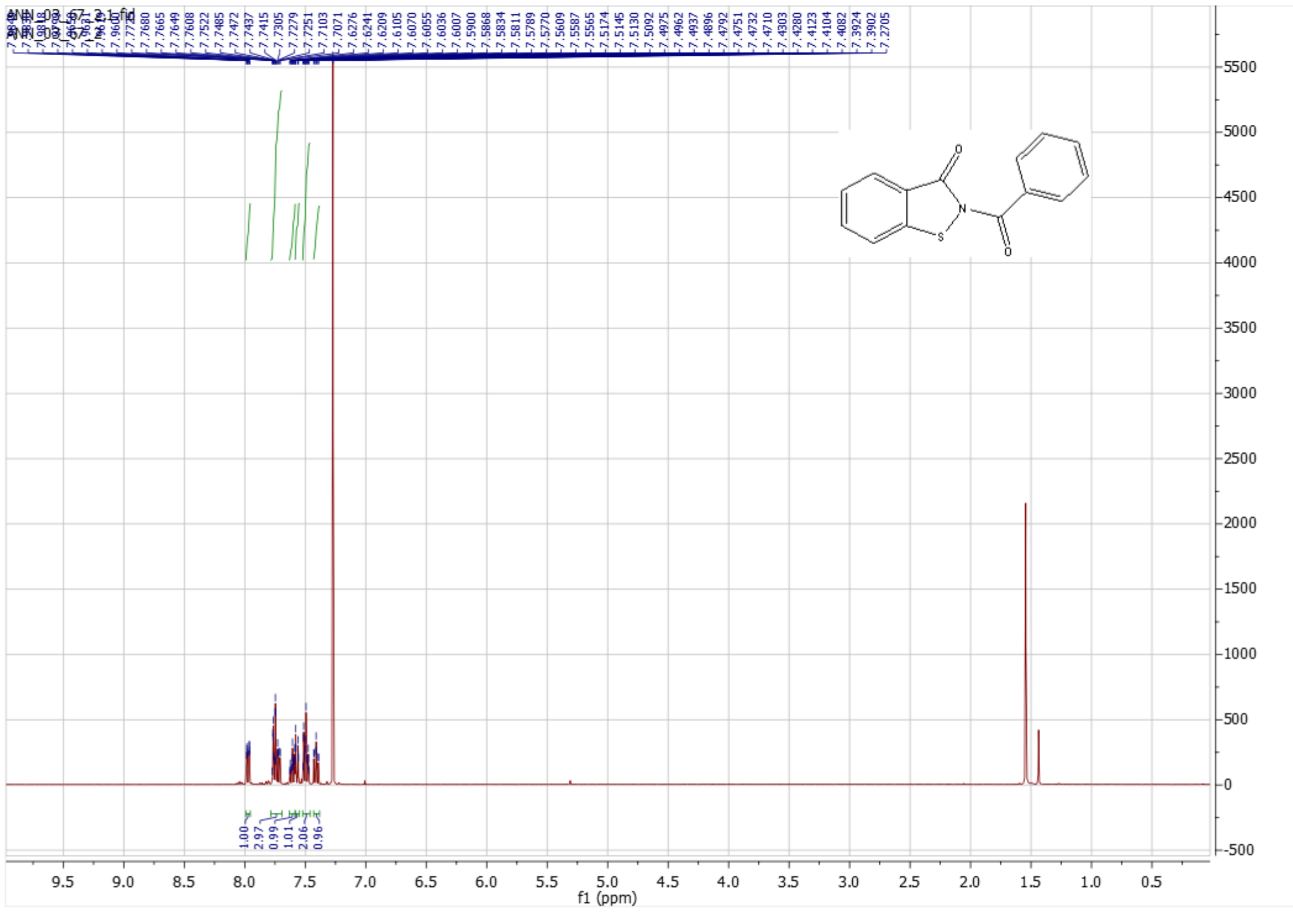

^{1}H NMR spectrum (400 MHz, $CDCl_3$) of compound **2a**.

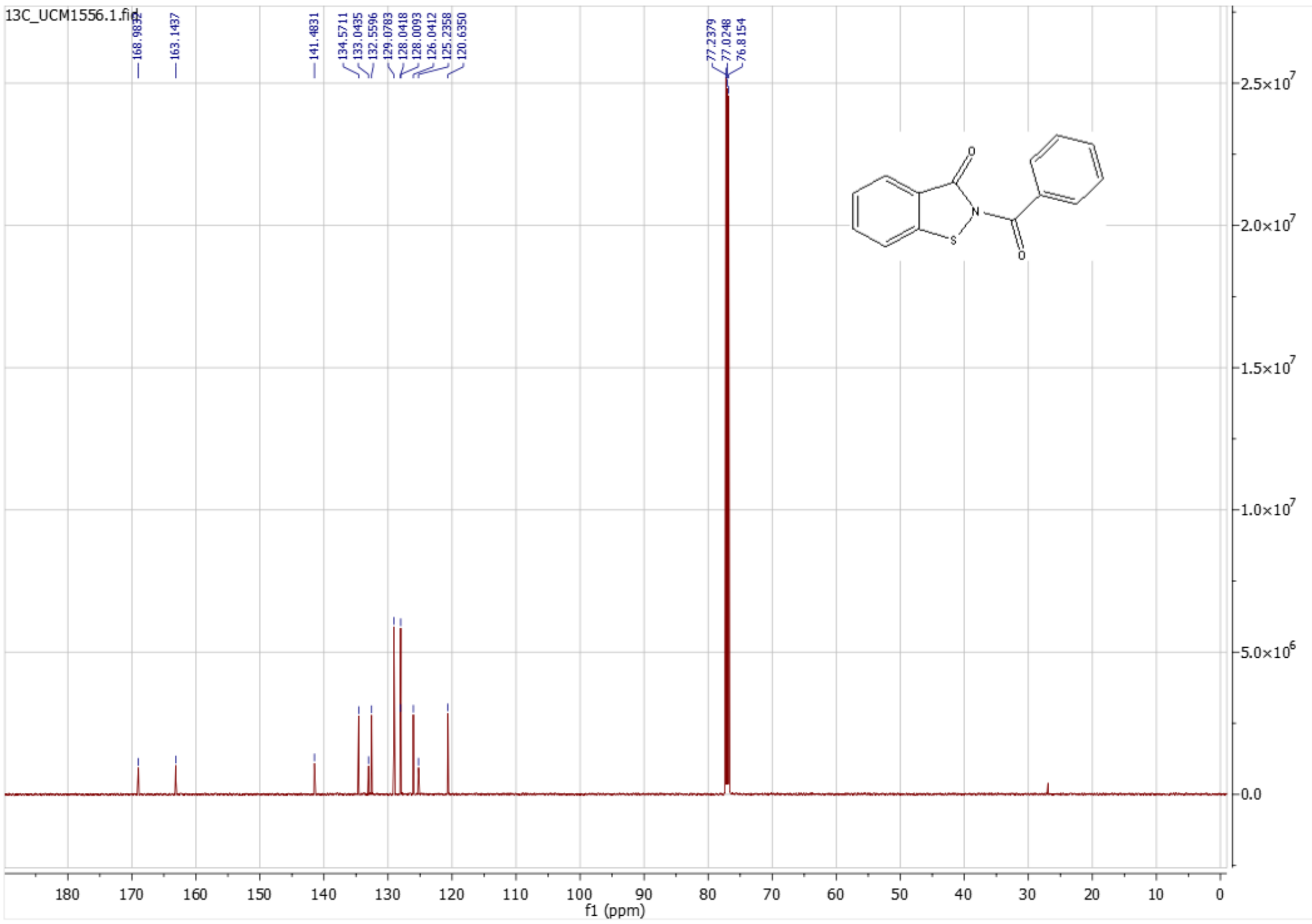

^{13}C NMR spectrum (150 MHz, $CDCl_3$) of compound **2a**.

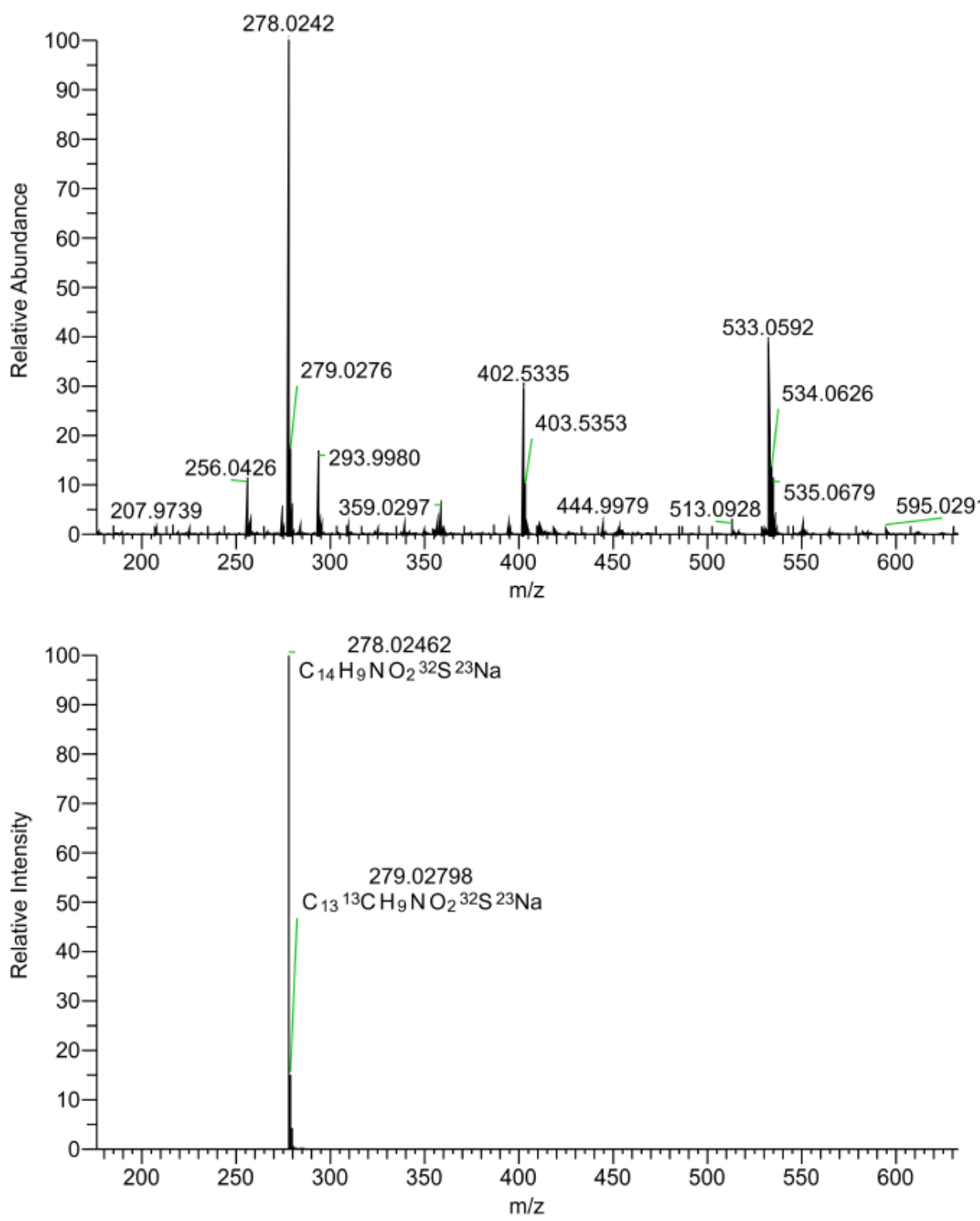

HRMS of compound **2a**.

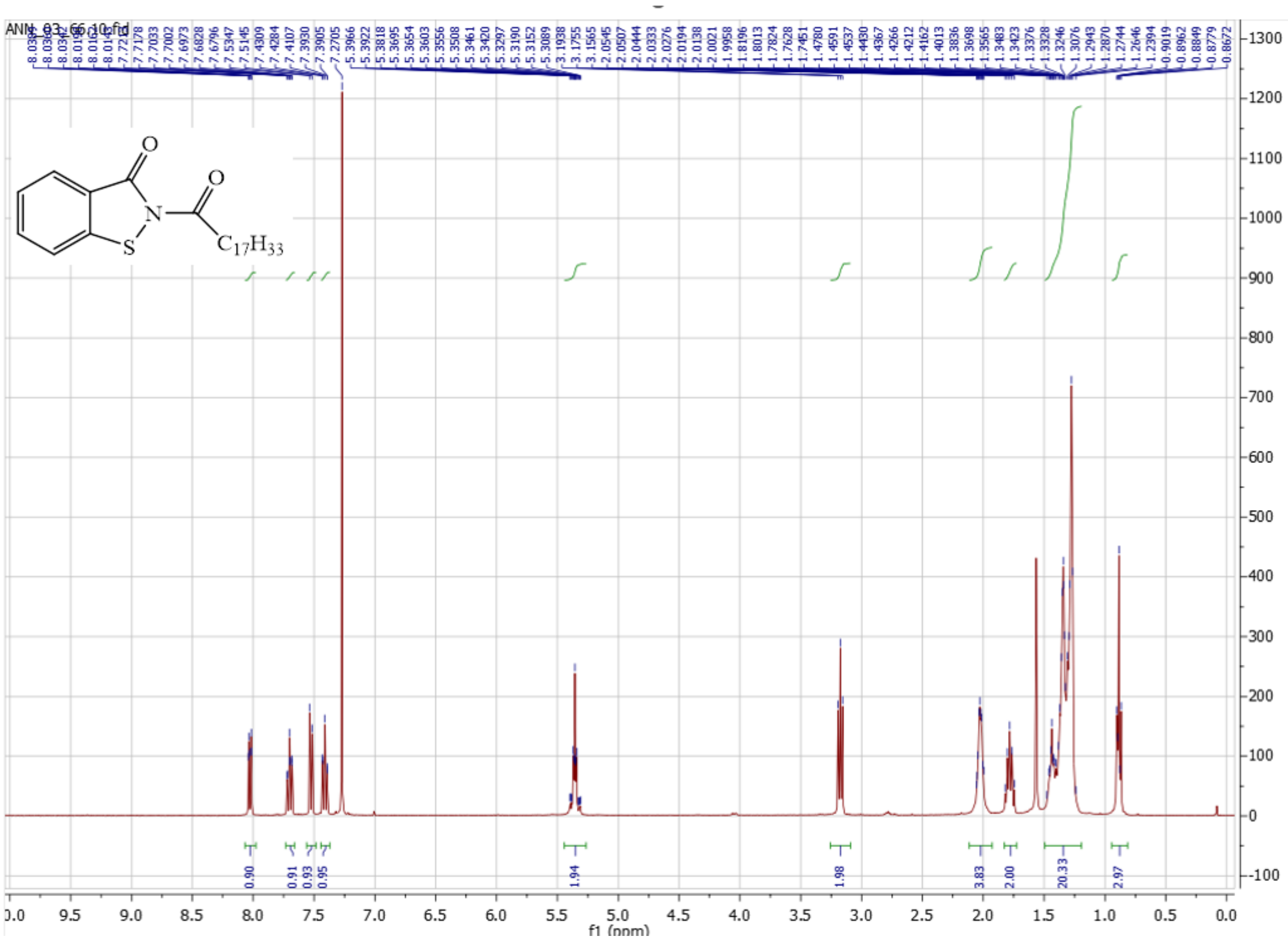

^{1}H NMR spectrum (400 MHz, $CDCl_3$) of compound **2b**.

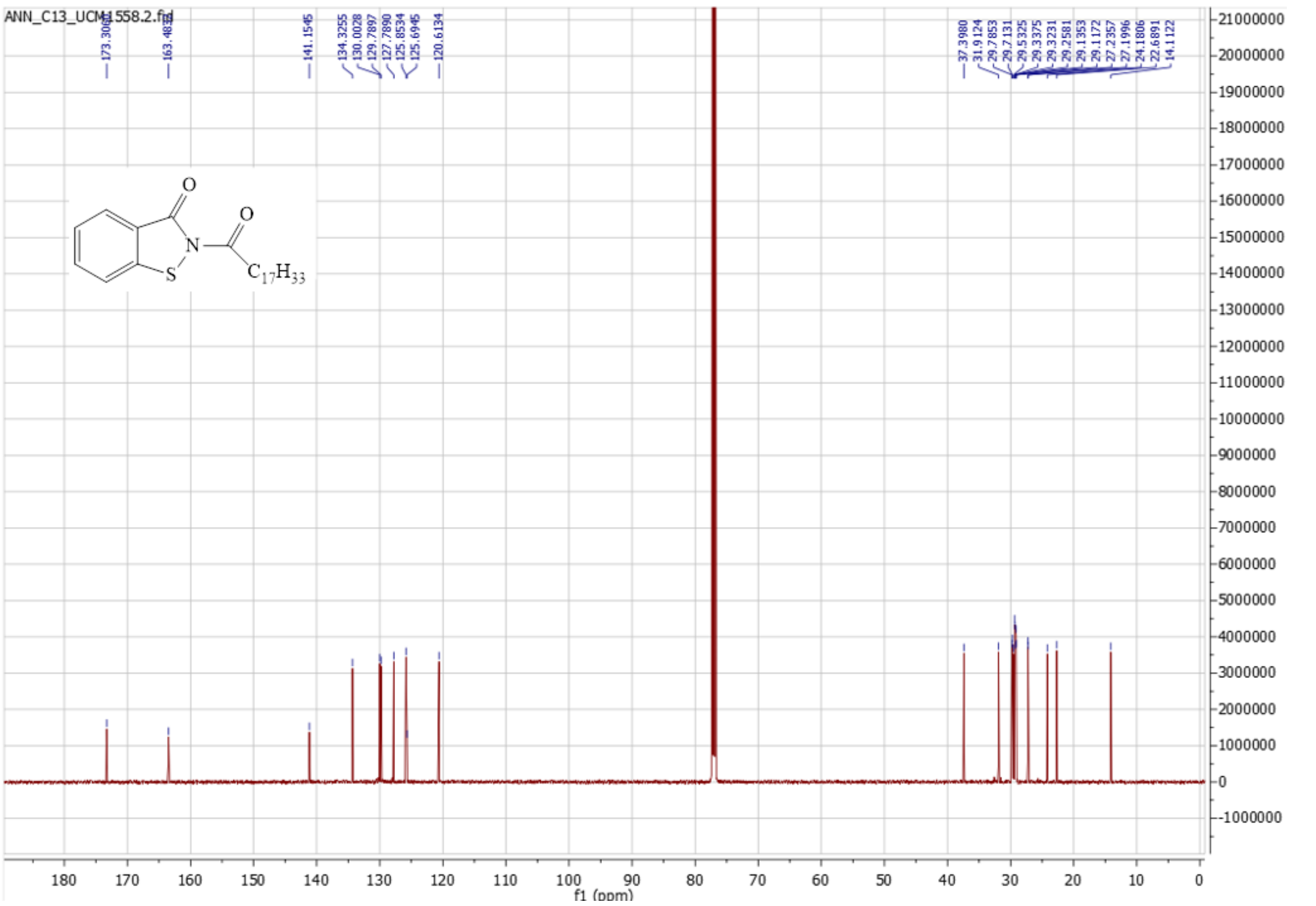

^{13}C NMR spectrum (150 MHz, $CDCl_3$) of compound **2b**.

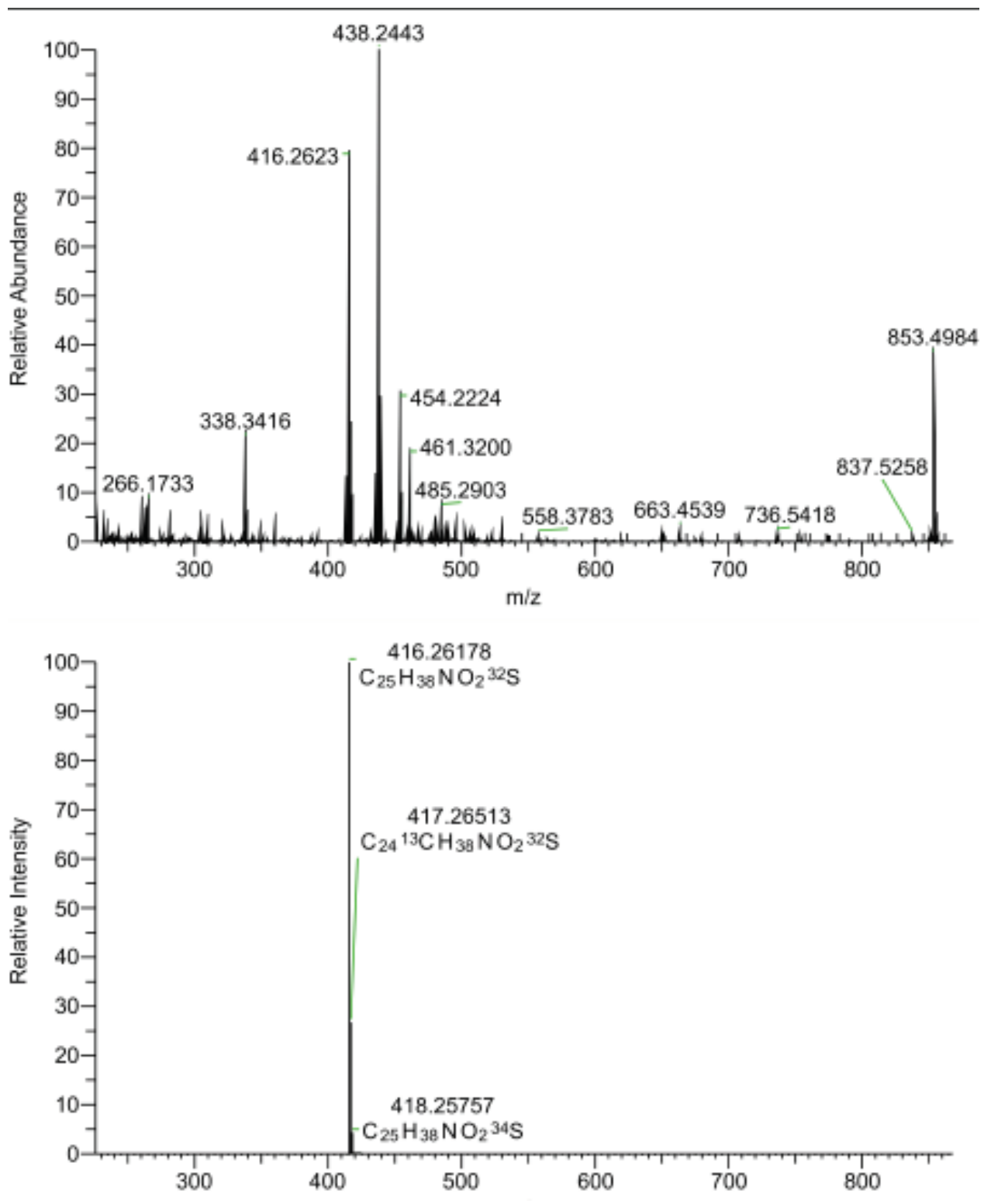

HRMS of compound **2b**.

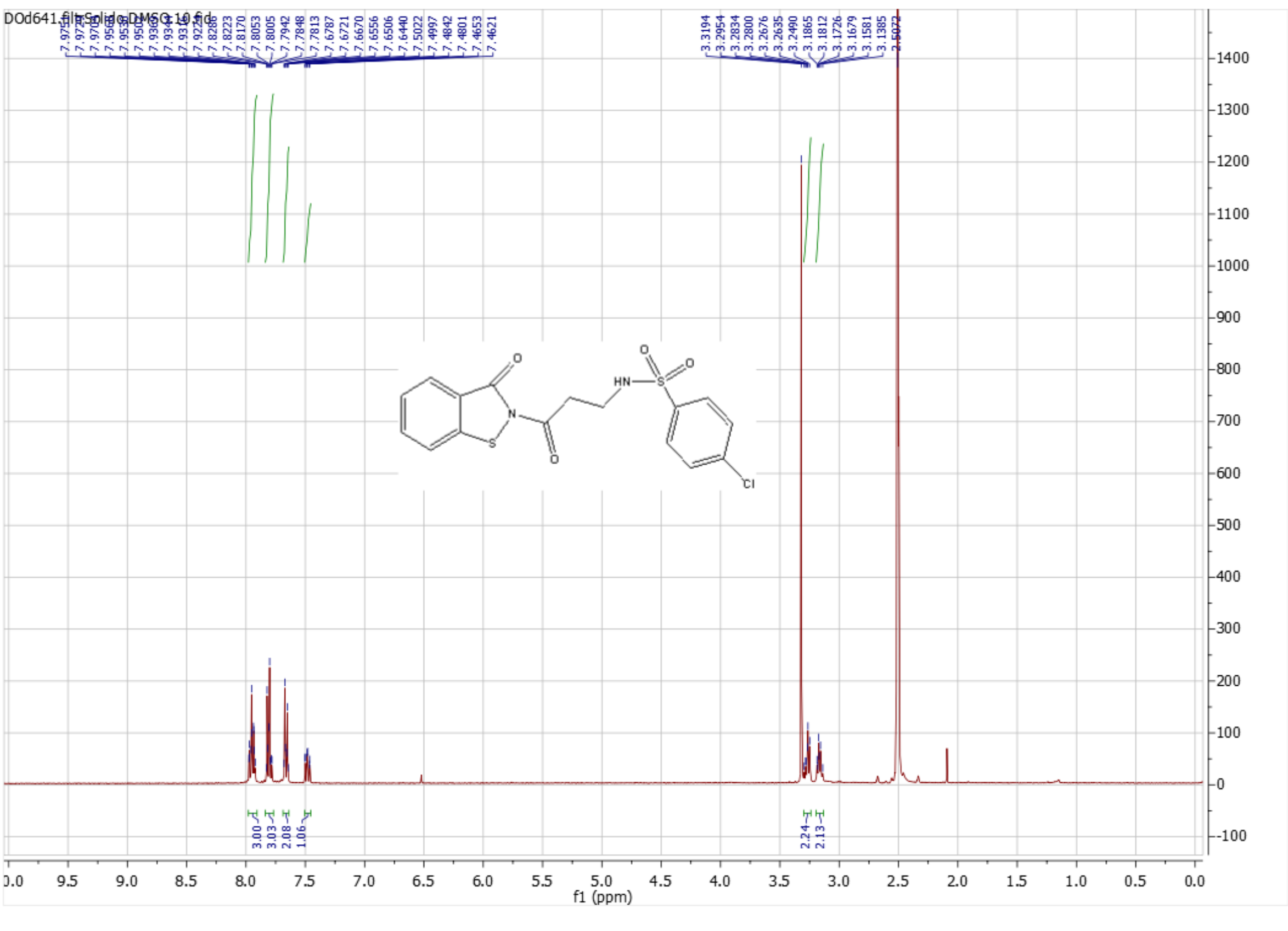

^{1}H NMR spectrum (400 MHz, DMSO-d_6) of compound **2c**.

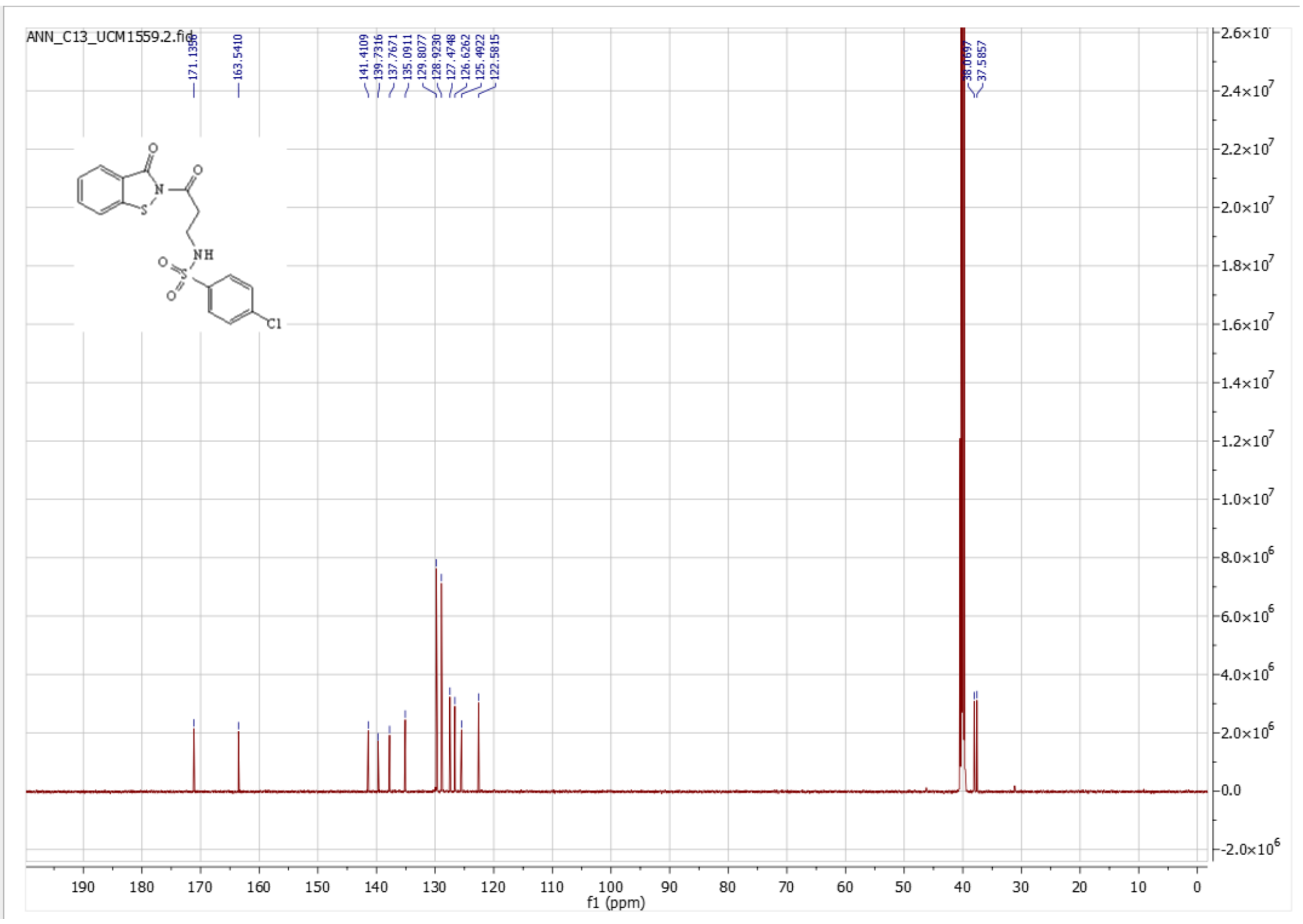

^{13}C NMR spectrum (150 MHz, DMSO-d_6) of compound **2c**.

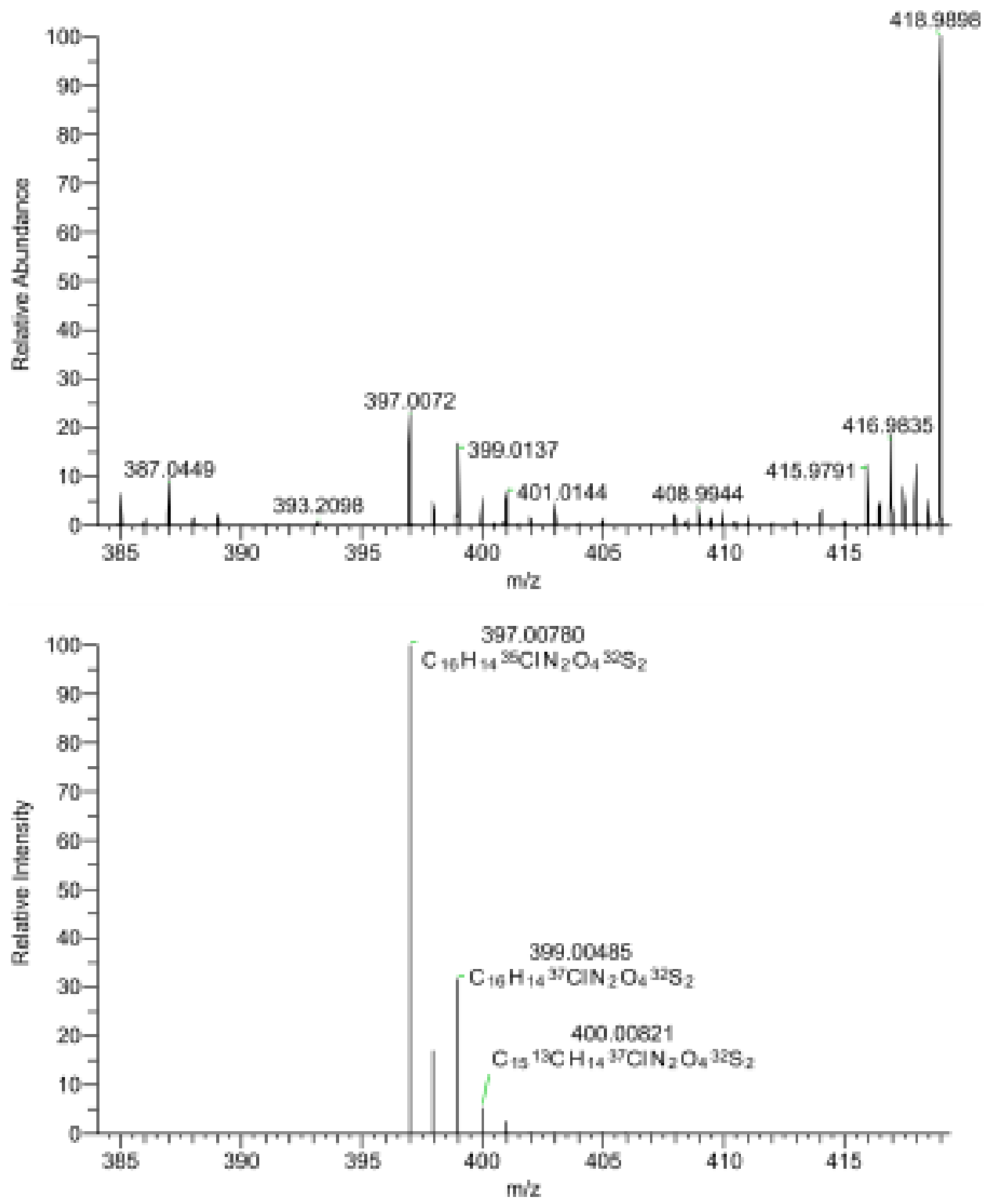

HRMS of compound **2c**.

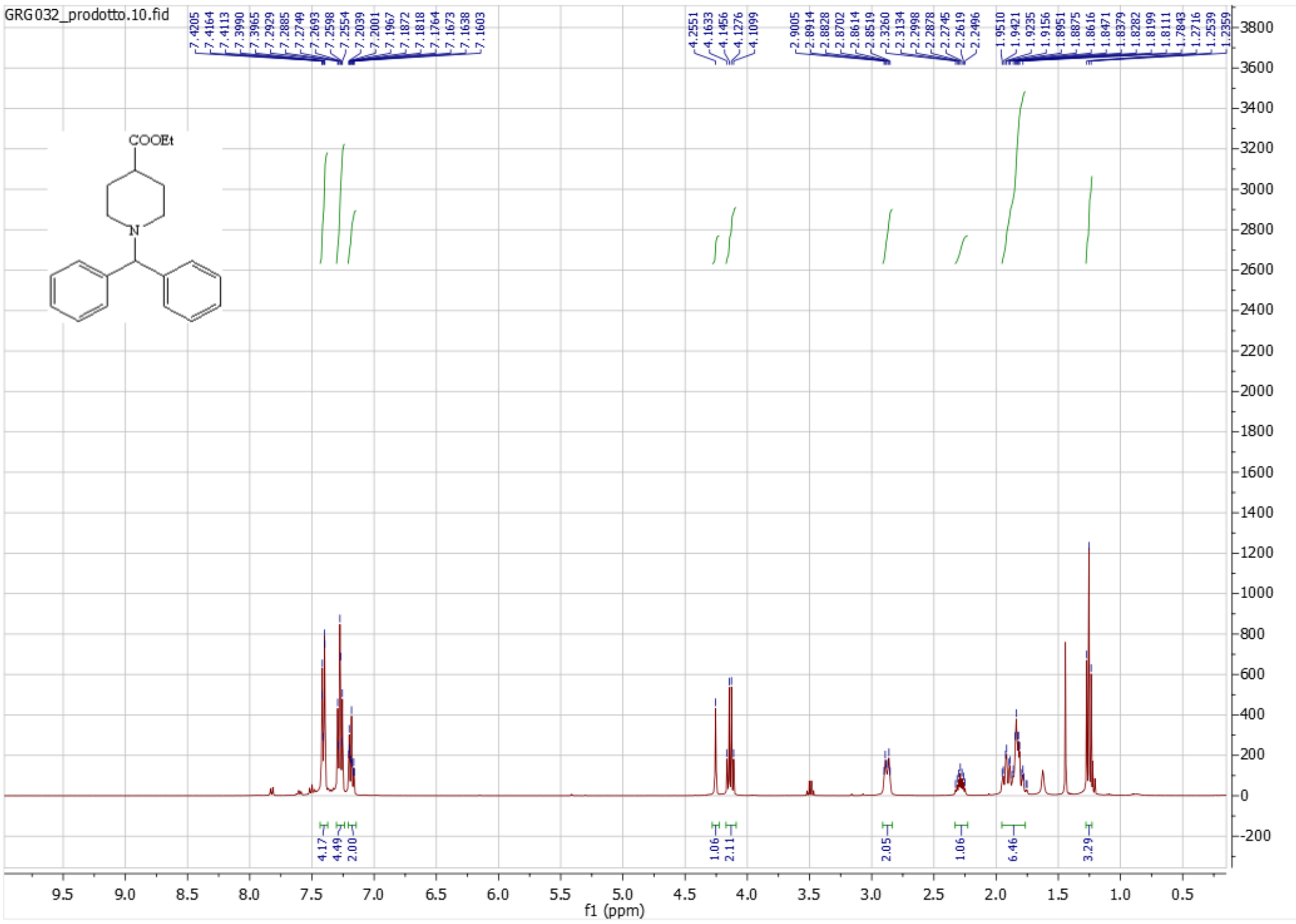

^{1}H NMR spectrum (400 MHz, $CDCl_3$) of compound **3**.

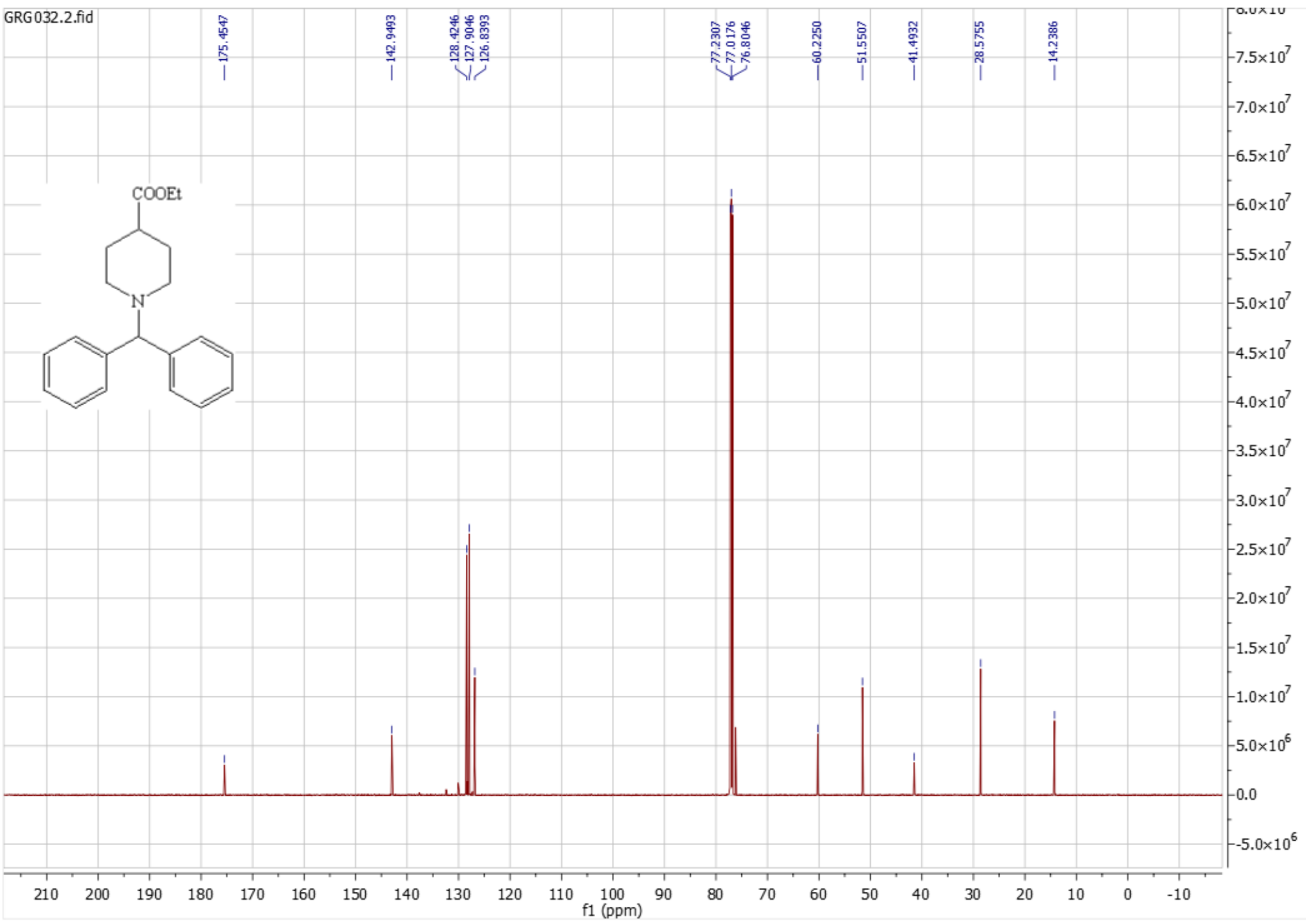

^{13}C NMR spectrum (150 MHz, $CDCl_3$) of compound **3**.

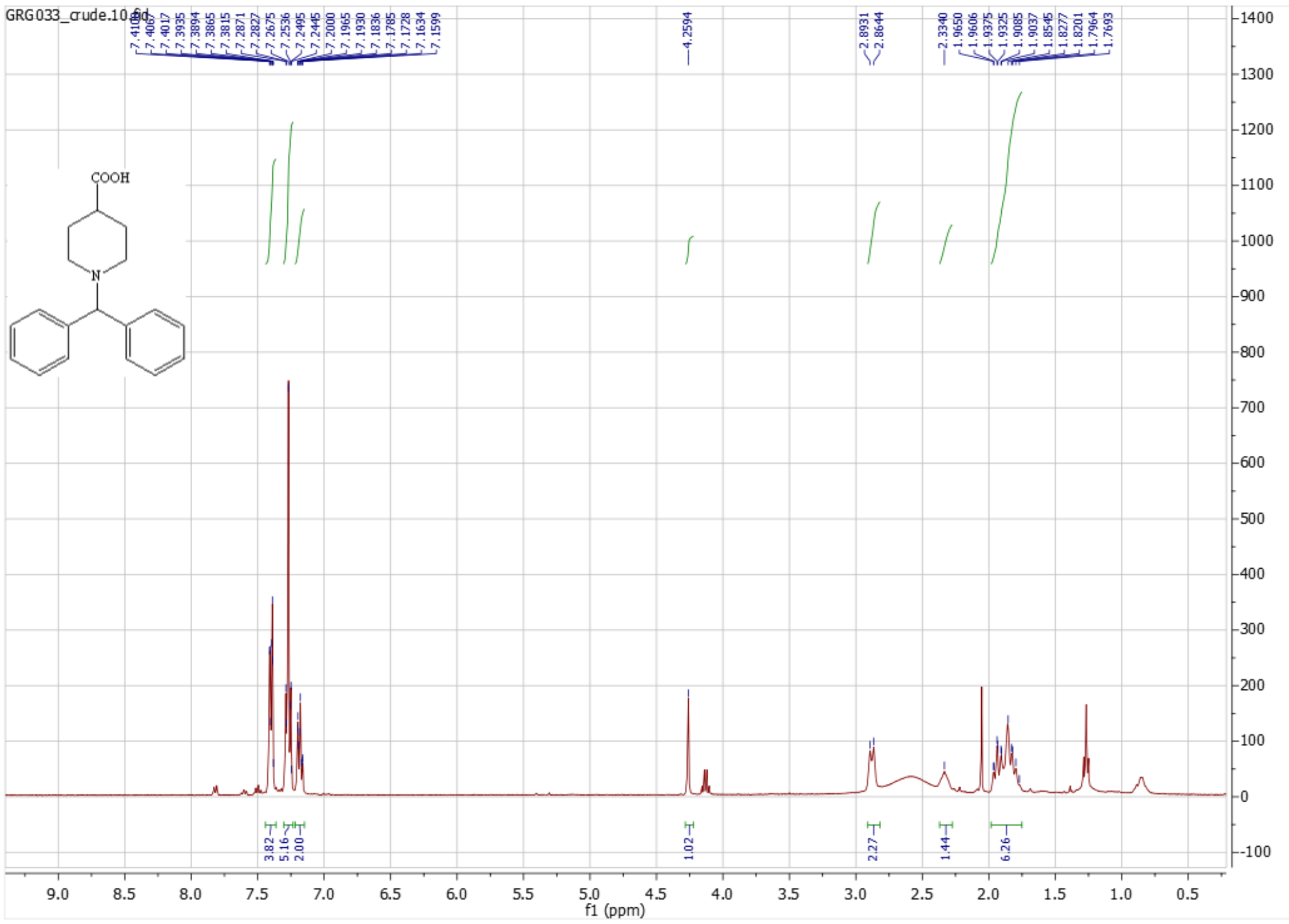

^{1}H NMR spectrum (400 MHz, $CDCl_3$) of compound **4**.

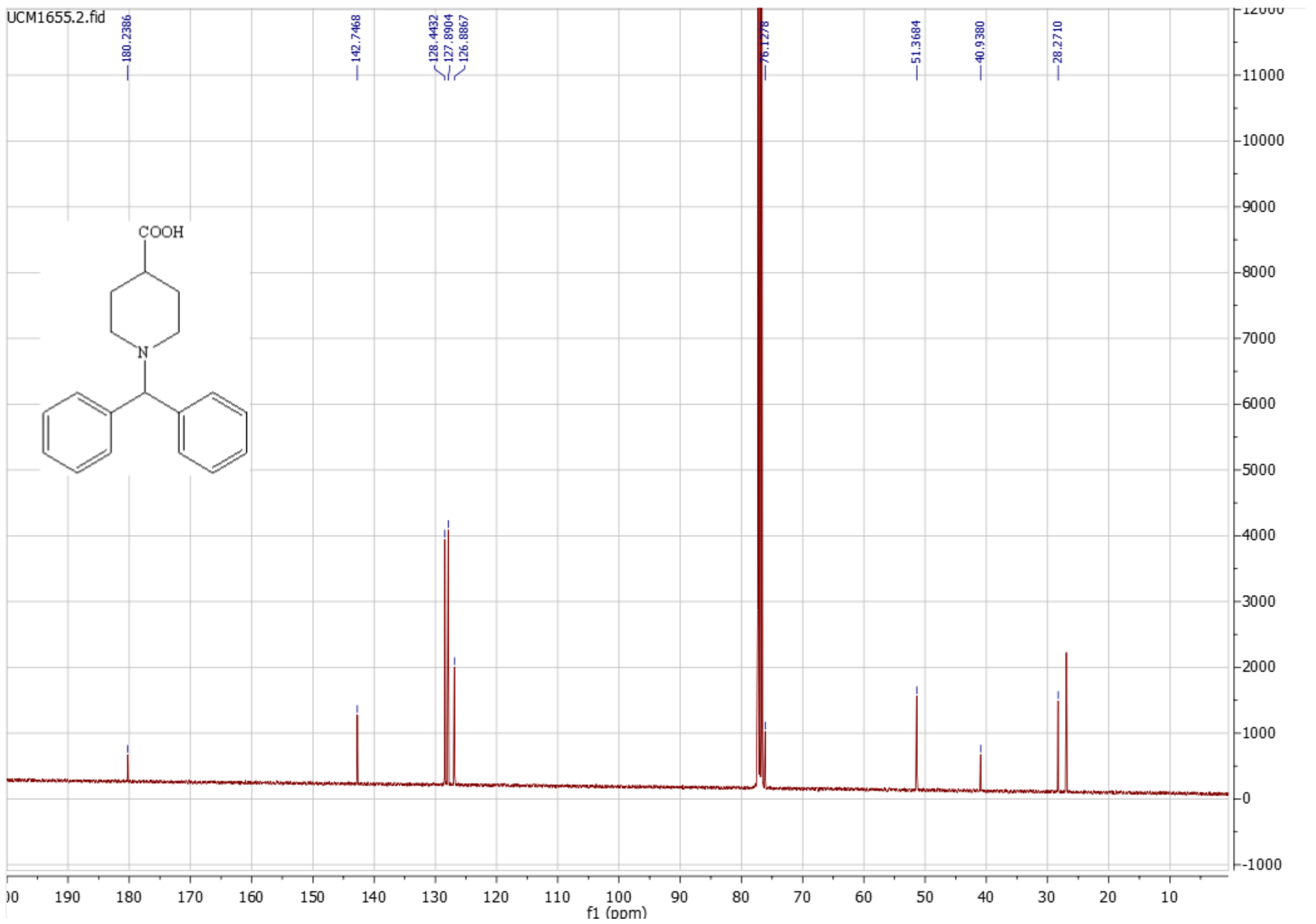

^{13}C NMR spectrum (100 MHz, $CDCl_3$) of compound **4**.

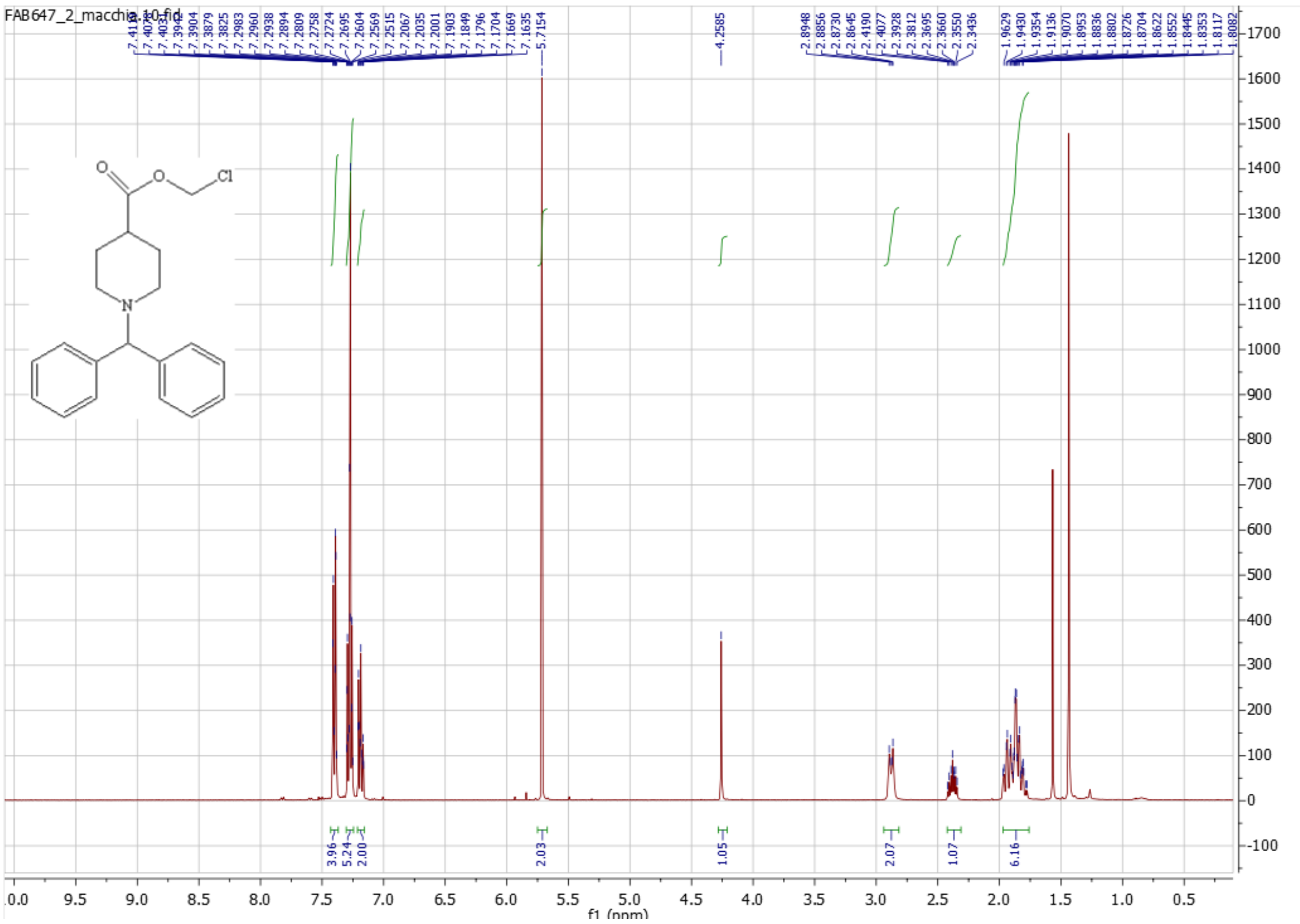

^{1}H NMR spectrum (400 MHz, $CDCl_3$) of compound **5**.

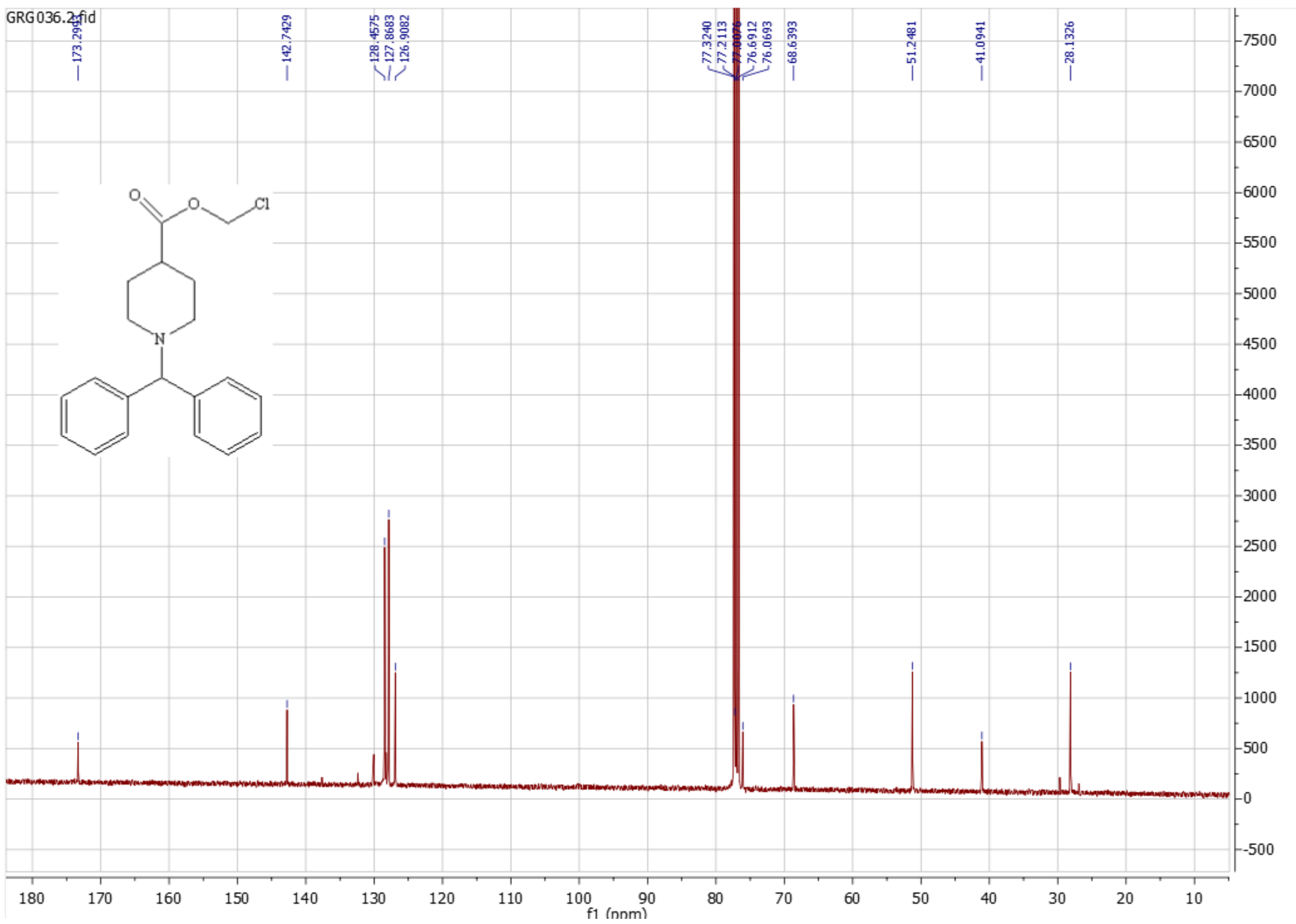

^{13}C NMR spectrum (100 MHz, $CDCl_3$) of compound **5**.

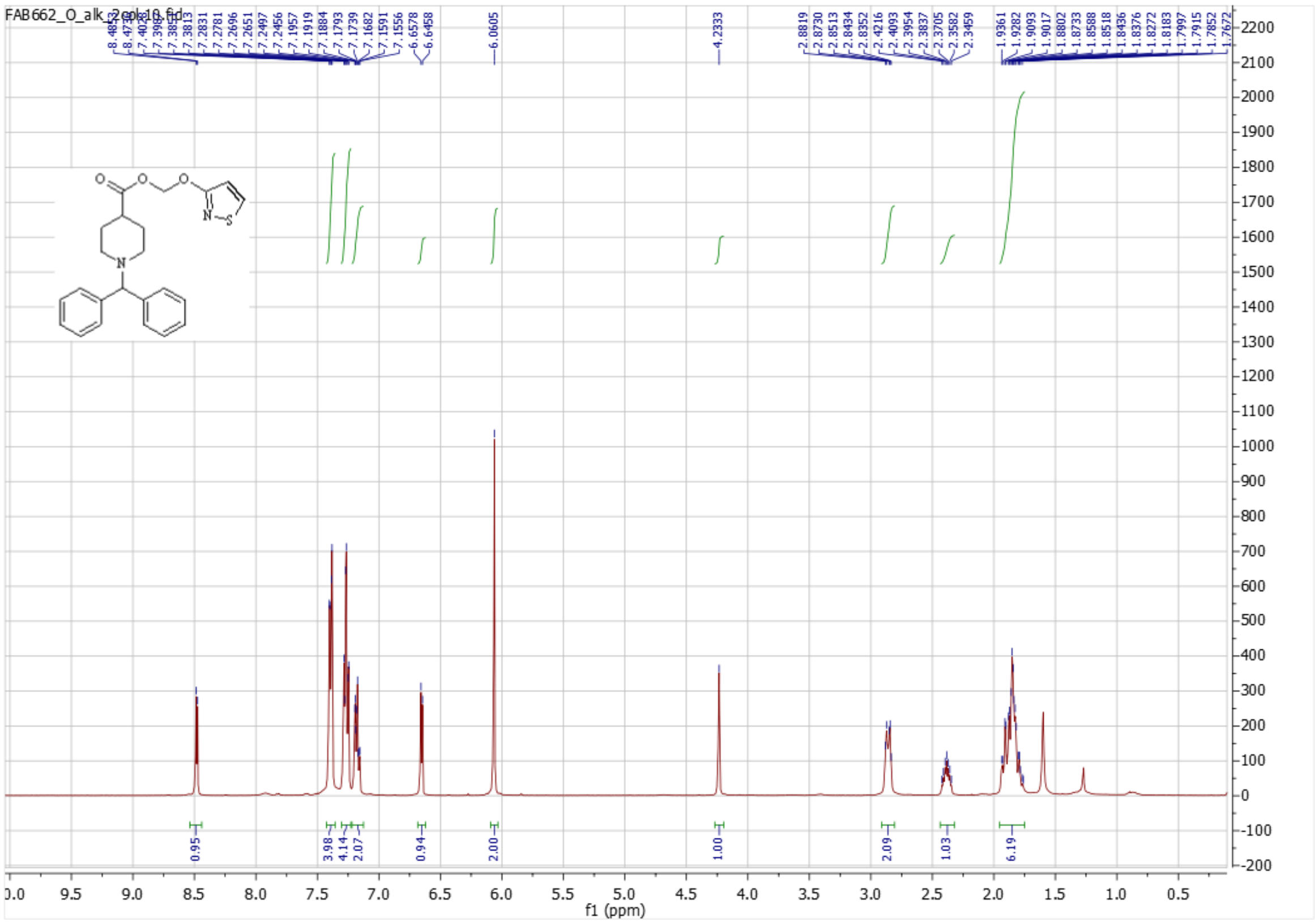

^{1}H NMR spectrum (400 MHz, $CDCl_3$) of compound **6**.

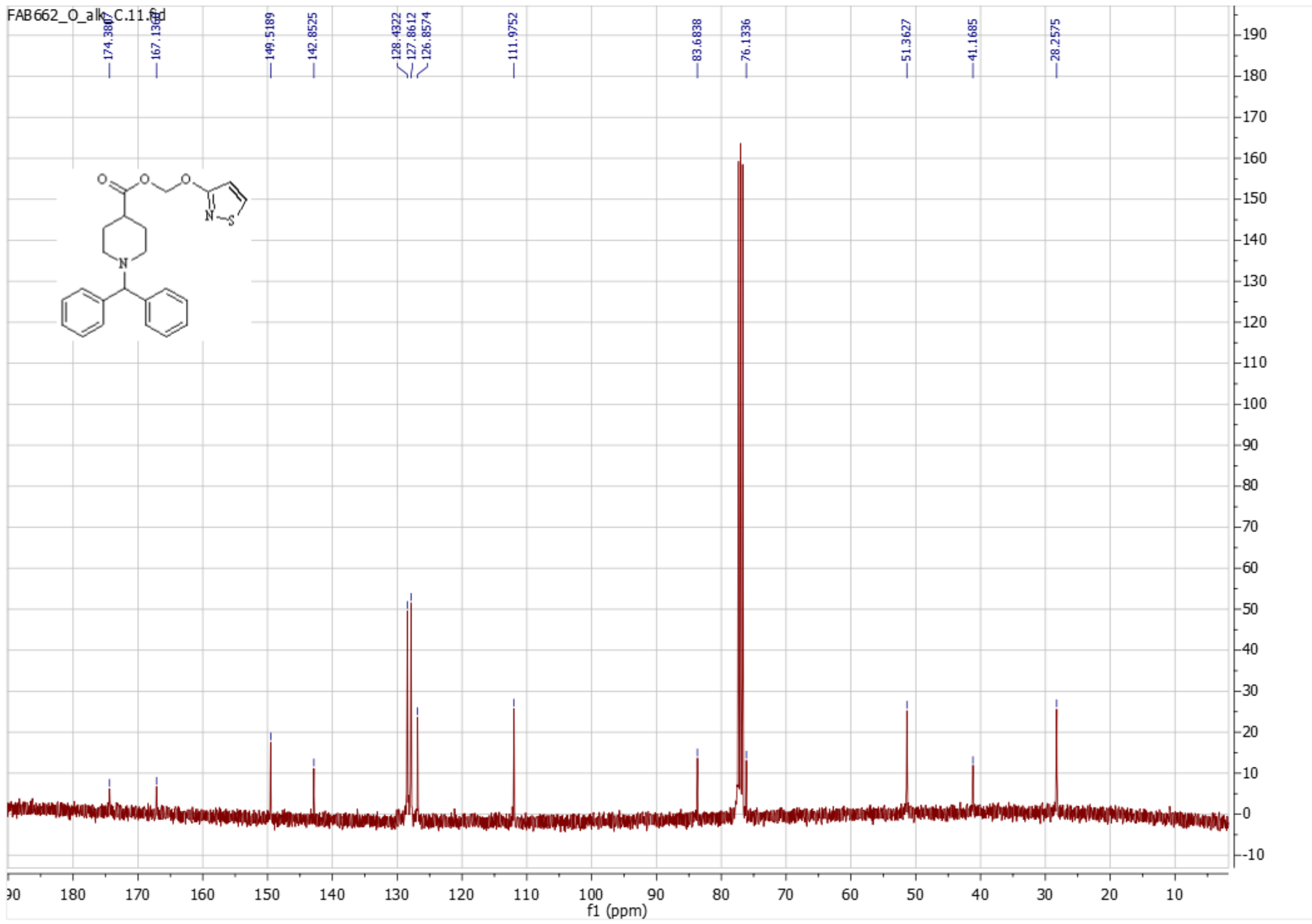

^{13}C NMR spectrum (100 MHz, $CDCl_3$) of compound **6**.

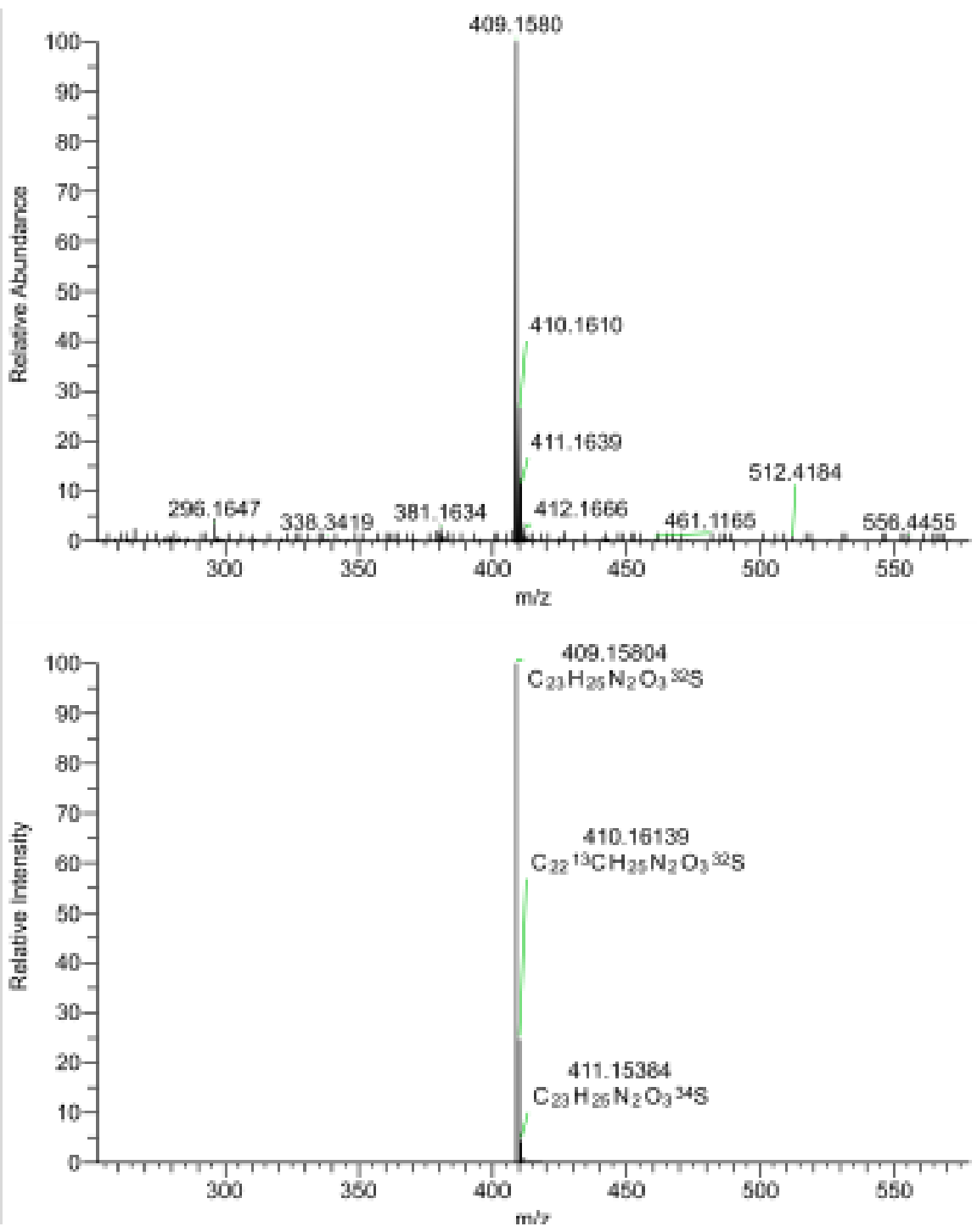

HRMS of compound **6**.

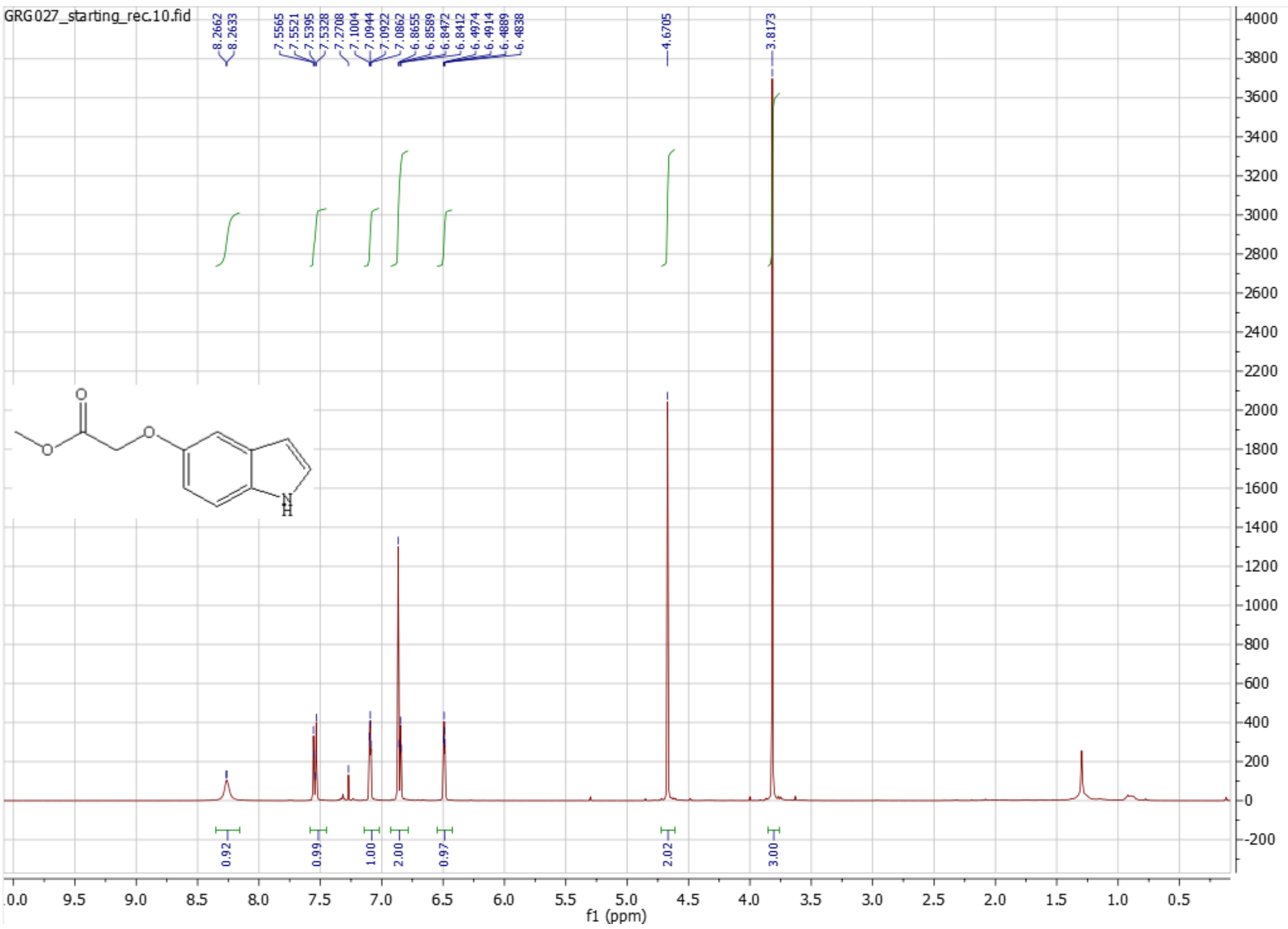

^{1}H NMR spectrum (400 MHz, $CDCl_3$) of compound **7**.

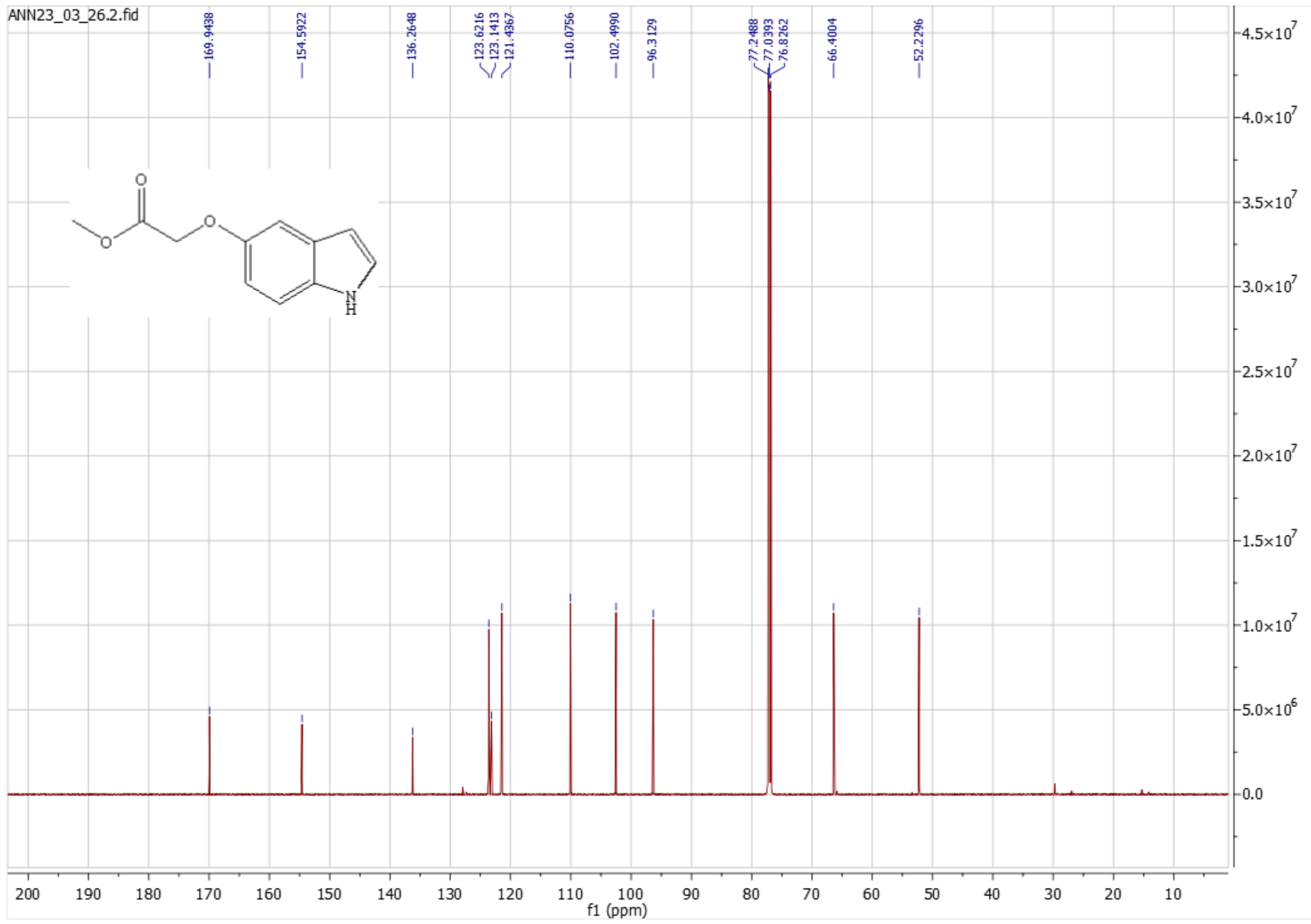

^{13}C NMR spectrum (150 MHz, $CDCl_3$) of compound **7**.

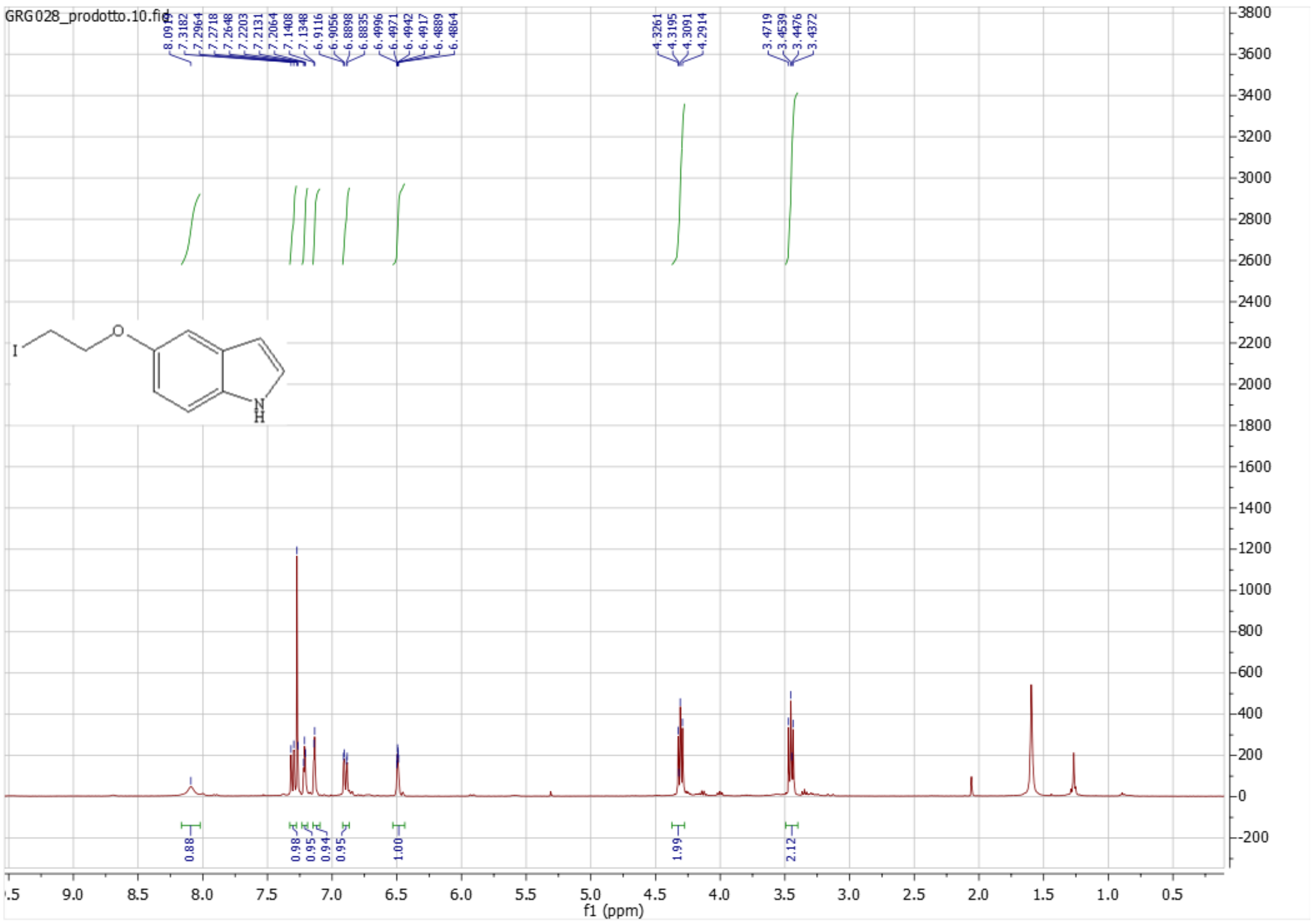

^{1}H NMR spectrum (400 MHz, $CDCl_3$) of compound **9**.

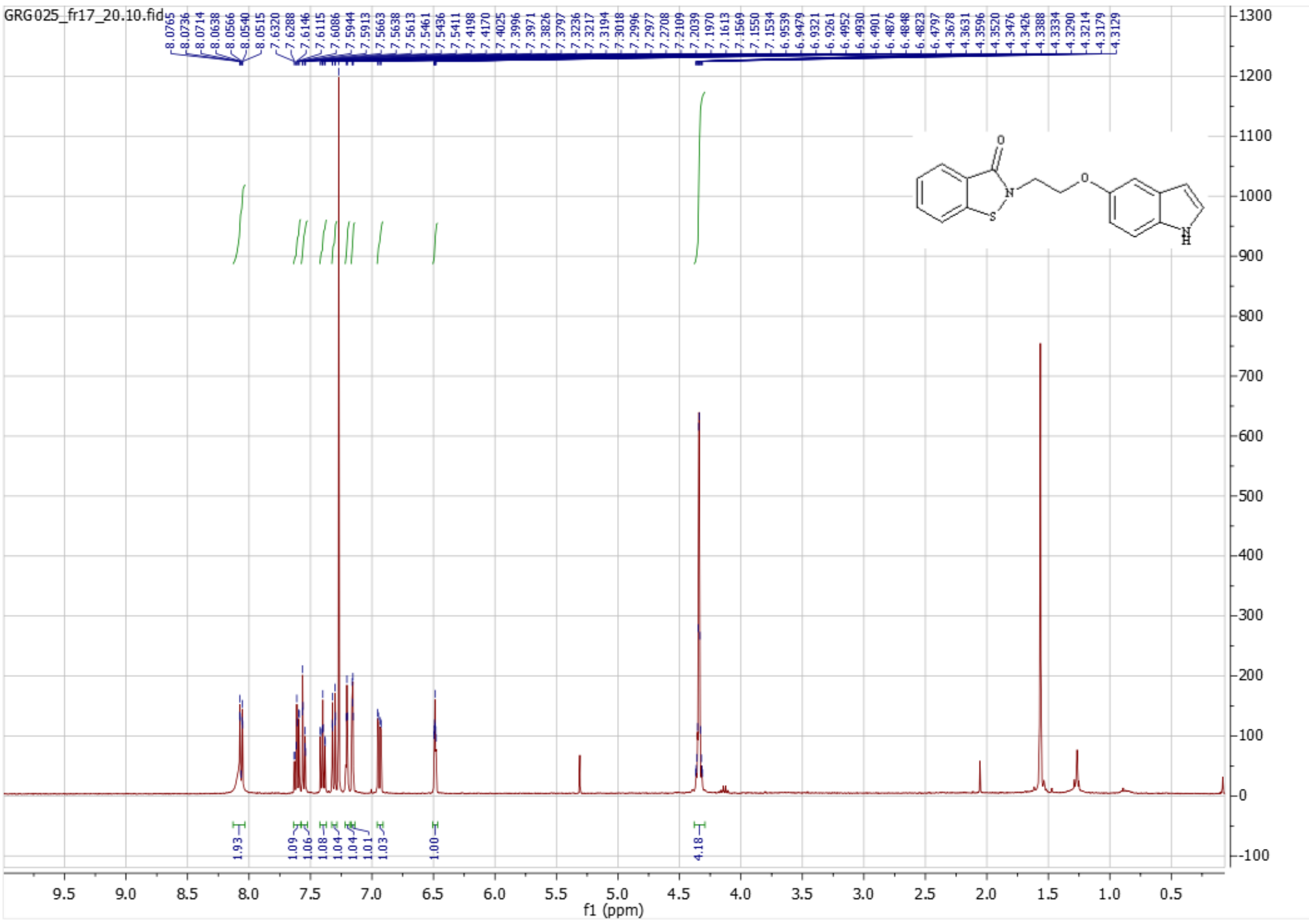

^{1}H NMR spectrum (400 MHz, $CDCl_3$) of compound **10**.

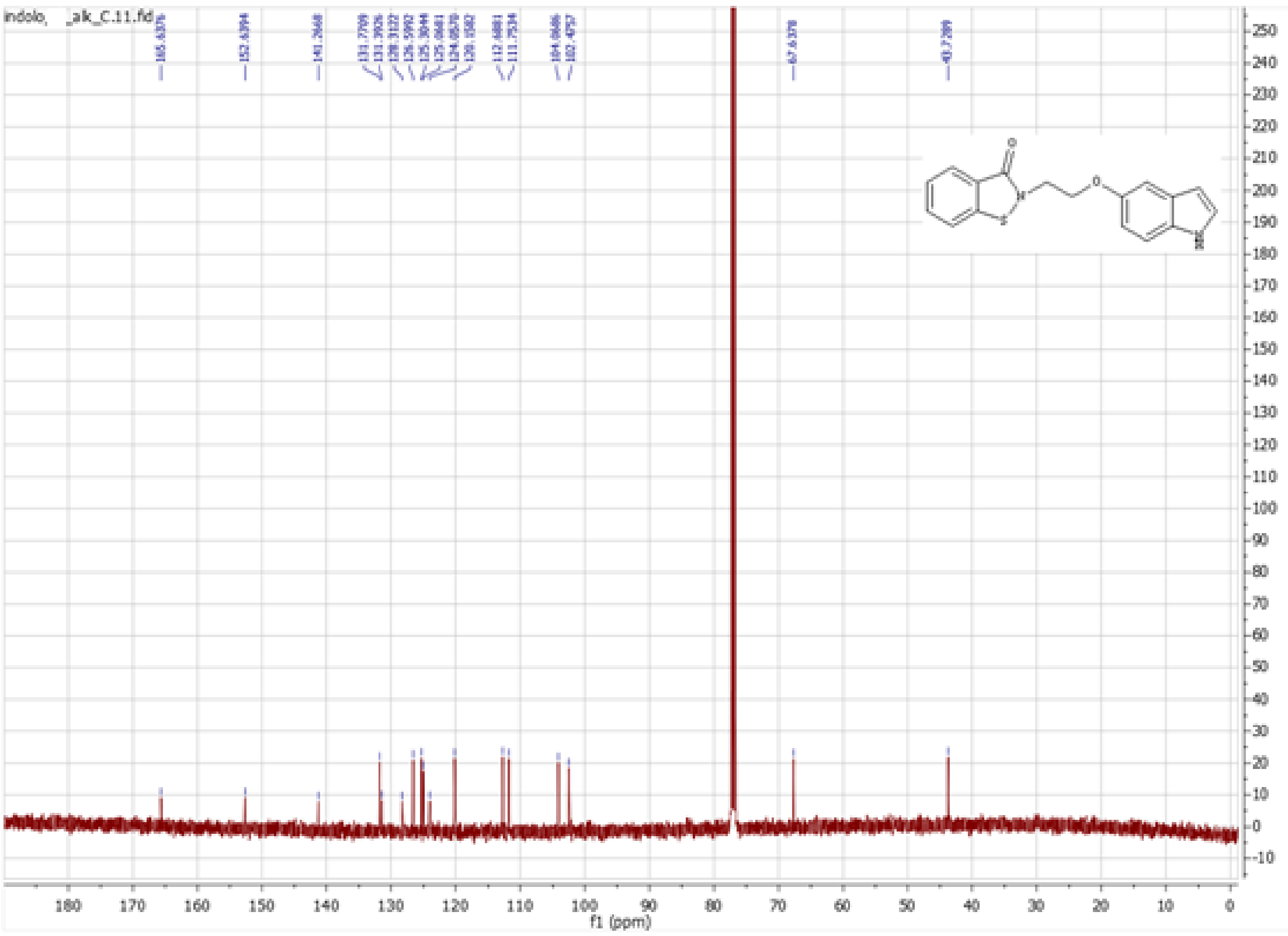

^{13}C NMR spectrum (100 MHz, $CDCl_3$) of compound **10**.

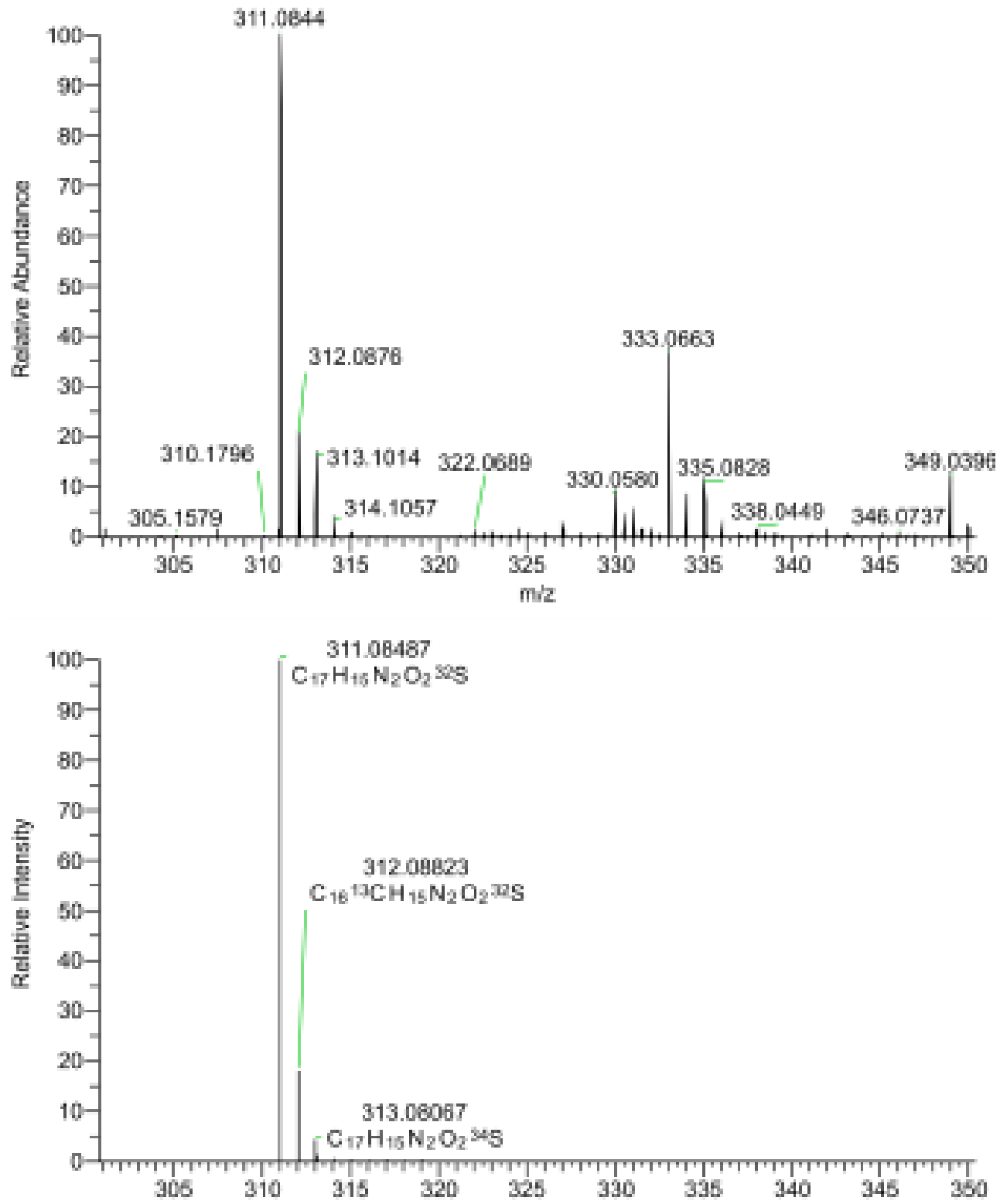

HRMS of compound **10**.

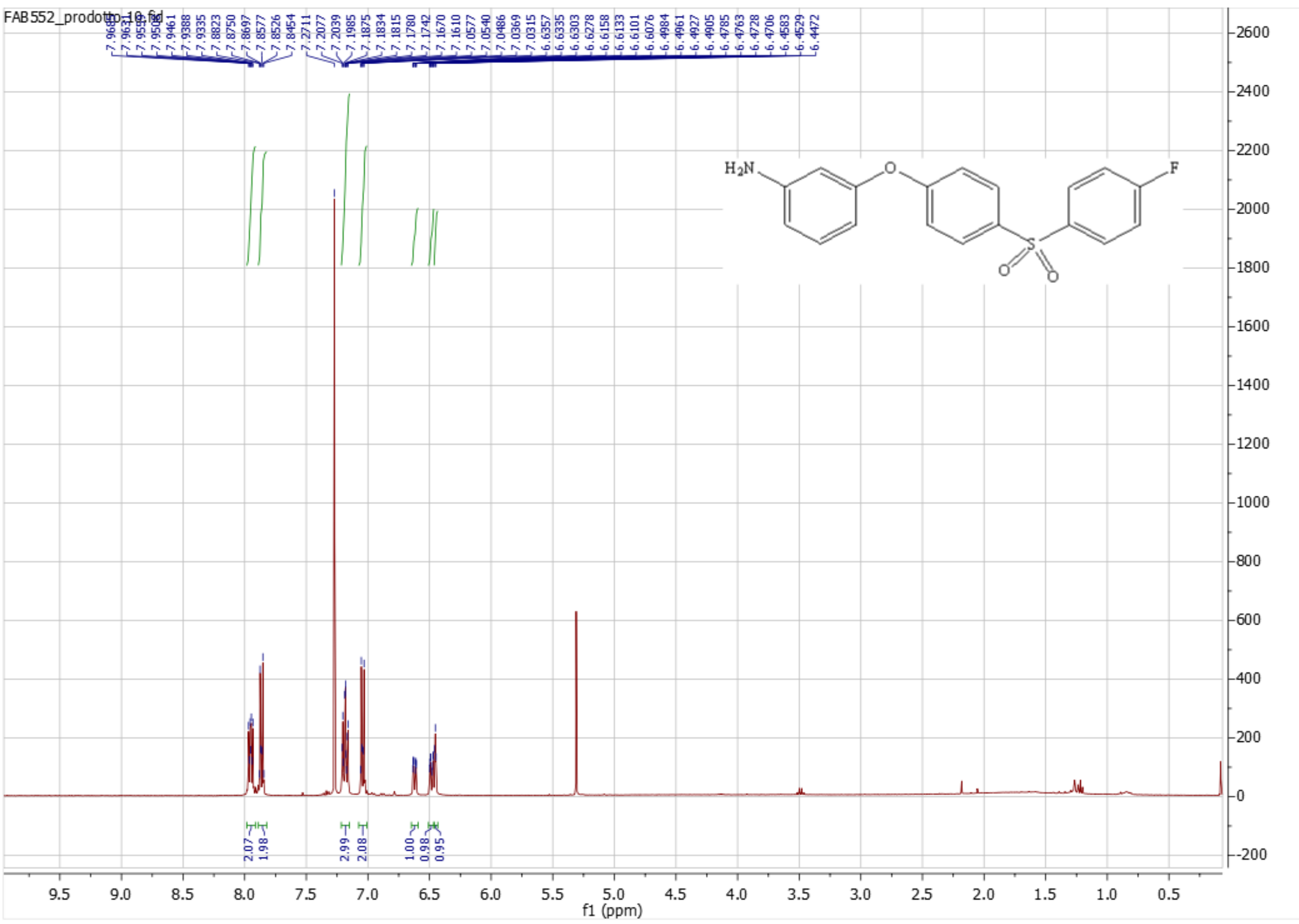

^{1}H NMR spectrum (400 MHz, $CDCl_3$) of compound **11**.

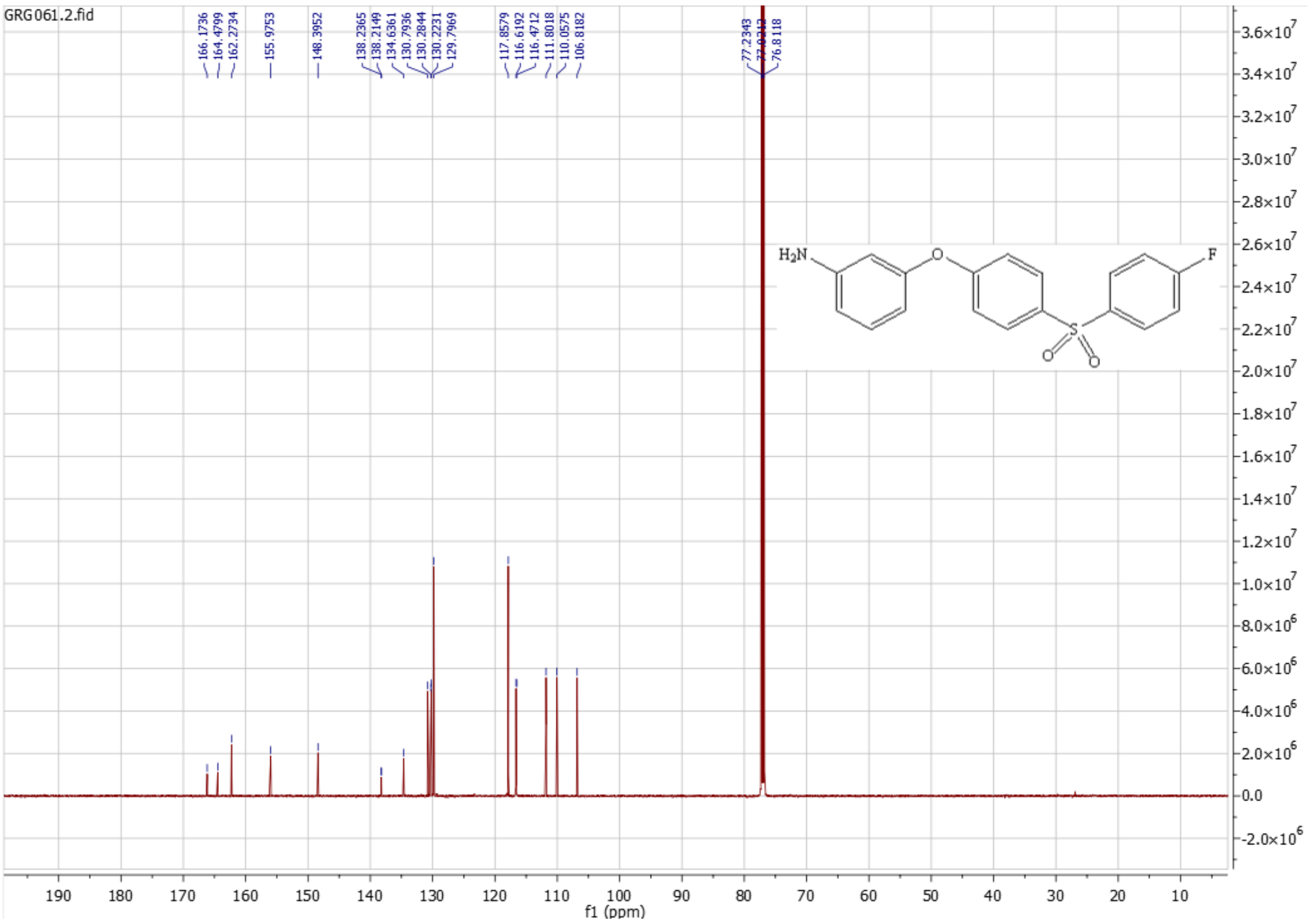

^{13}C NMR spectrum (150 MHz, $CDCl_3$) of compound **11**.

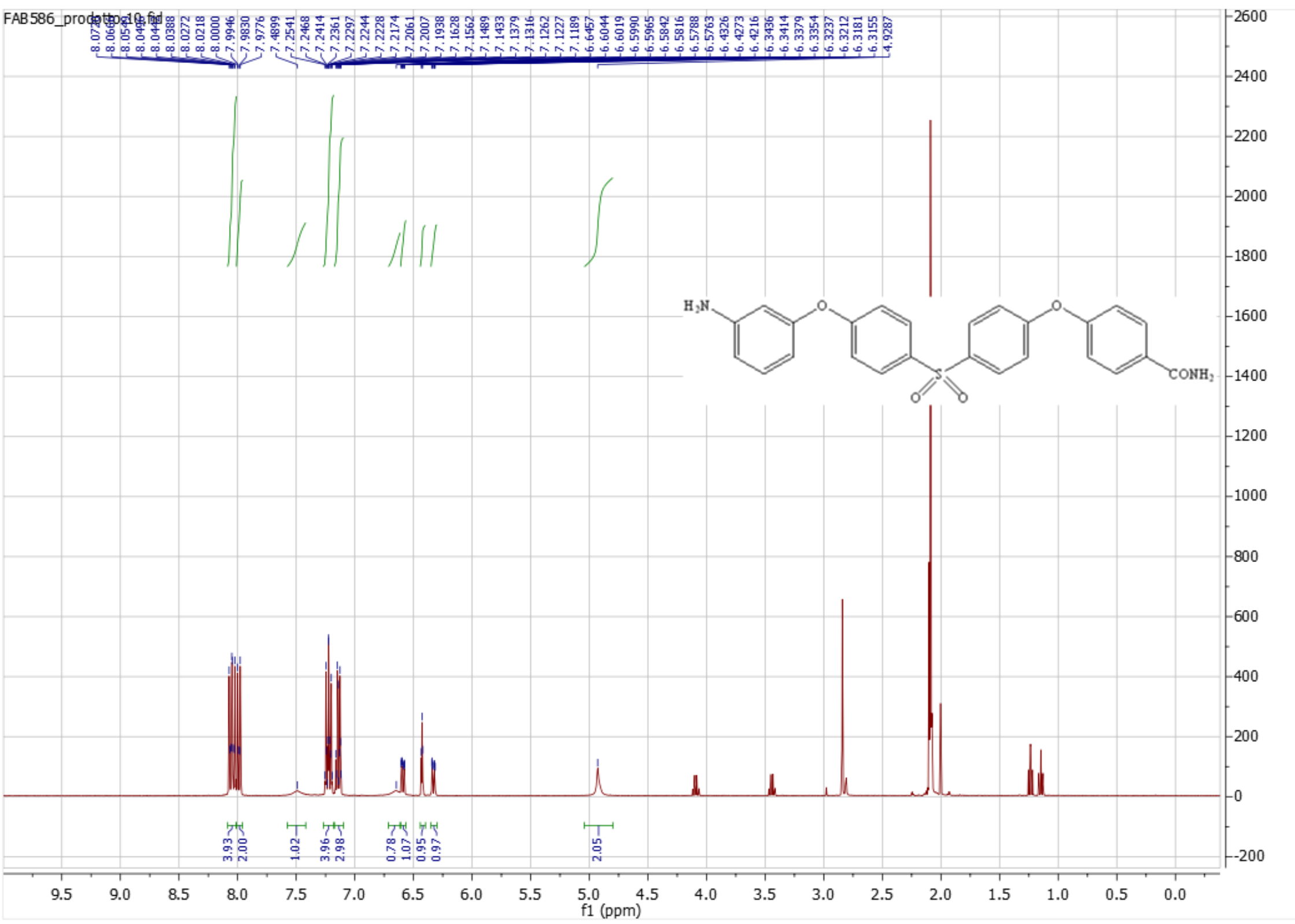

^{1}H NMR spectrum (400 MHz, acetone-d_6) of compound **12**.

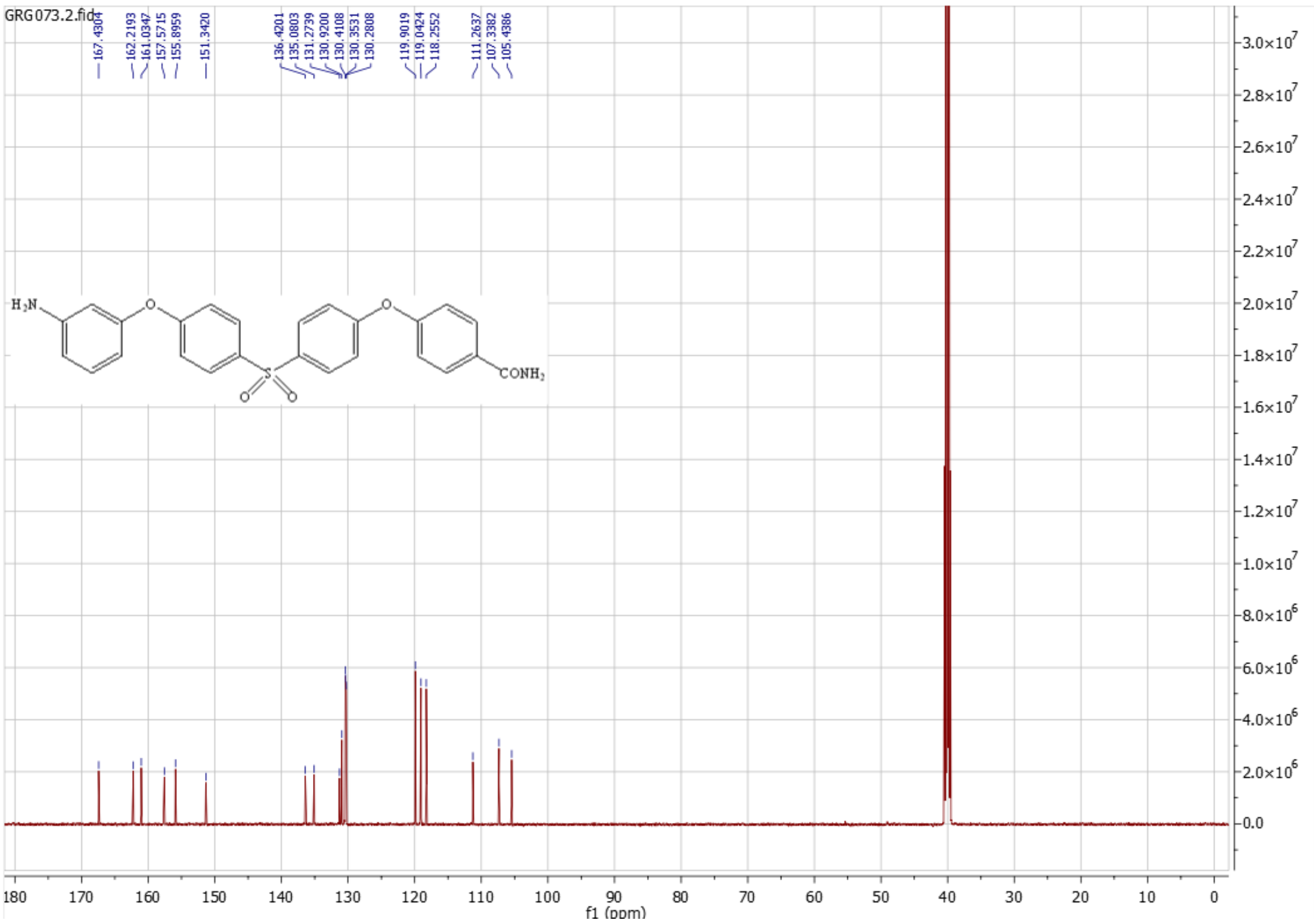

^{13}C NMR spectrum (150 MHz, DMSO-d_6) of compound **12**.

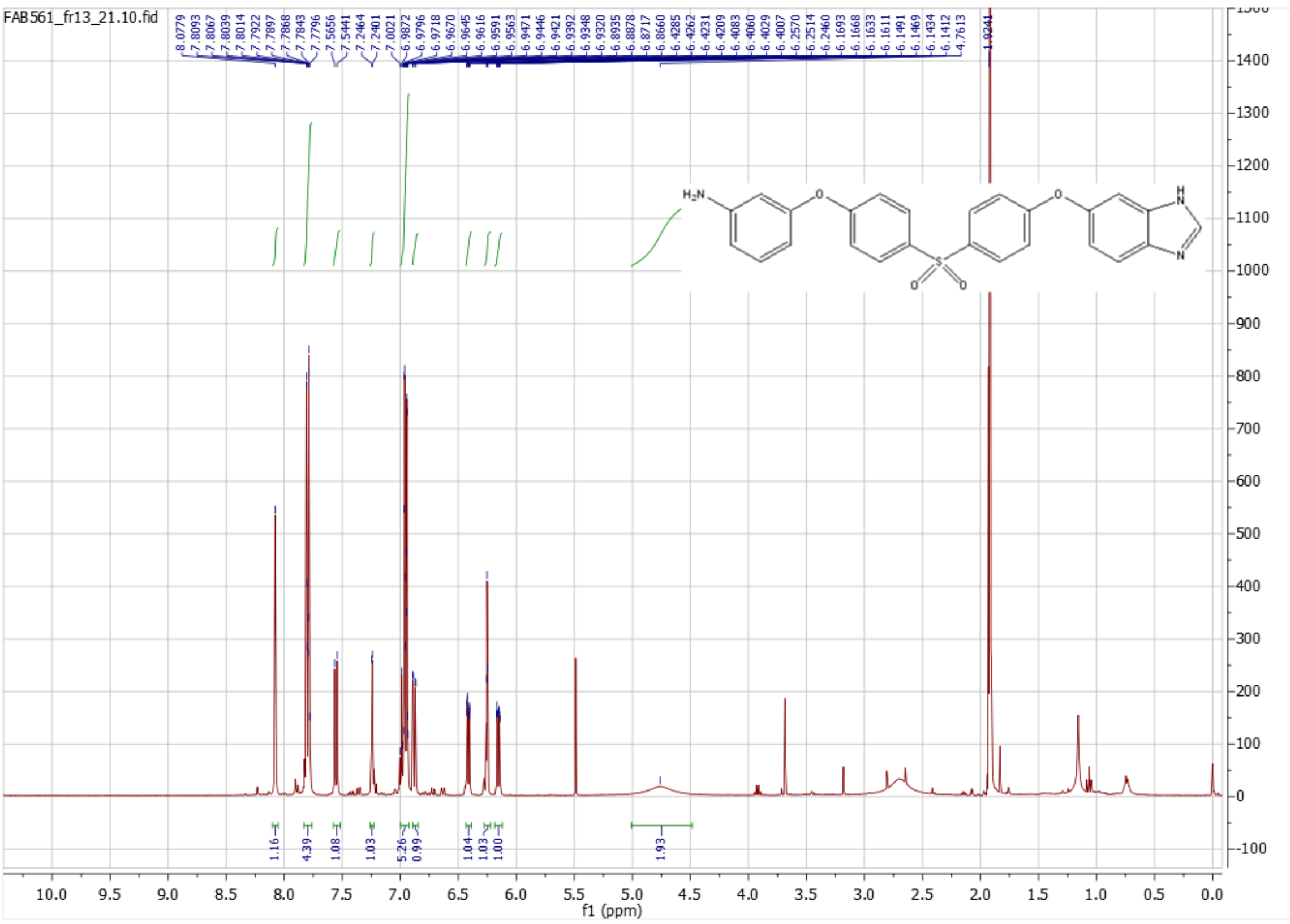

^{1}H NMR spectrum (400 MHz, acetone-d_6) of compound **13**.

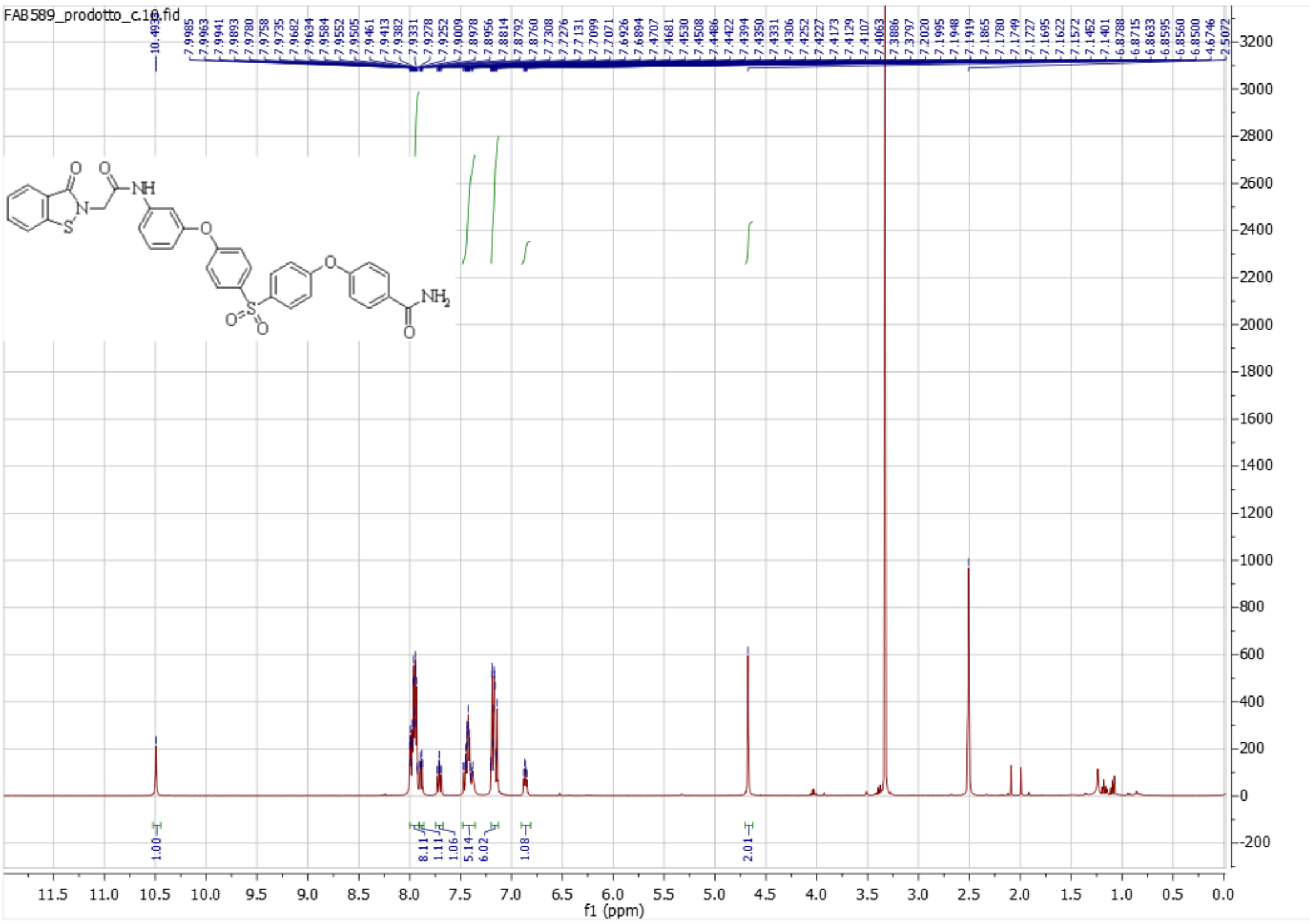

^{1}H NMR spectrum (400 MHz, DMSO-d_6) of compound **14**.

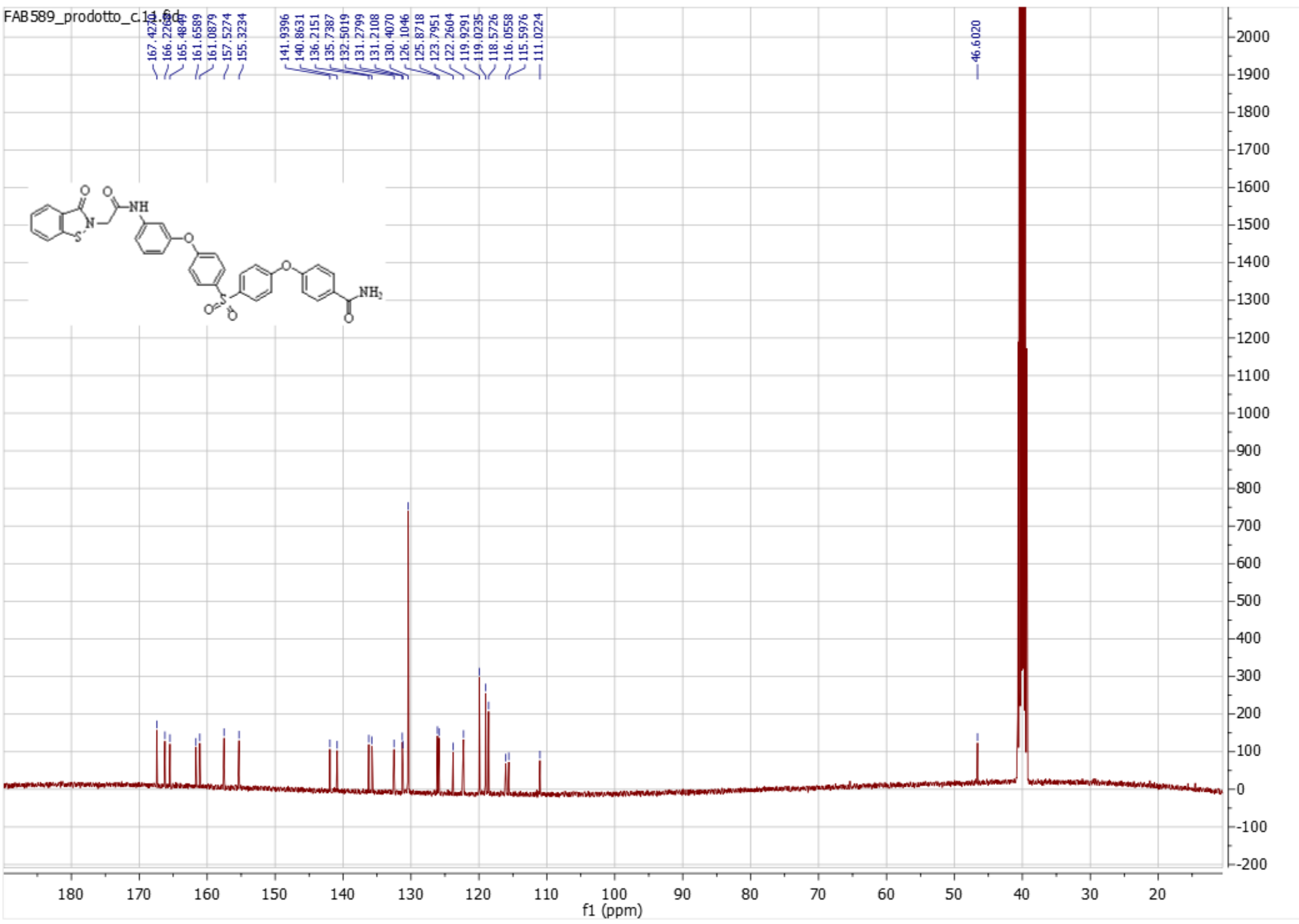

^{13}C NMR spectrum (100 MHz, DMSO-d_6) of compound **14**.

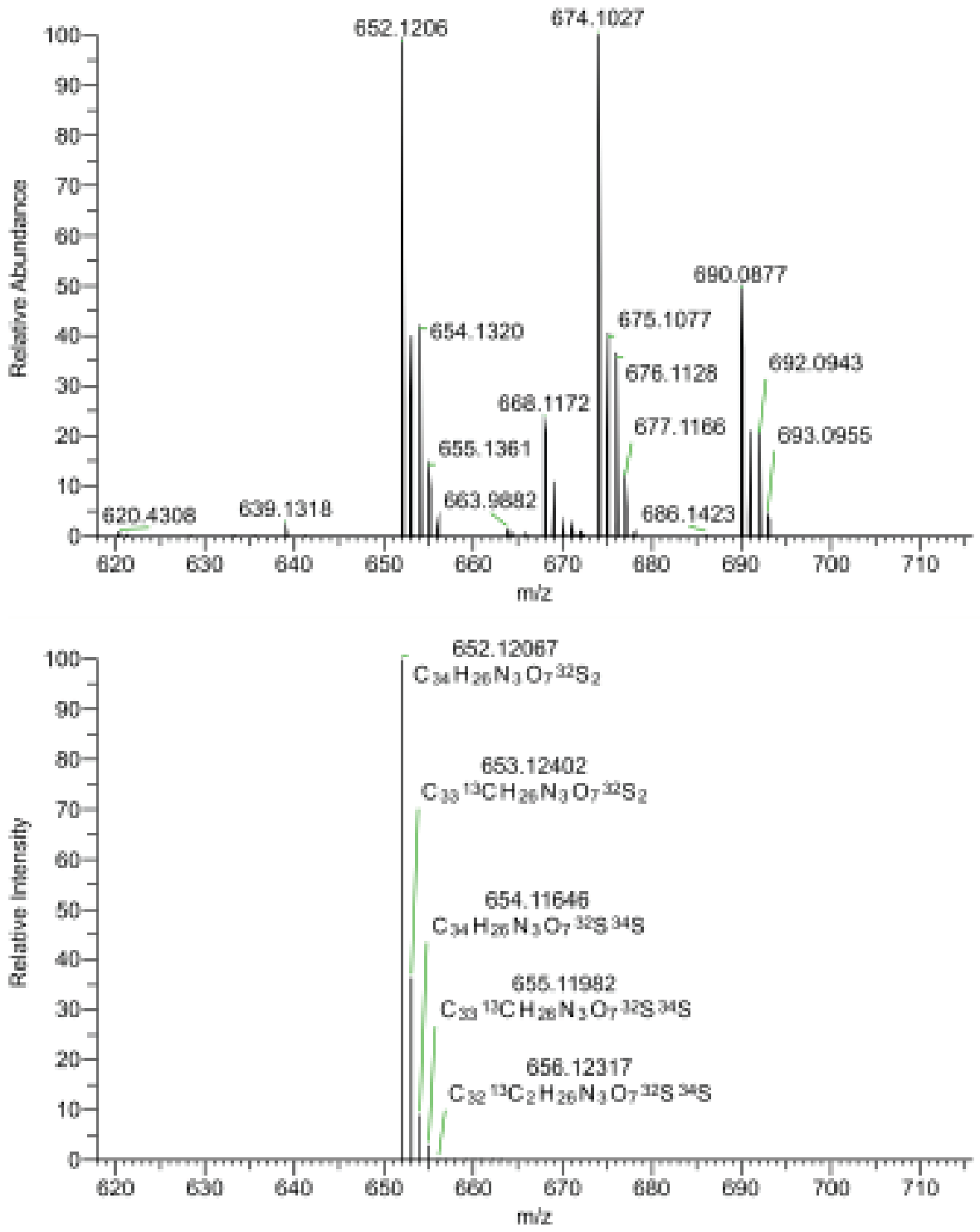

HRMS of compound **14**.

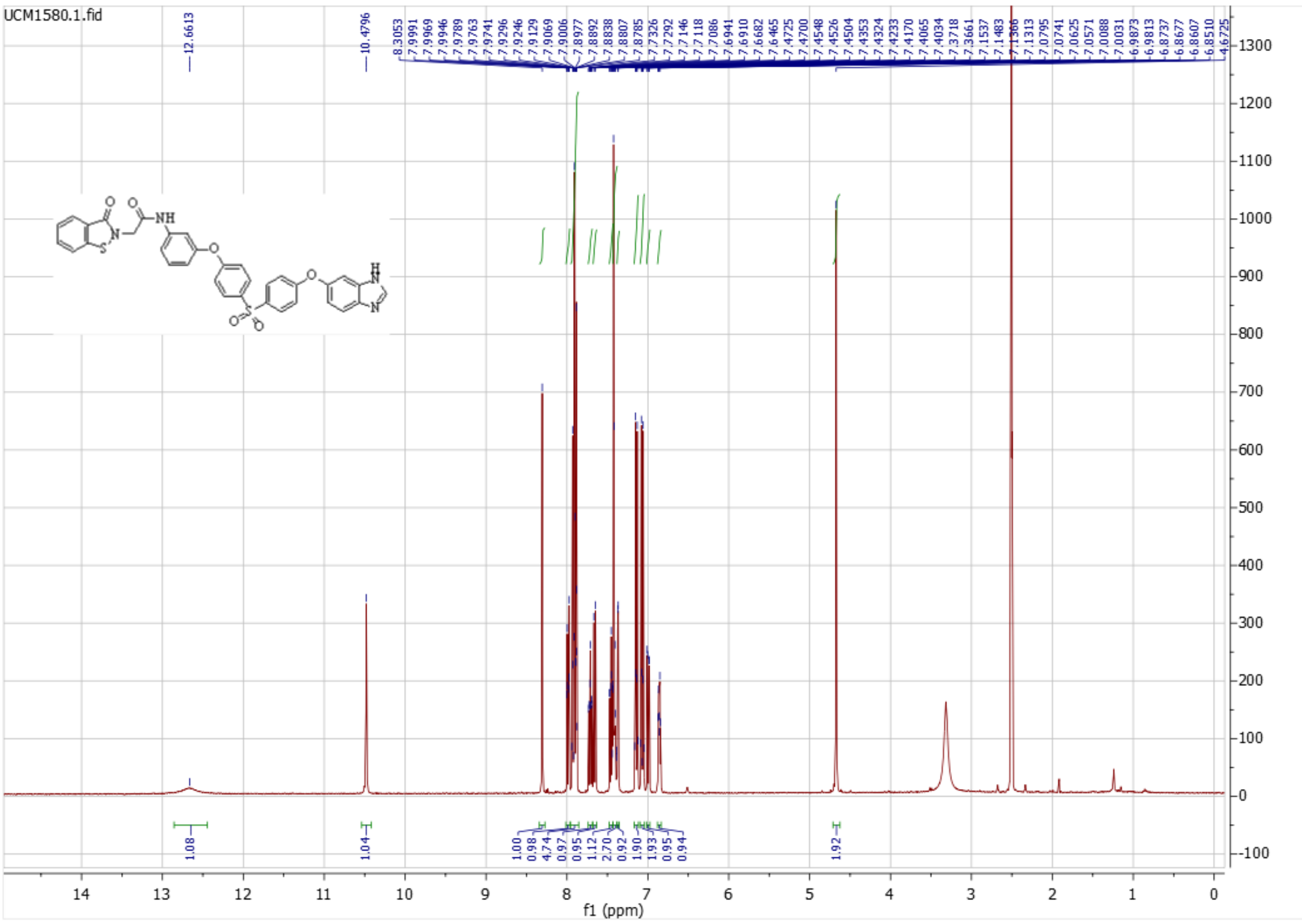

^{1}H NMR spectrum (400 MHz, DMSO-d_6) of compound **15**.

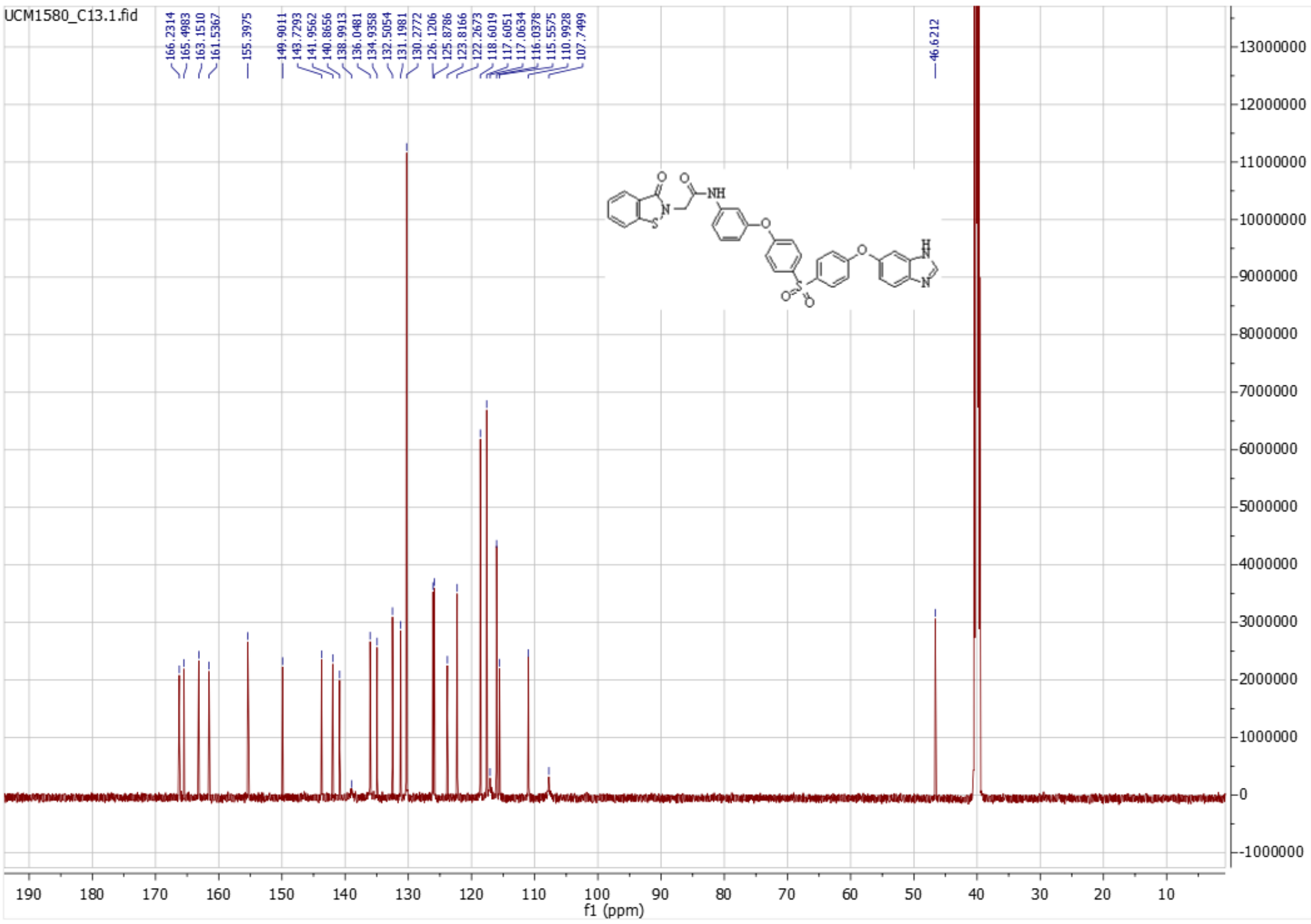

^{13}C NMR spectrum (150 MHz, DMSO-d_6) of compound **15**.

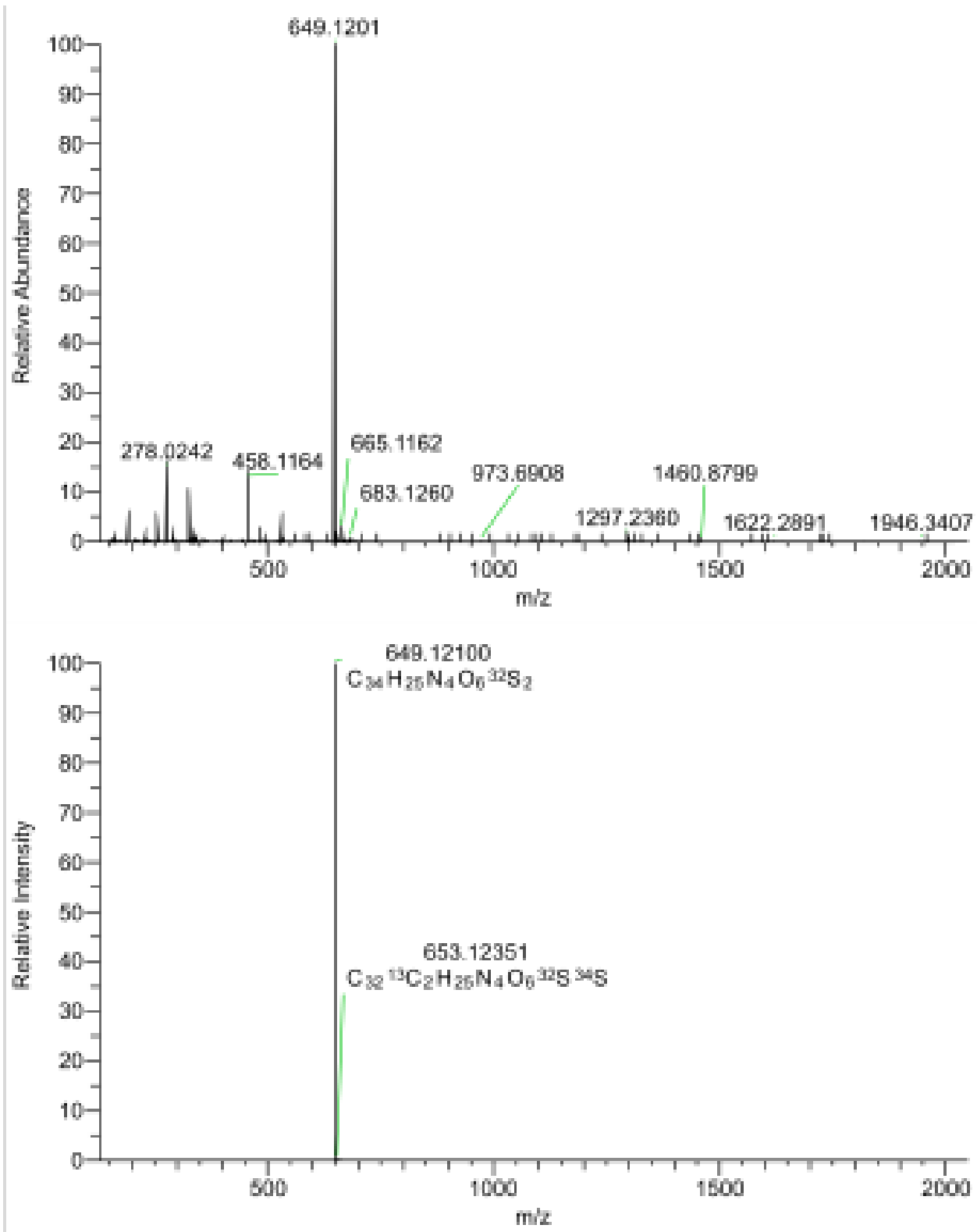

HRMS of compound **15**.

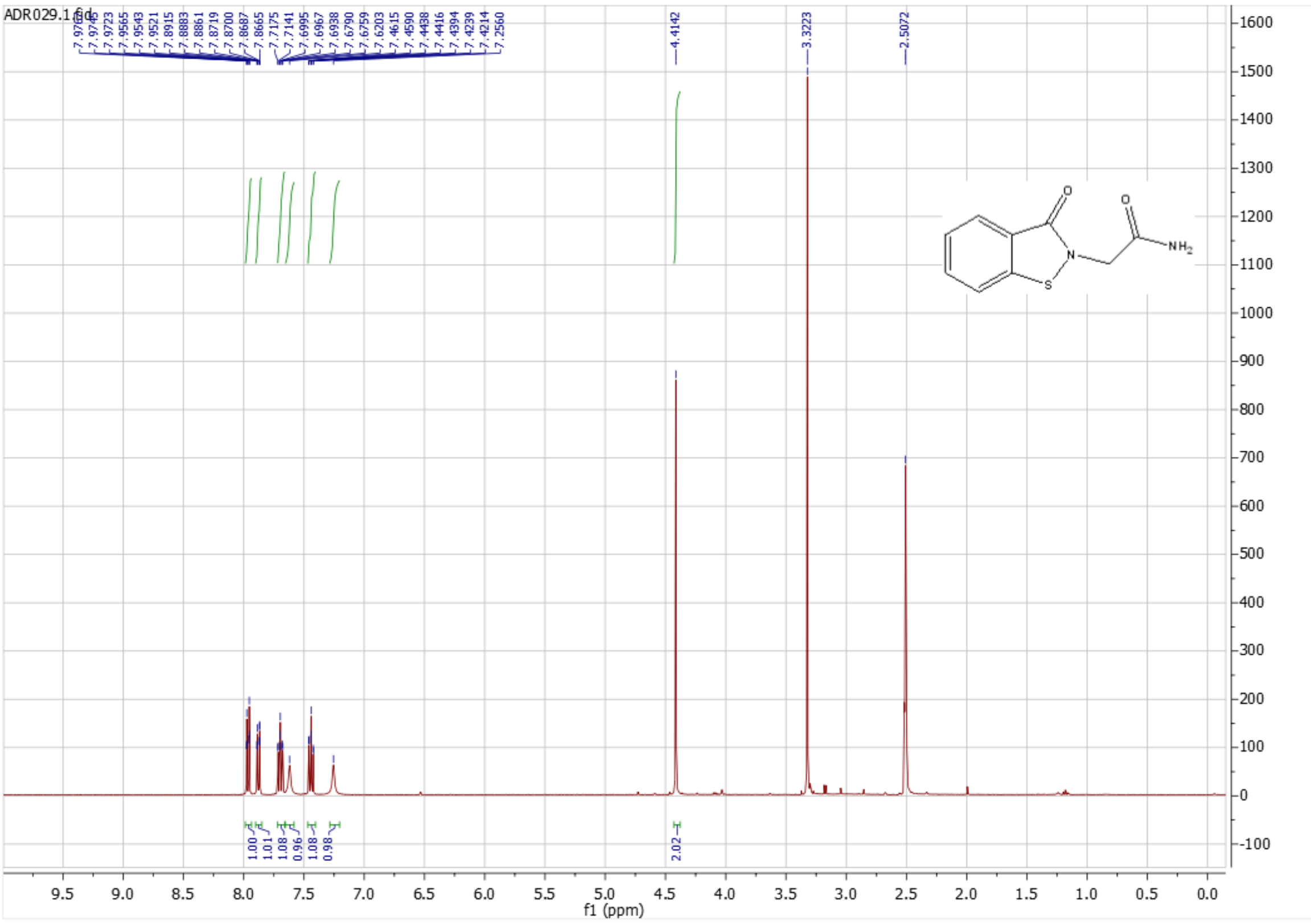

^{1}H NMR spectrum (400 MHz, DMSO-d_6) of compound **16**.

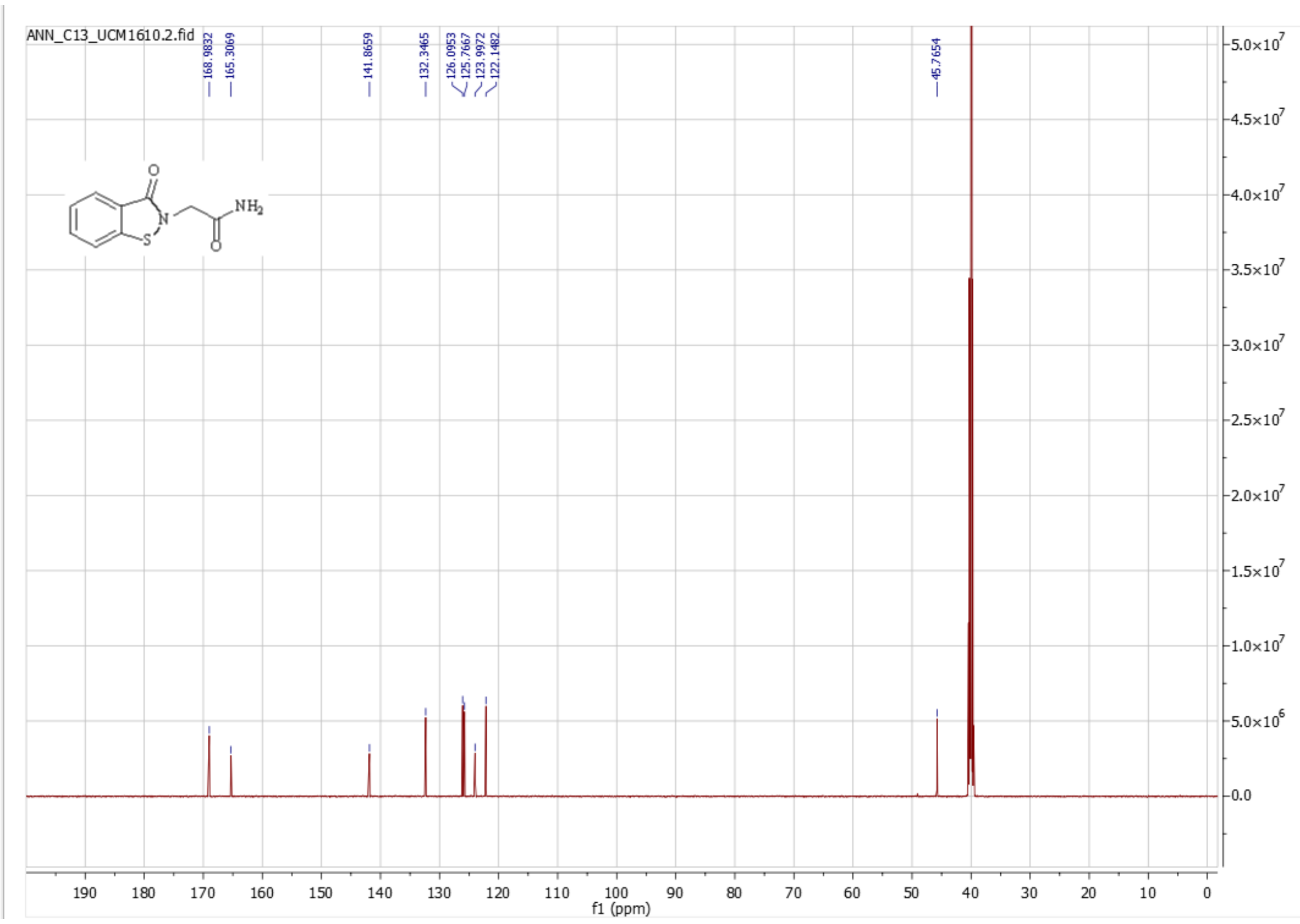

^{13}C NMR spectrum (150 MHz, DMSO-d_6) of compound **16**.

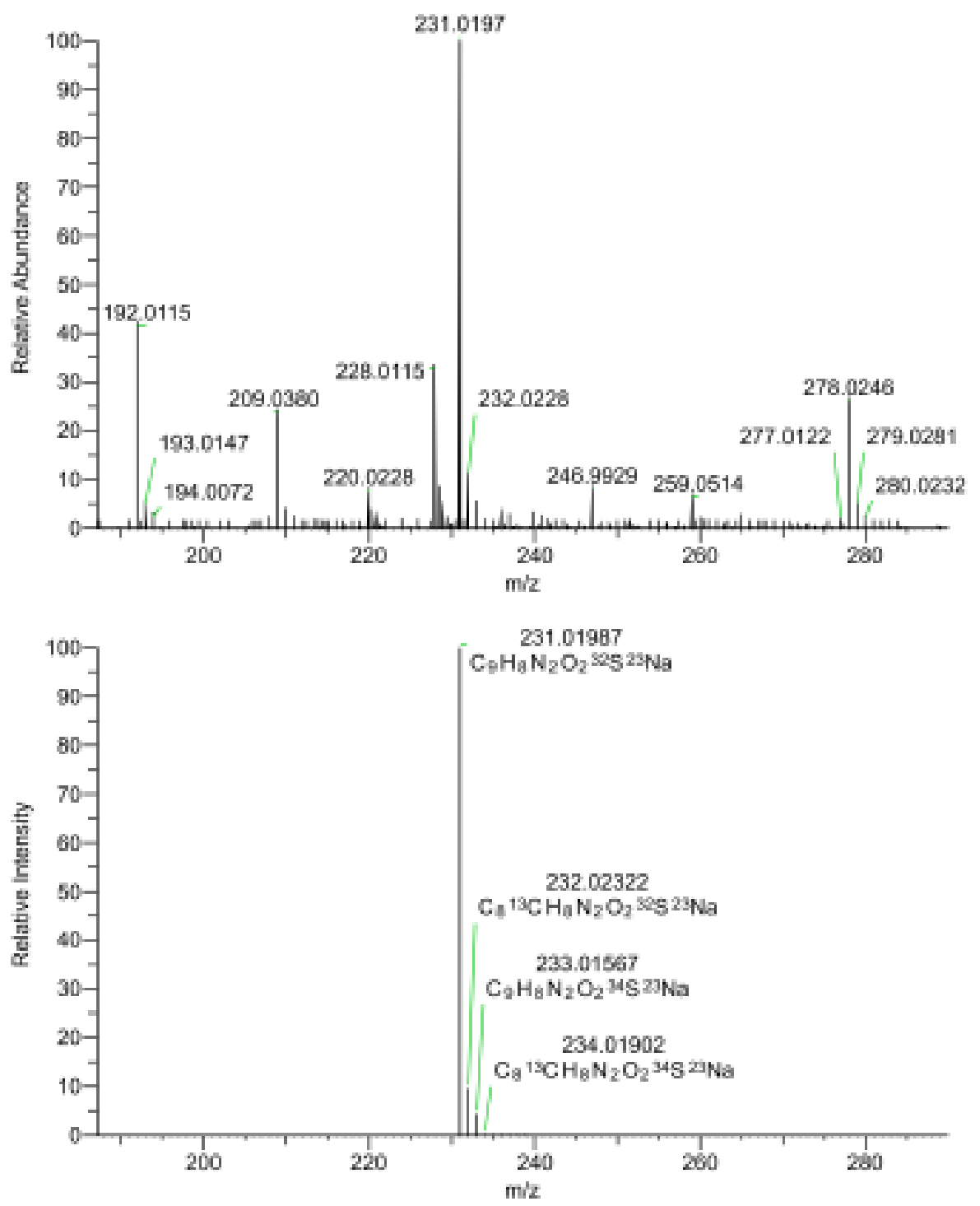

HRMS of compound **16**.

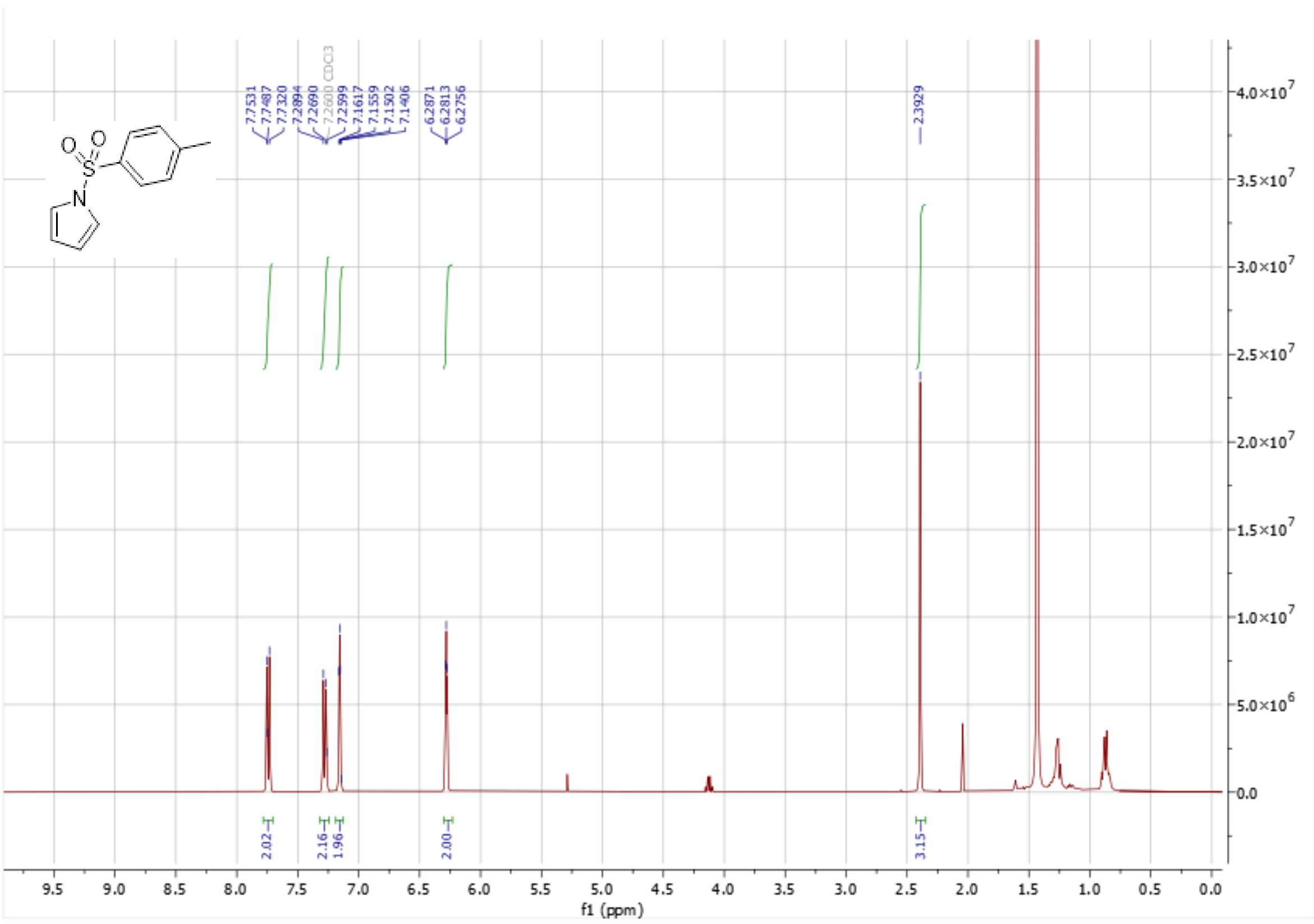

^{1}H NMR spectrum (400 MHz, $CDCl_3$) of compound **17**.

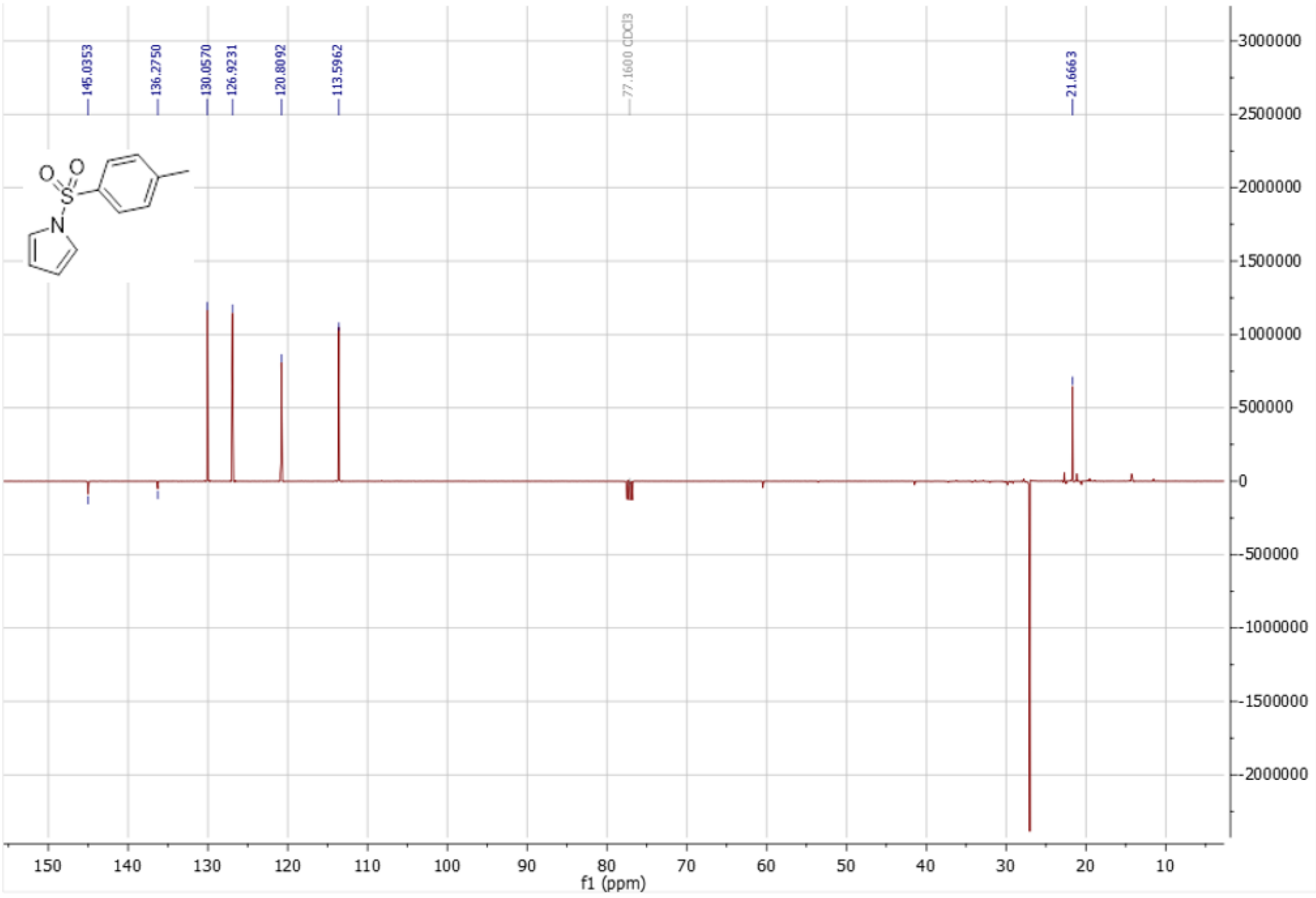

^{13}C NMR spectrum (101 MHz, $CDCl_3$) of compound **17**.

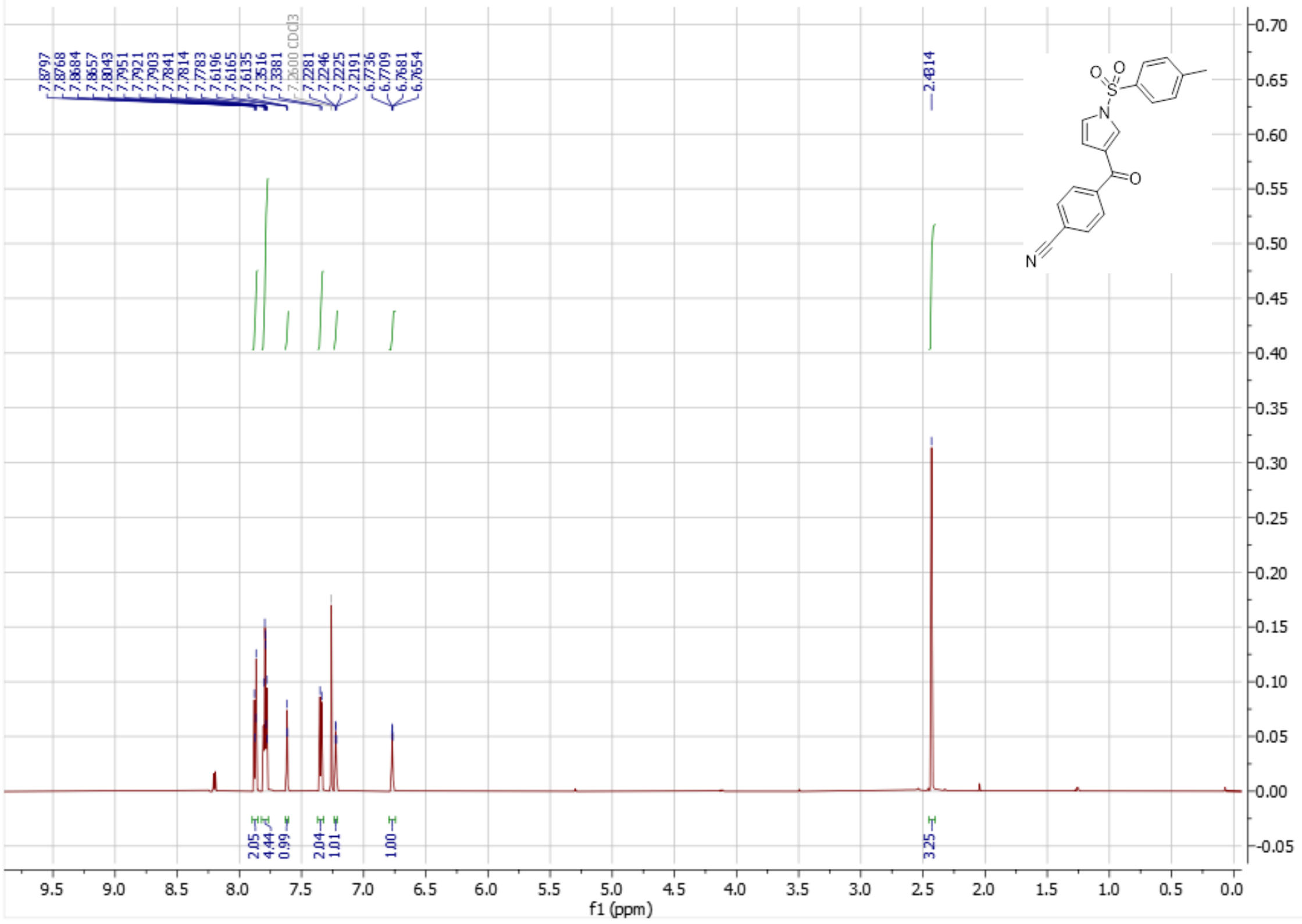

^{1}H NMR spectrum (600 MHz, $CDCl_3$) of compound **18**.

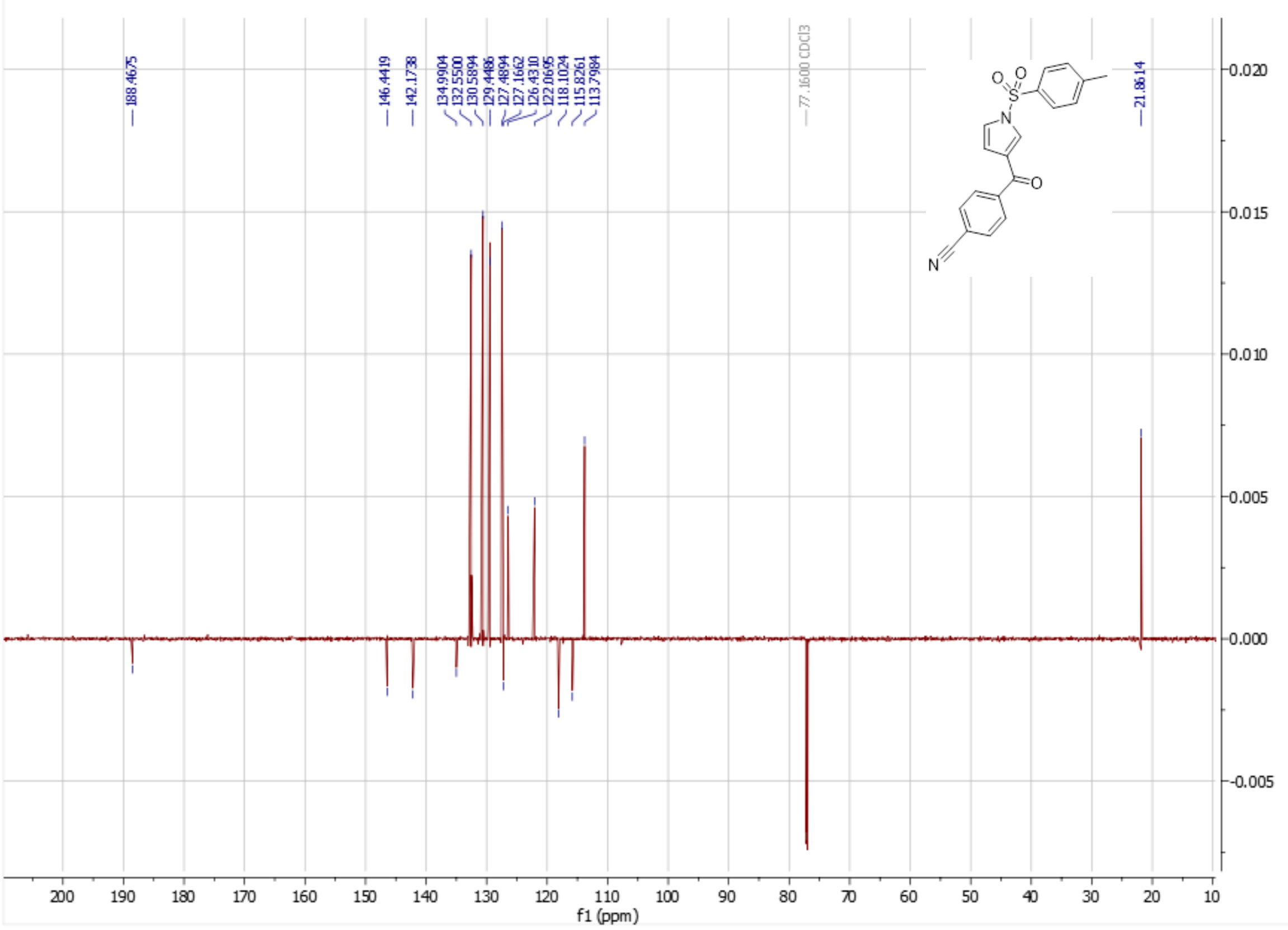

^{13}C NMR spectrum (151 MHz, $CDCl_3$) of compound **18**.

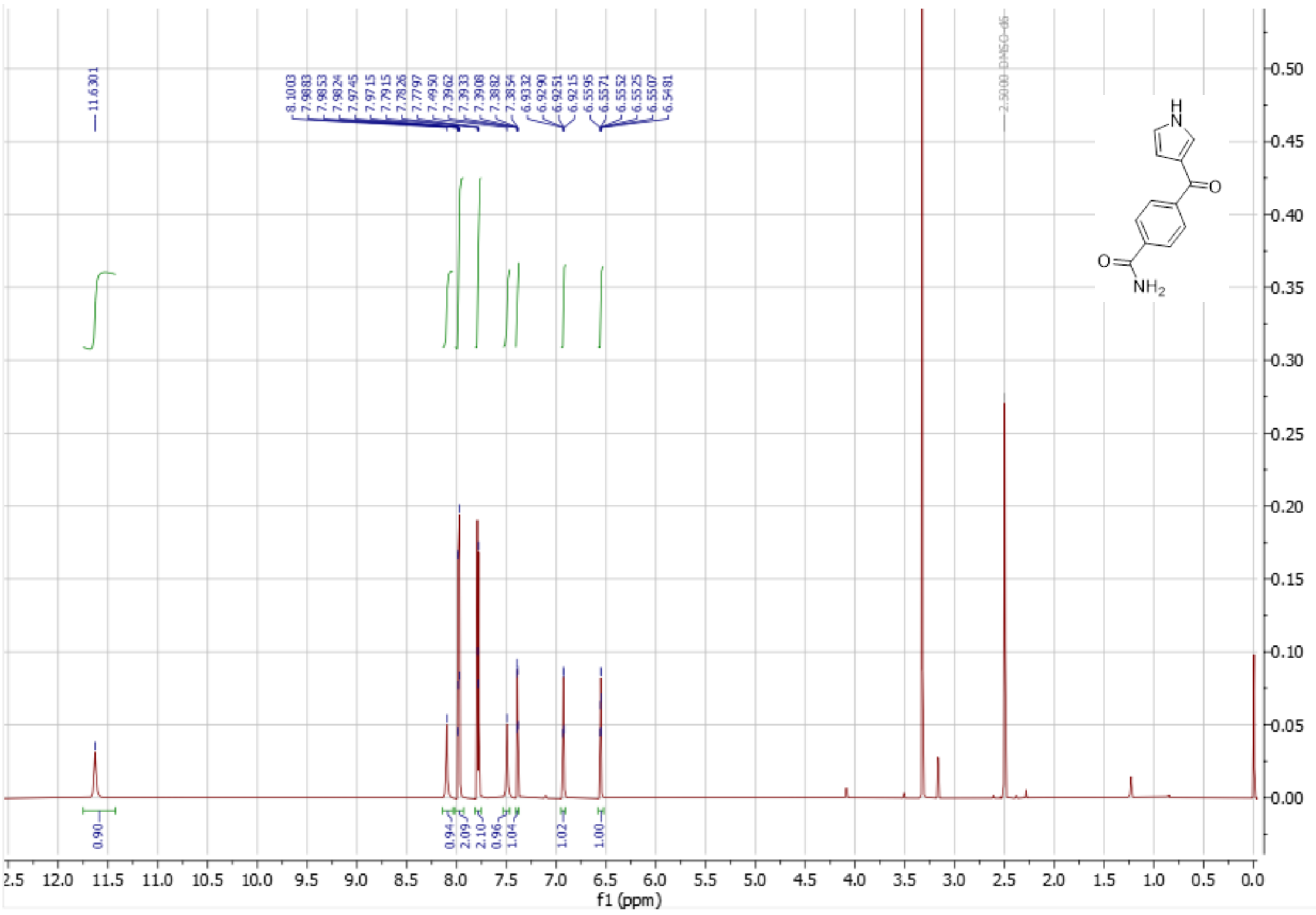

^{1}H NMR spectrum (400 MHz, DMSO-d_6) of compound **19**.

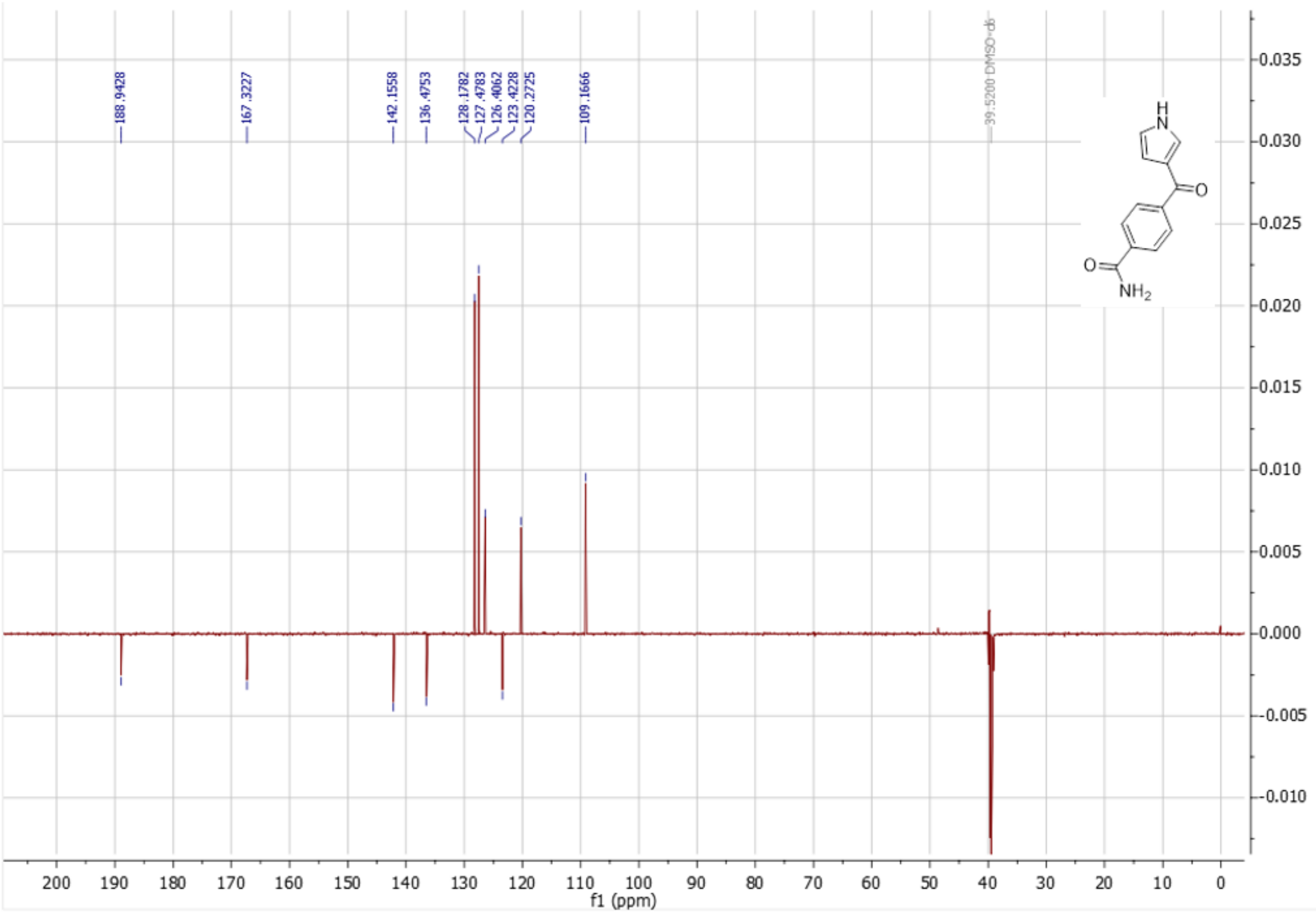

^{13}C NMR spectrum (151 MHz, DMSO-d_6) of compound **19**.

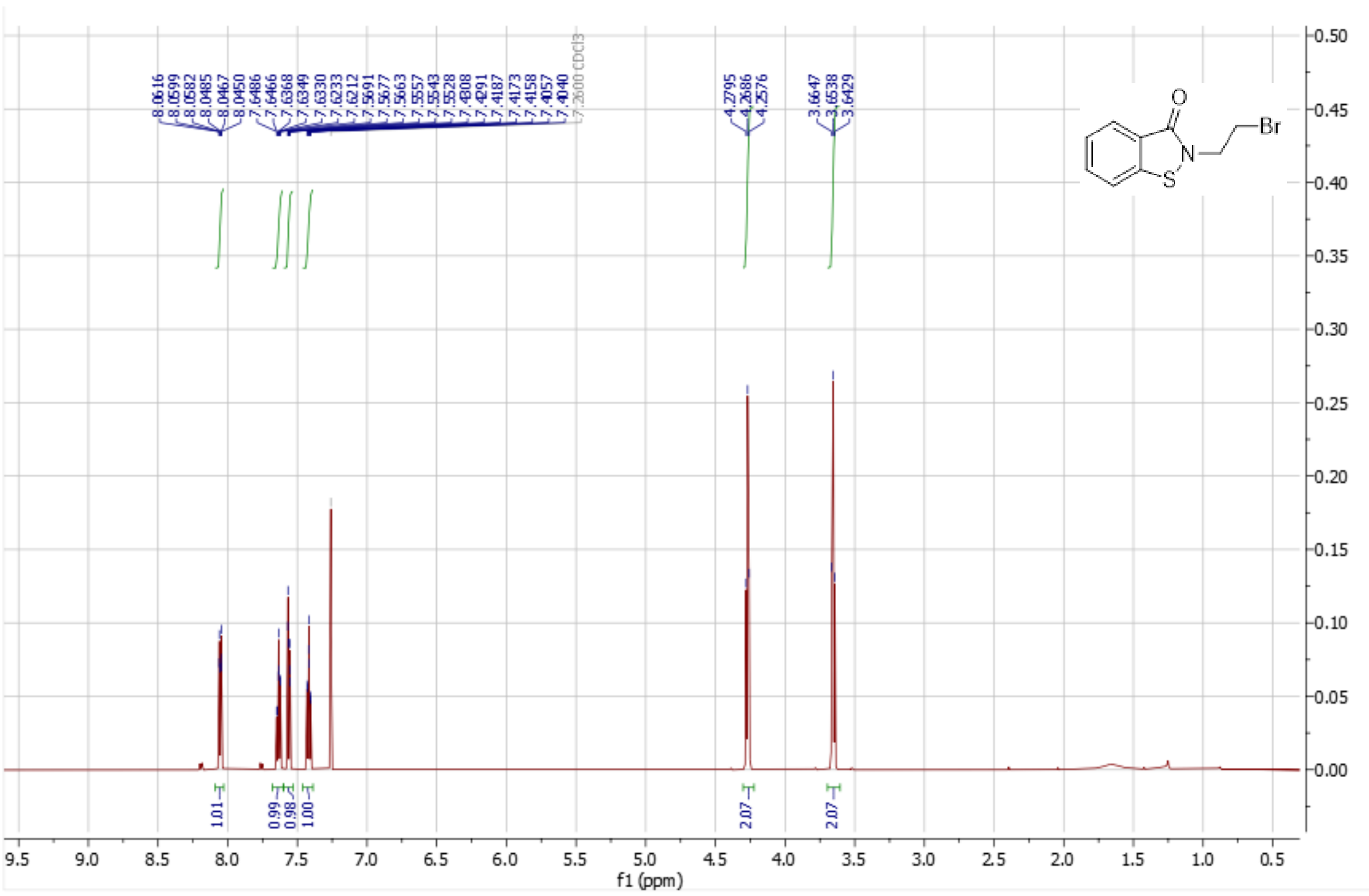

^{1}H NMR spectrum (600 MHz, $CDCl_3$) of compound **20**.

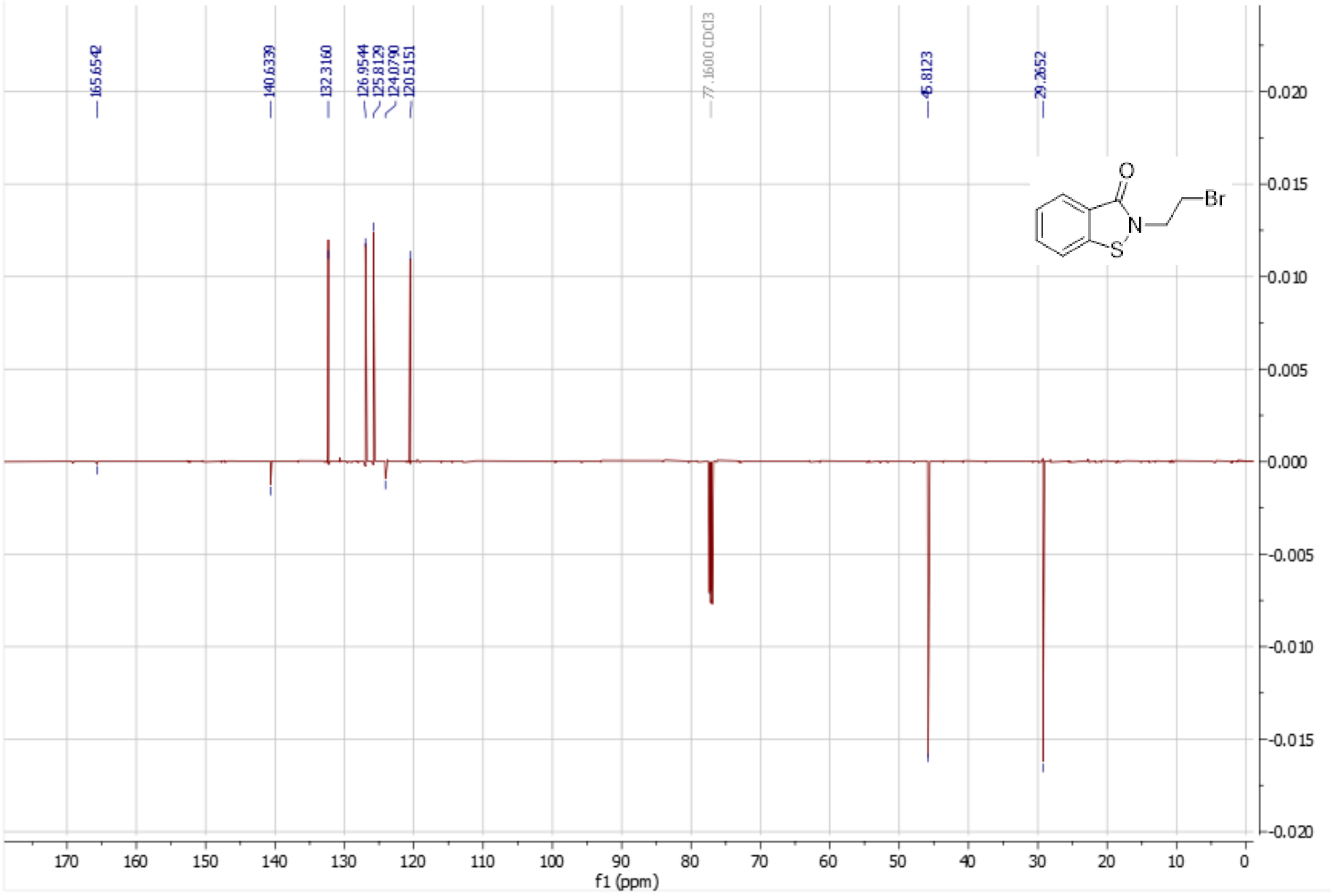

^{13}C NMR spectrum (151 MHz, $CDCl_3$) of compound **20**.

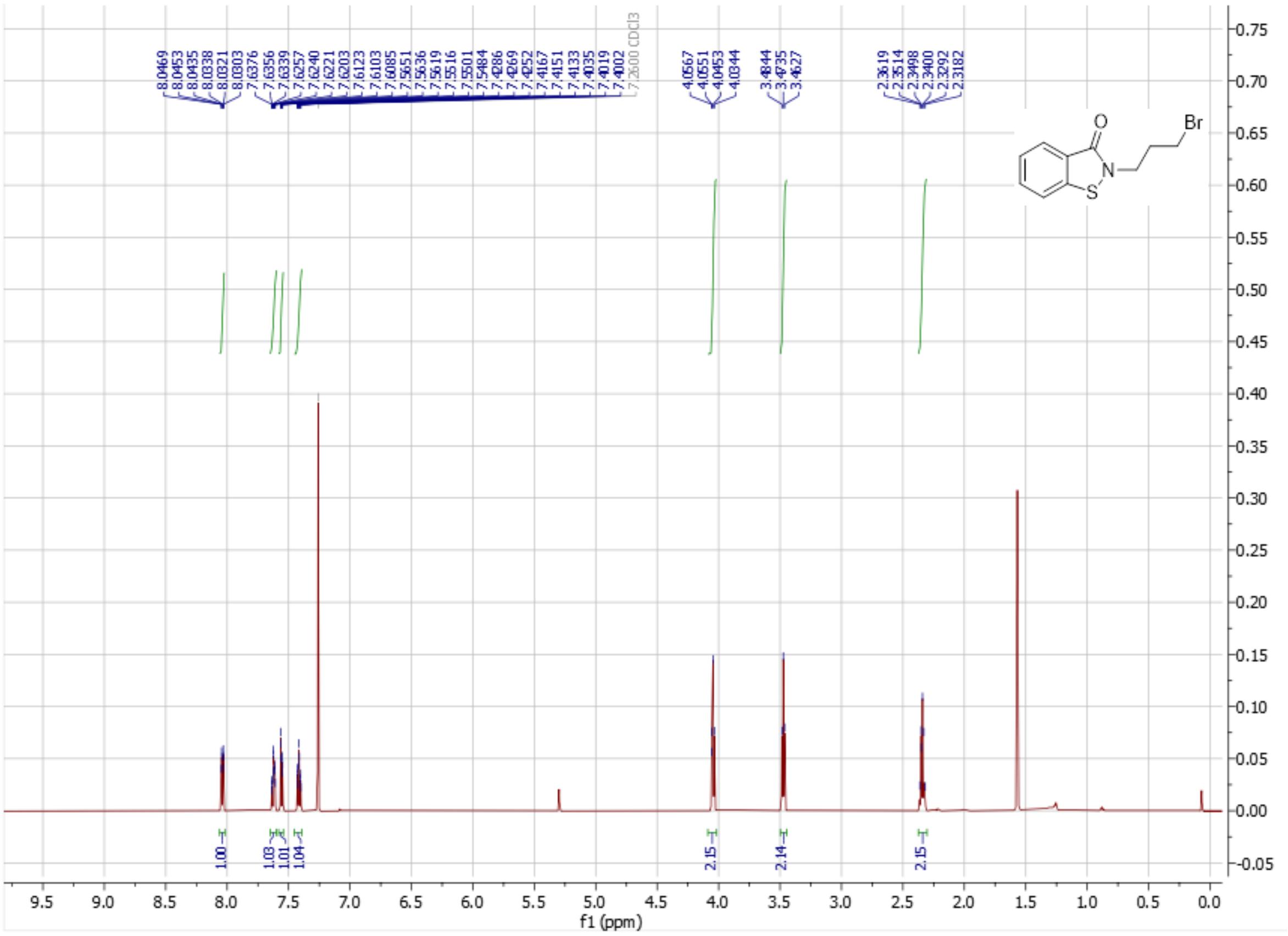

^{1}H NMR spectrum (600 MHz, $CDCl_3$) of compound **21**.

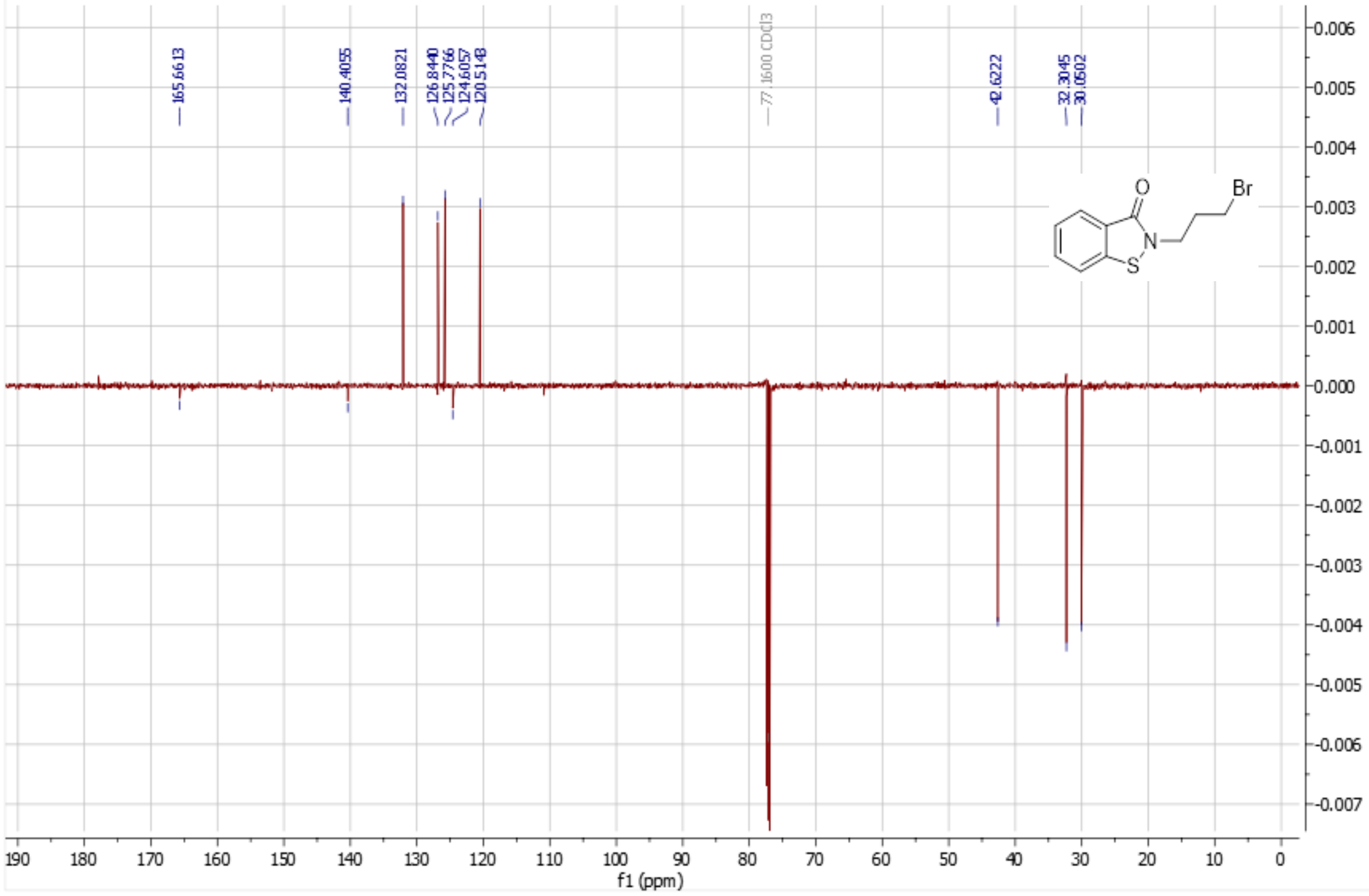

^{13}C NMR spectrum (151 MHz, $CDCl_3$) of compound **21**.

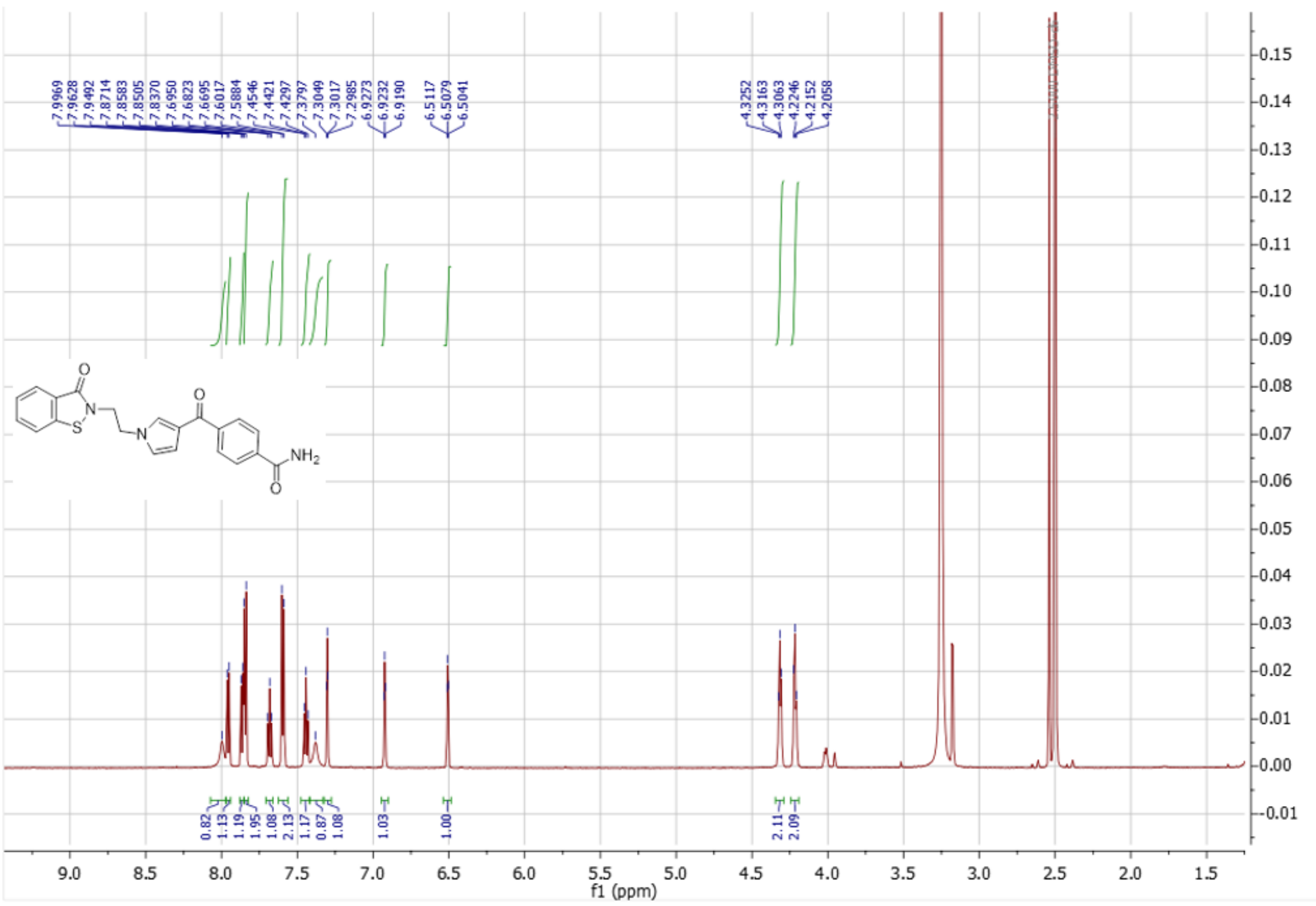

^{1}H NMR spectrum (600 MHz, DMSO-d_6) of compound **22**.

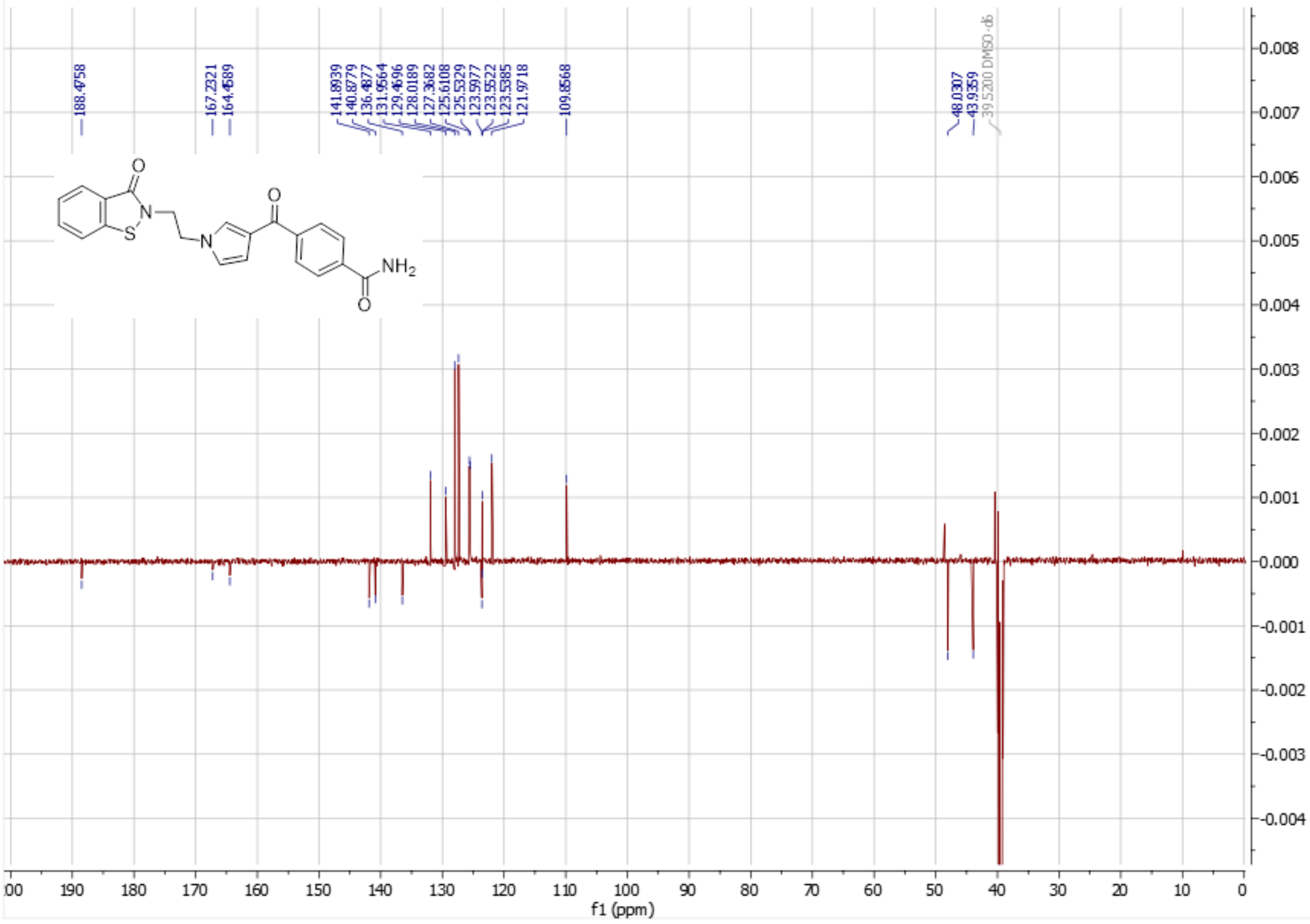

^{13}C NMR spectrum (151 MHz, DMSO-d_6) of compound **22**.

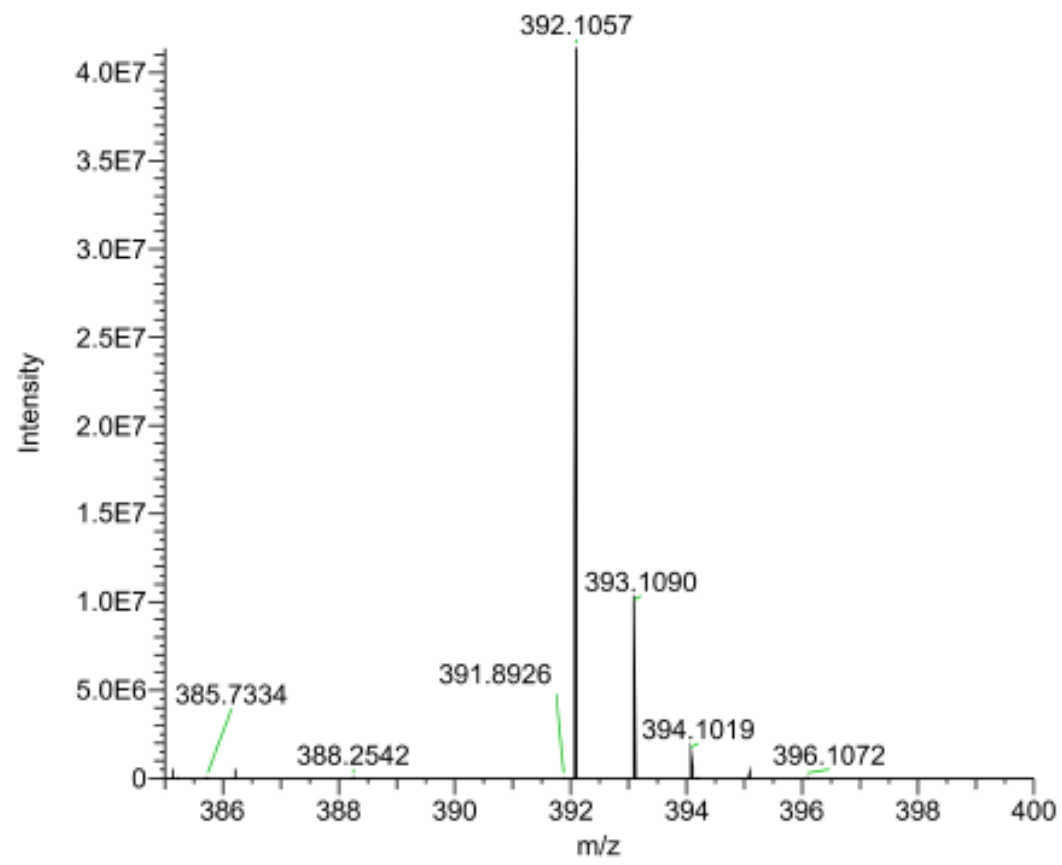

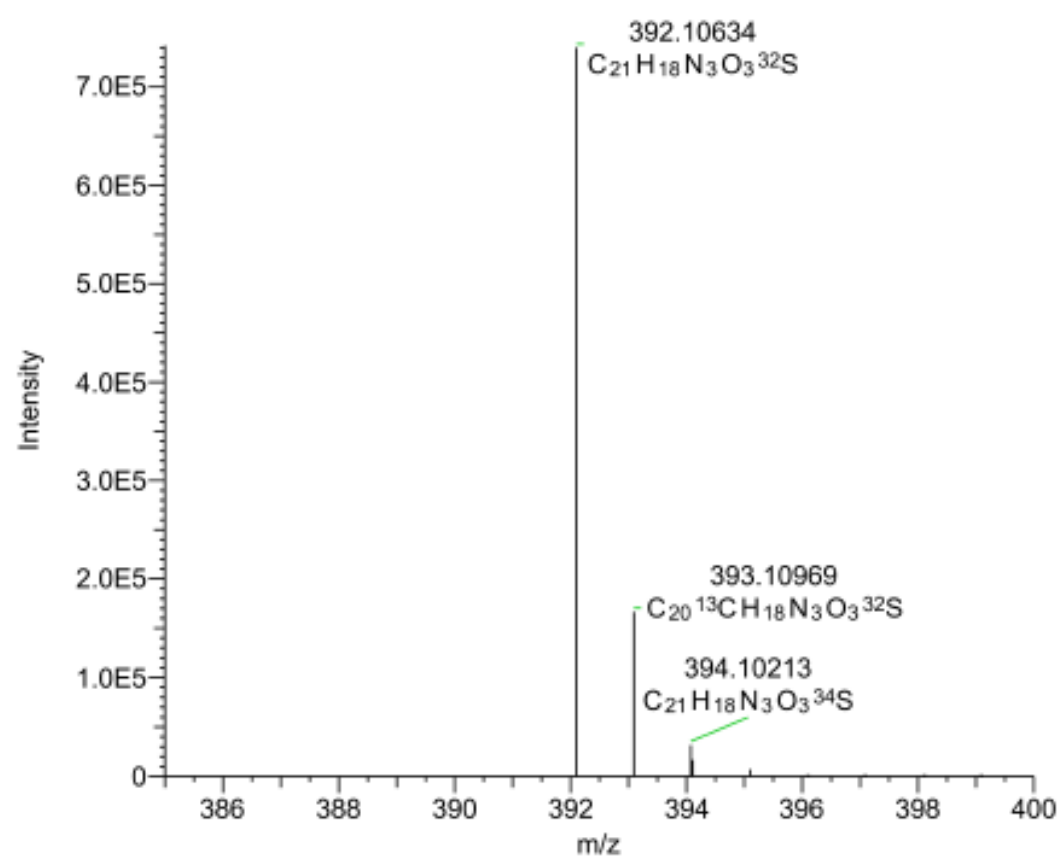

HRMS of compound **22**.

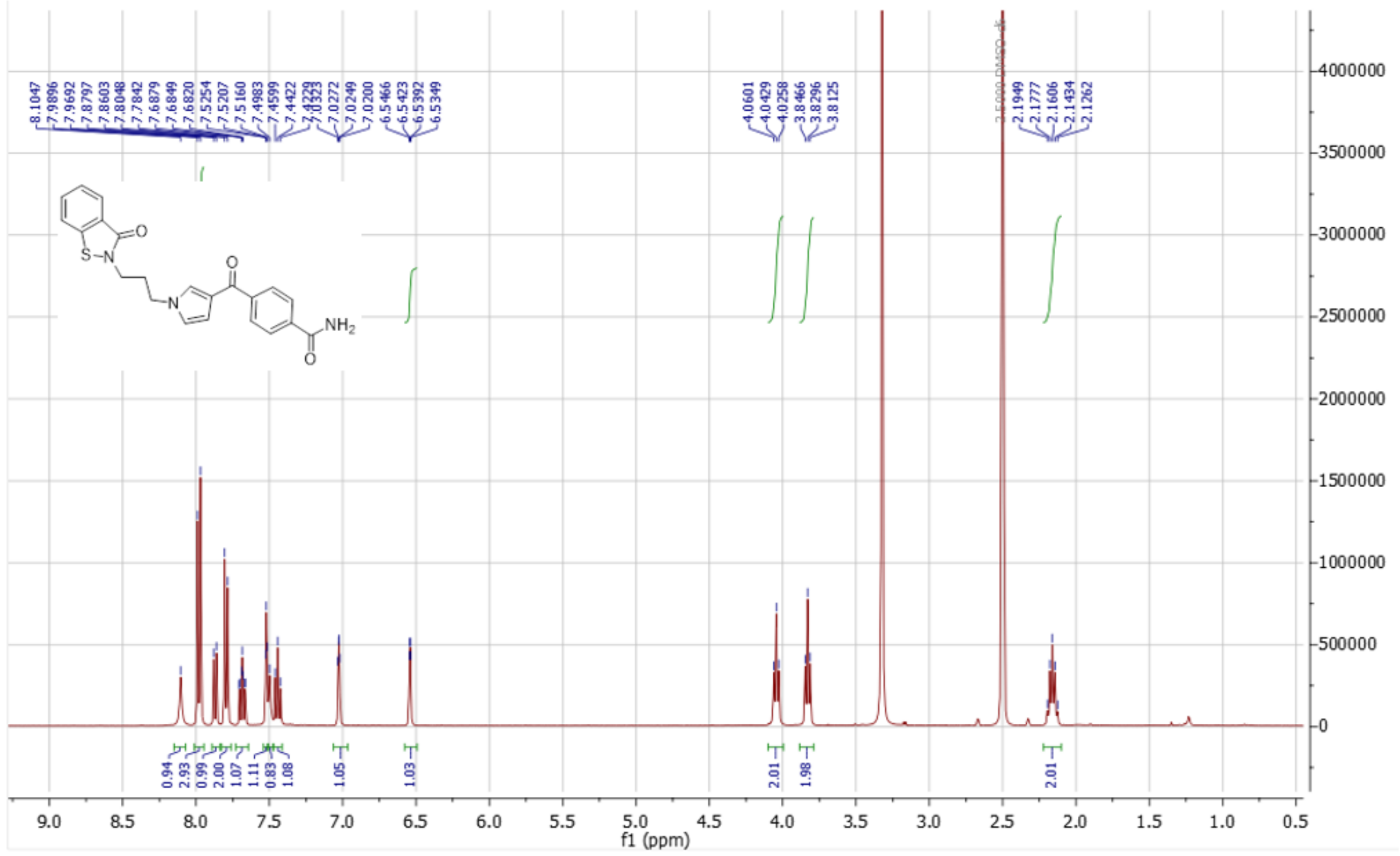

^{1}H NMR spectrum (400 MHz, DMSO-d_6) of compound **23**.

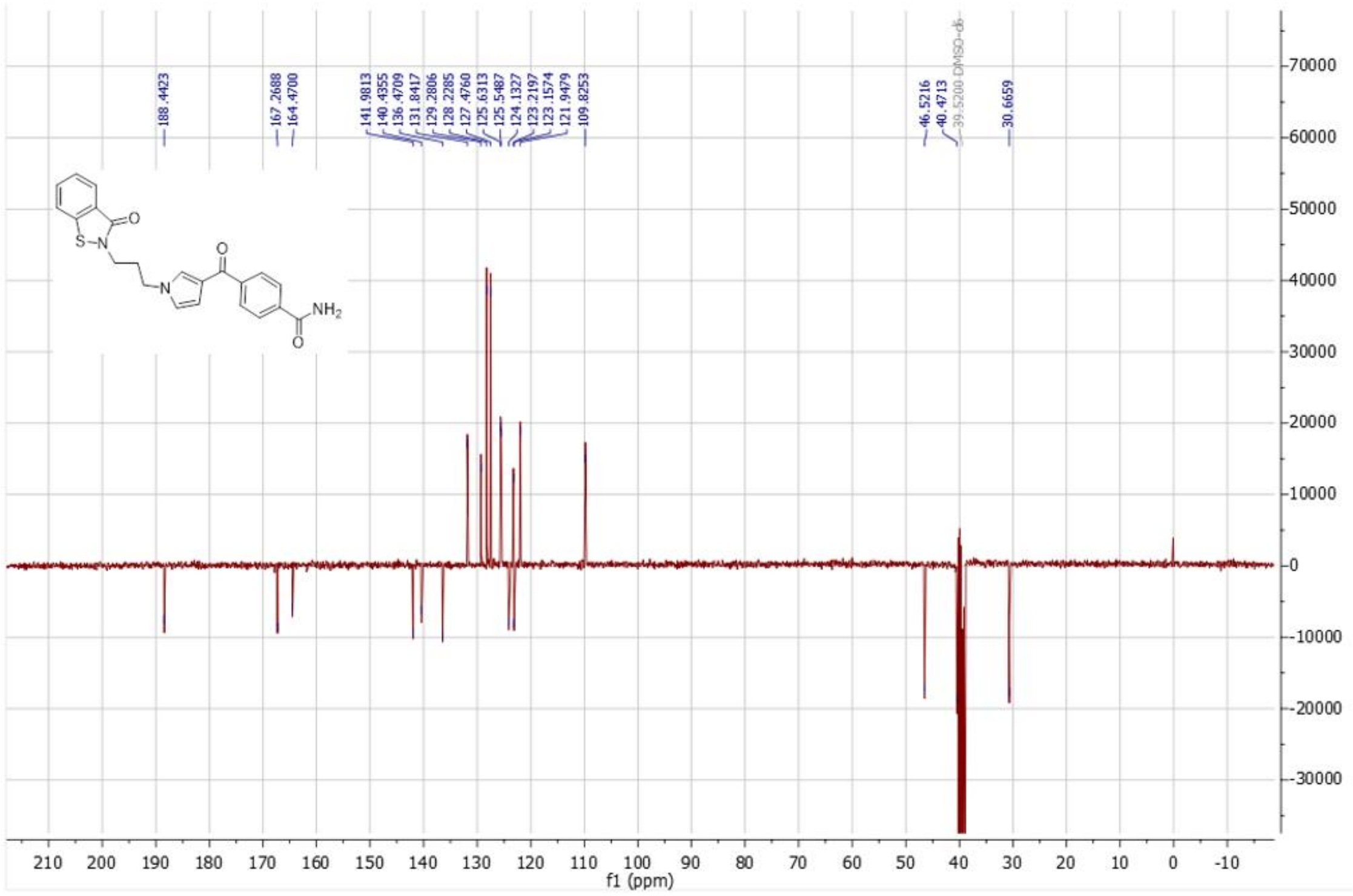

^{13}C NMR spectrum (101 MHz, DMSO-d_6) of compound **23**.

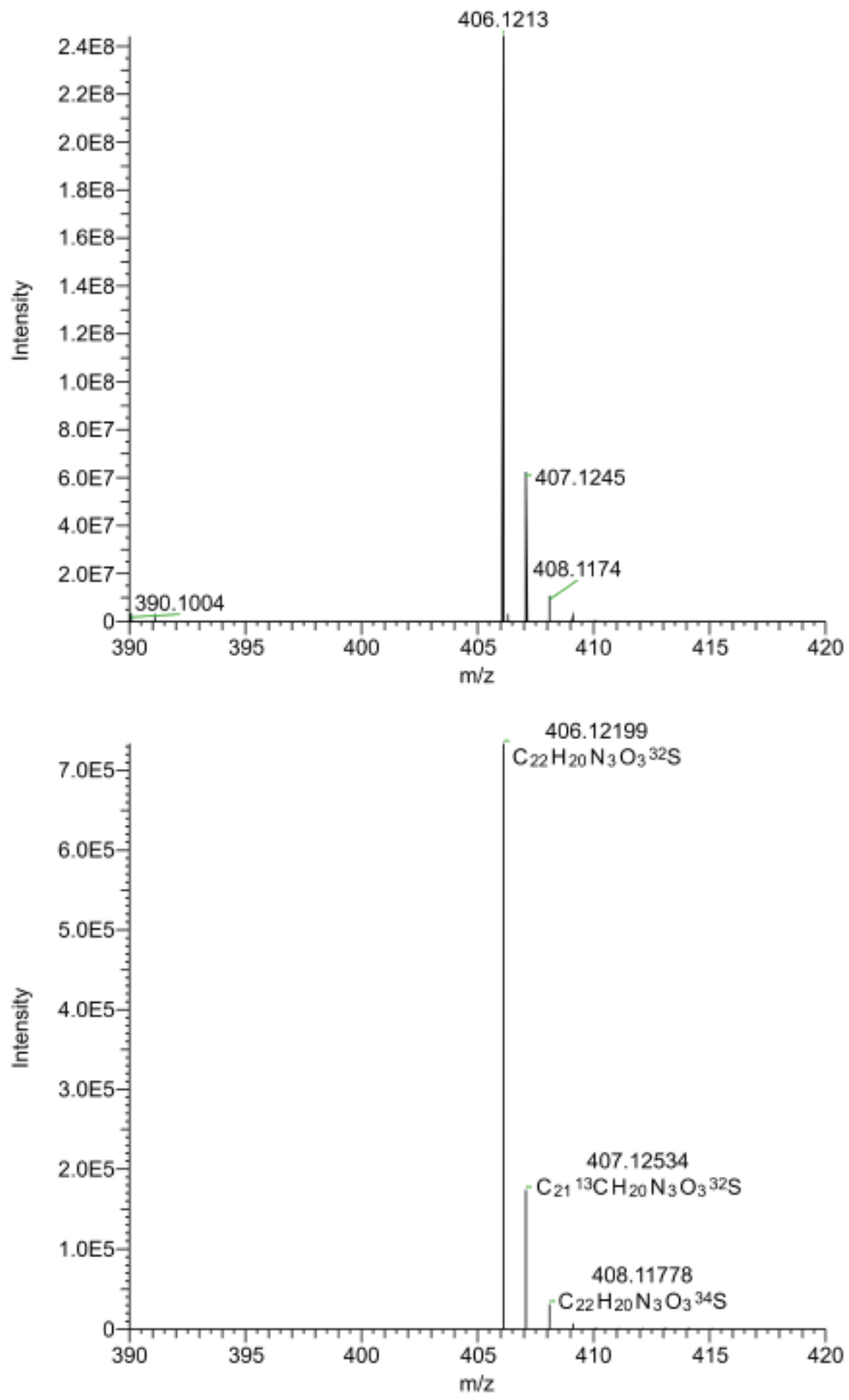

HRMS of compound **23**.

3. HPLC purity of target compounds

LC-UV analytical method for assessing the purity of key target compounds.

The purity of all tested compounds was determined by HPLC analysis and was greater than 95%. Stock solutions of test compounds were freshly prepared in HPLC-grade MeOH and further diluted. Analyses were performed on a:

1) Waters HPLC/UV/MS system (separation module Alliance HT2795, Photo Diode Array Detector 2996, mass detector Micromass ZQ; software: MassLynx 4.1). Separation was achieved using a Gemini® C6-Phenyl column (1500 mm × 4.6 mm i.d., 5-µm particle size). HPLC settings were as follows: flow rate, 1.0 mL/min; injection volume, 10.0 µL; detector wavelength, 254 nm. Three different methods were applied on this instrument:

Method A: linear gradient of 0.1% formic acid aqueous solution and acetonitrile 40/60 to 100% acetonitrile for 8 minutes then 100% acetonitrile for 2 minutes.

Method B: linear gradient of 0.1% formic acid aqueous solution and acetonitrile 30/70 to 100% acetonitrile for 8 minutes then 100% acetonitrile for 2 minutes.

Method C: linear gradient of 0.1% formic acid aqueous solution and acetonitrile 90/10 to 40/60 acetonitrile for 8 minutes then 100% acetonitrile for 2 minutes.

2) Agilent 1260 Infinity II system (Agilent Technologies, Santa Clara, CA, USA) coupled to an Agilent LC/MSD XT single-quadrupole mass spectrometer (Agilent Technologies, Santa Clara, CA, USA) equipped with the Agilent JetStream (AJS) electrospray ionization source and a UV–Vis diode-array detector (DAD, model G7115A). Separation was achieved using a XSelect^{TM} Premier CSH C18 column (100 * 4.6 mm, 3.5 µm particle size; Waters corporation, USA). HPLC settings were as follows: flow rate, 0.7 mL/min; injection volume, 5.0 µL; detector wavelength, 254 nm and 320 nm. Mobile phases consisted of water (eluent A) and acetonitrile (eluent B), both added with formic acid at 0.1% v/v.

Method D: T(0 min)= 95% A, 5% B. T(2 min) = 95% A, 5% B. T(12 min) = 5% A, 95% B. T(14 min) = 5% A, 95% B. T(16 min) 95% A, 5% B.

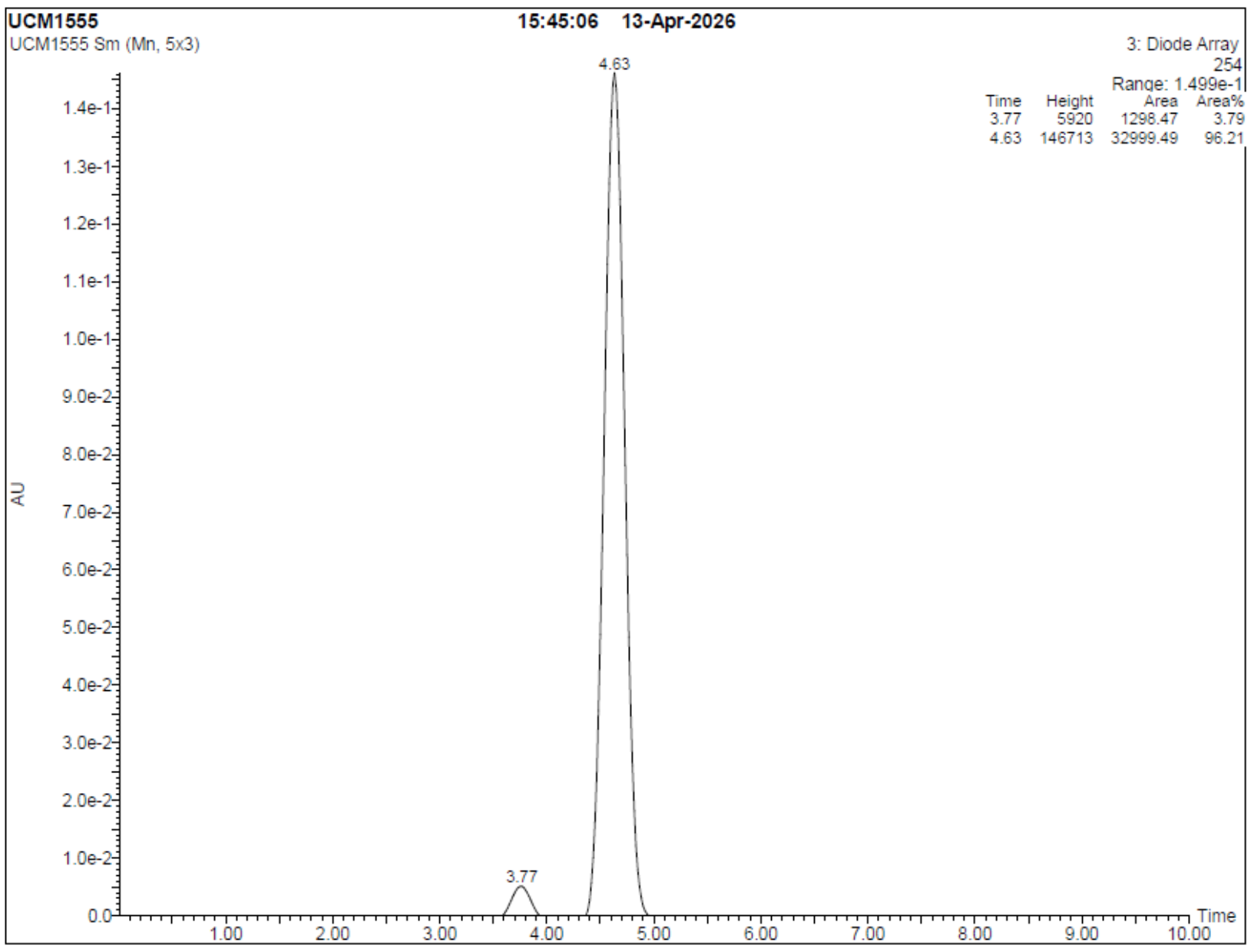

HPLC trace of compound **1a** (instrument 1, method A).

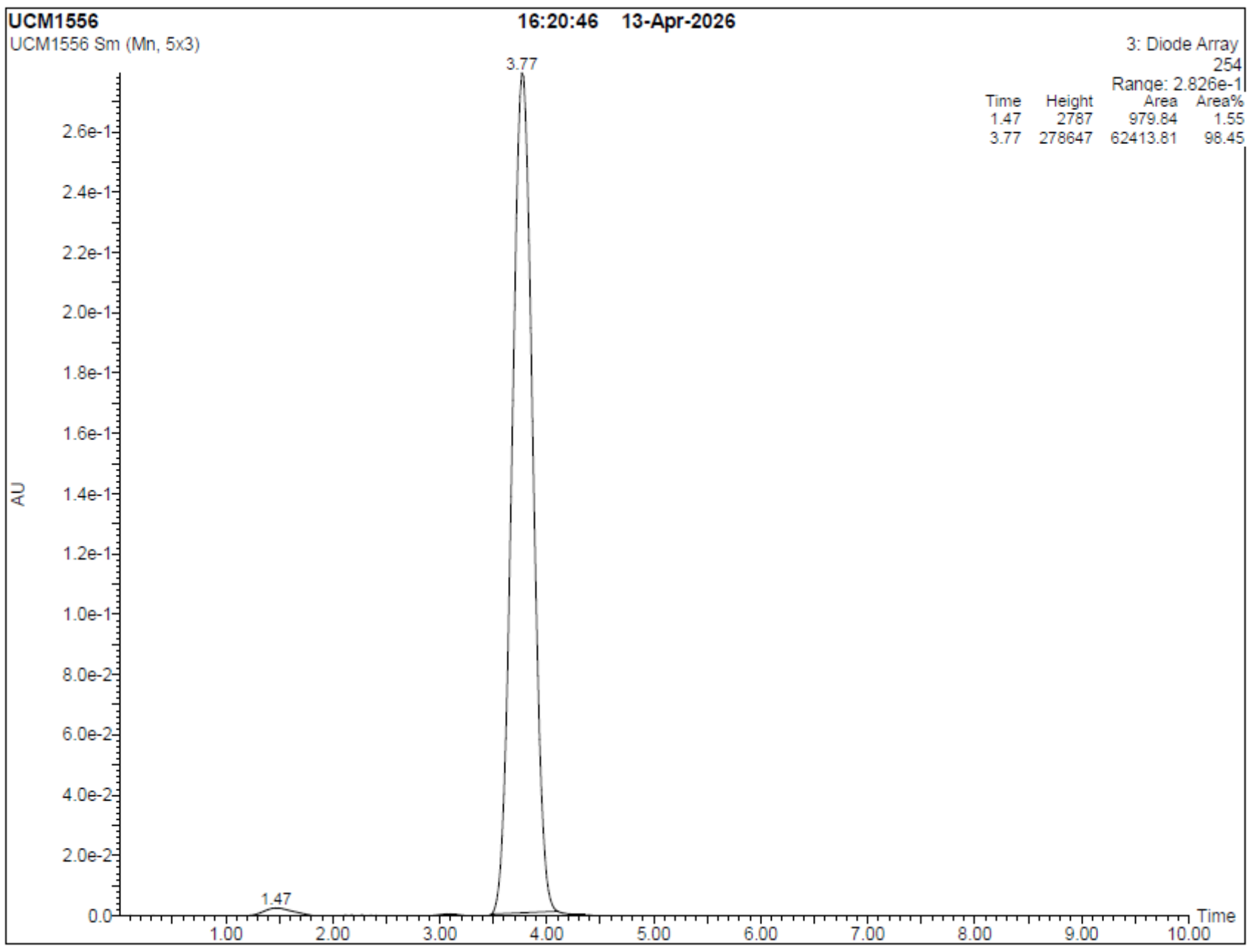

HPLC trace of compound **2a** (instrument 1, method A).

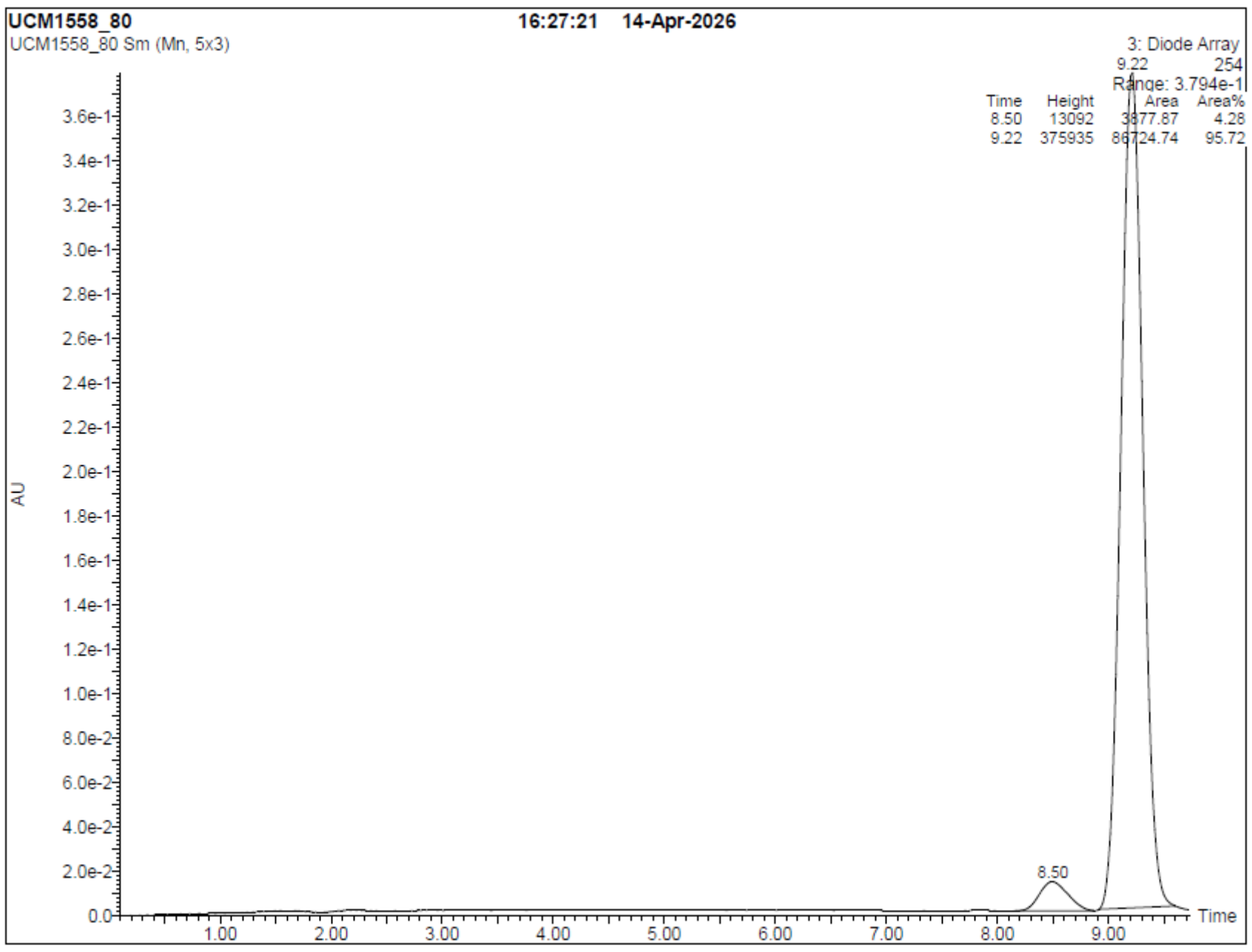

HPLC trace of compound **2b** (instrument 1, method B).

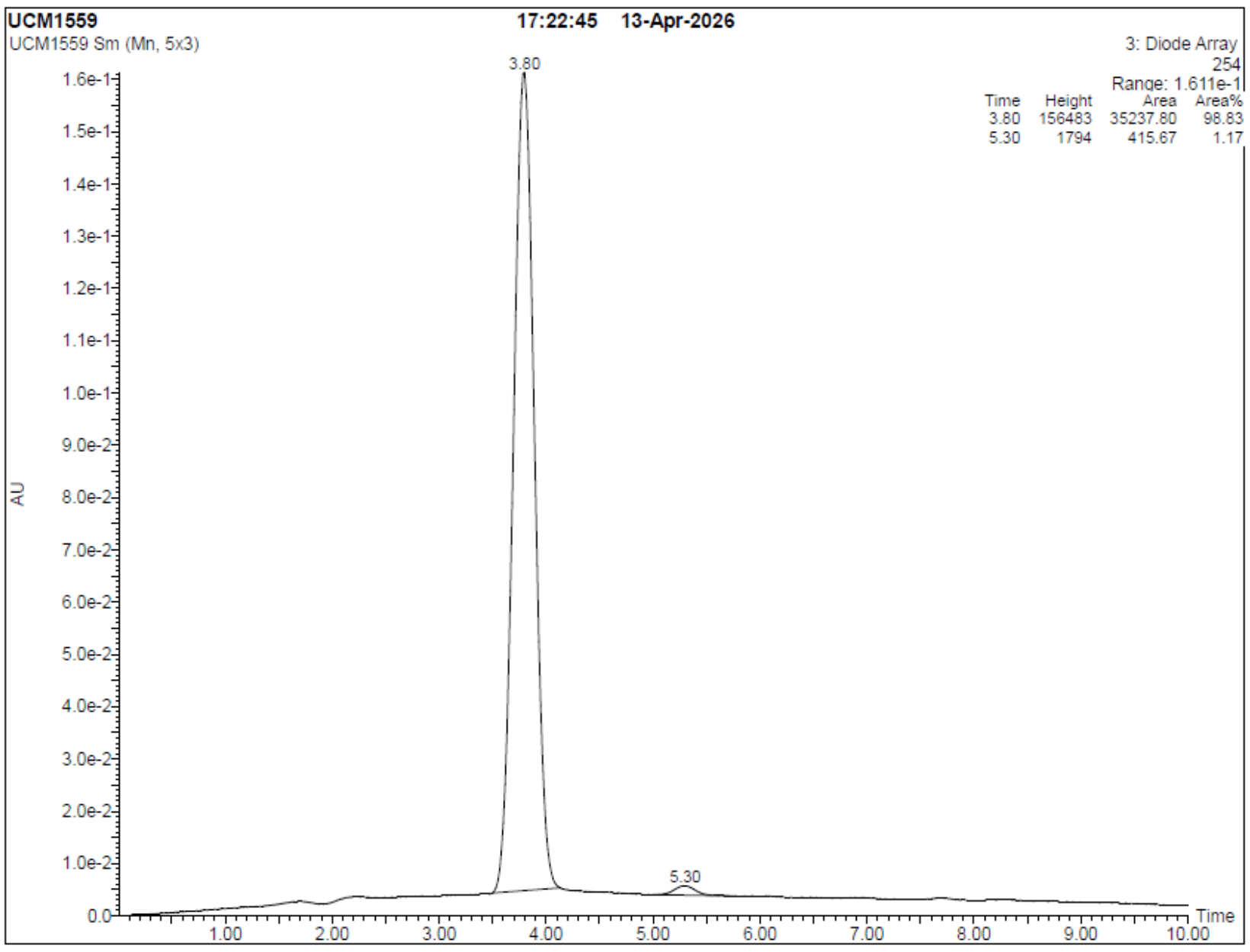

HPLC trace of compound **2c** (instrument 1, method A).

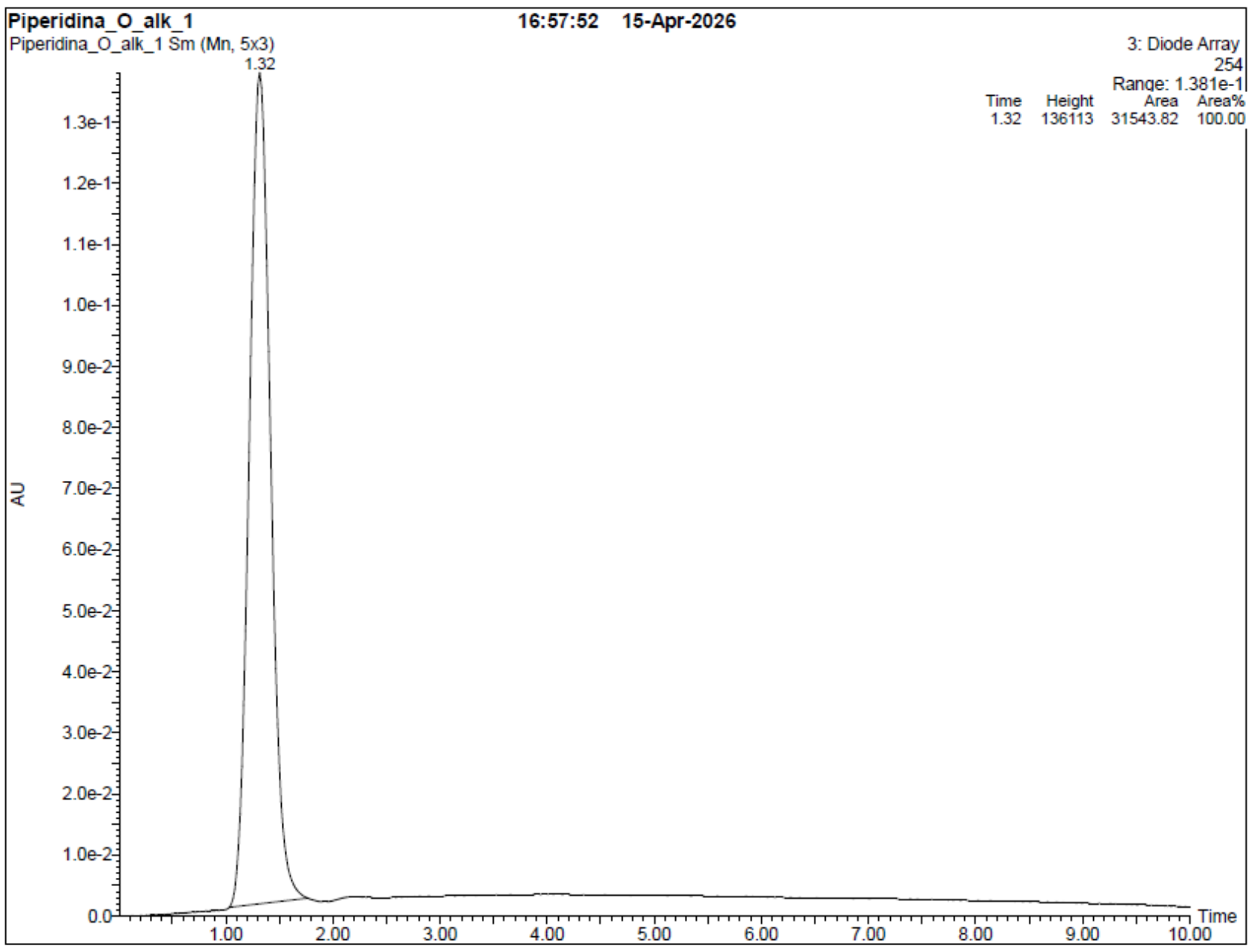

HPLC trace of compound **6** (instrument 1, method A).

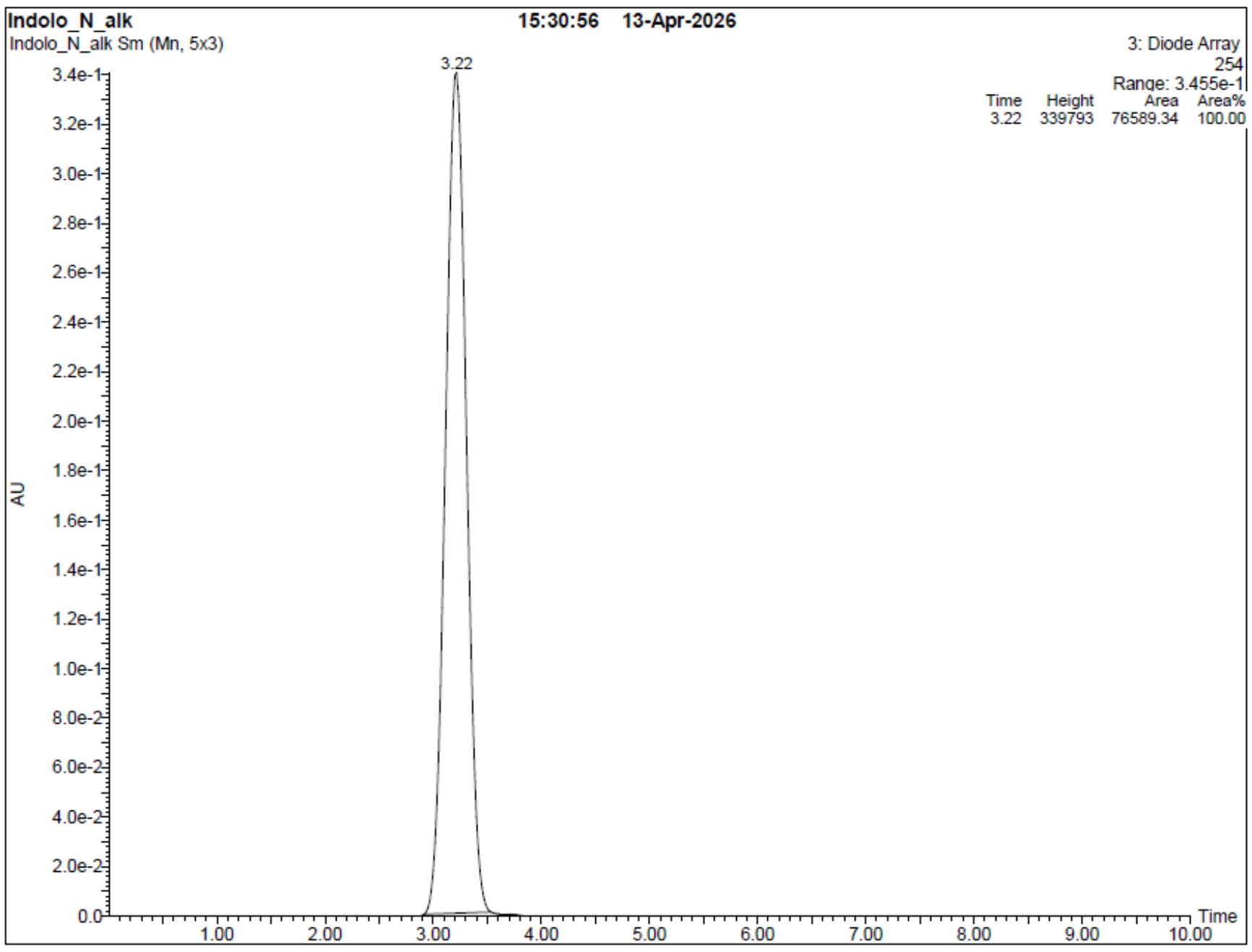

HPLC trace of compound **10** (instrument 1, method A).

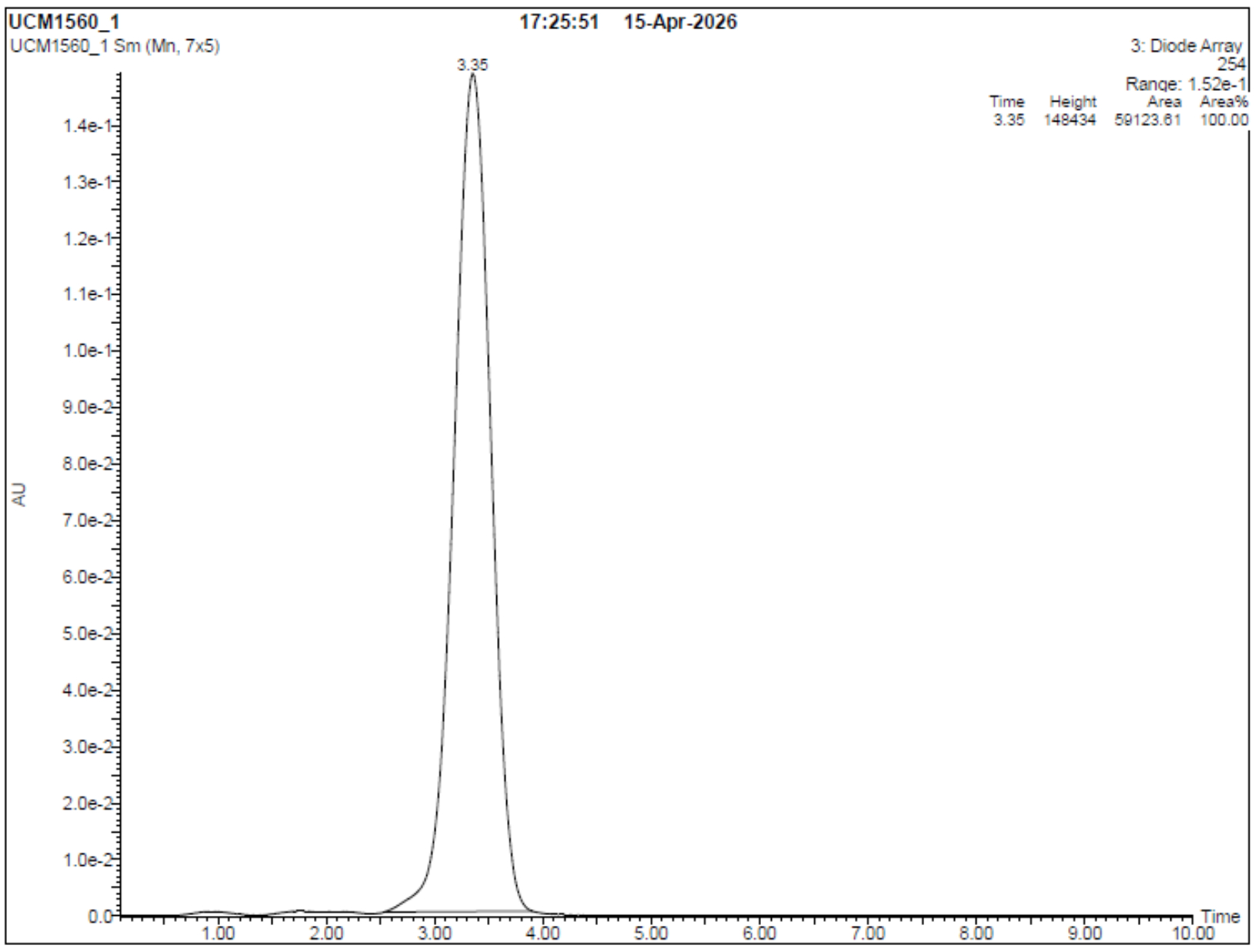

HPLC trace of compound **14** (instrument 1, method A).

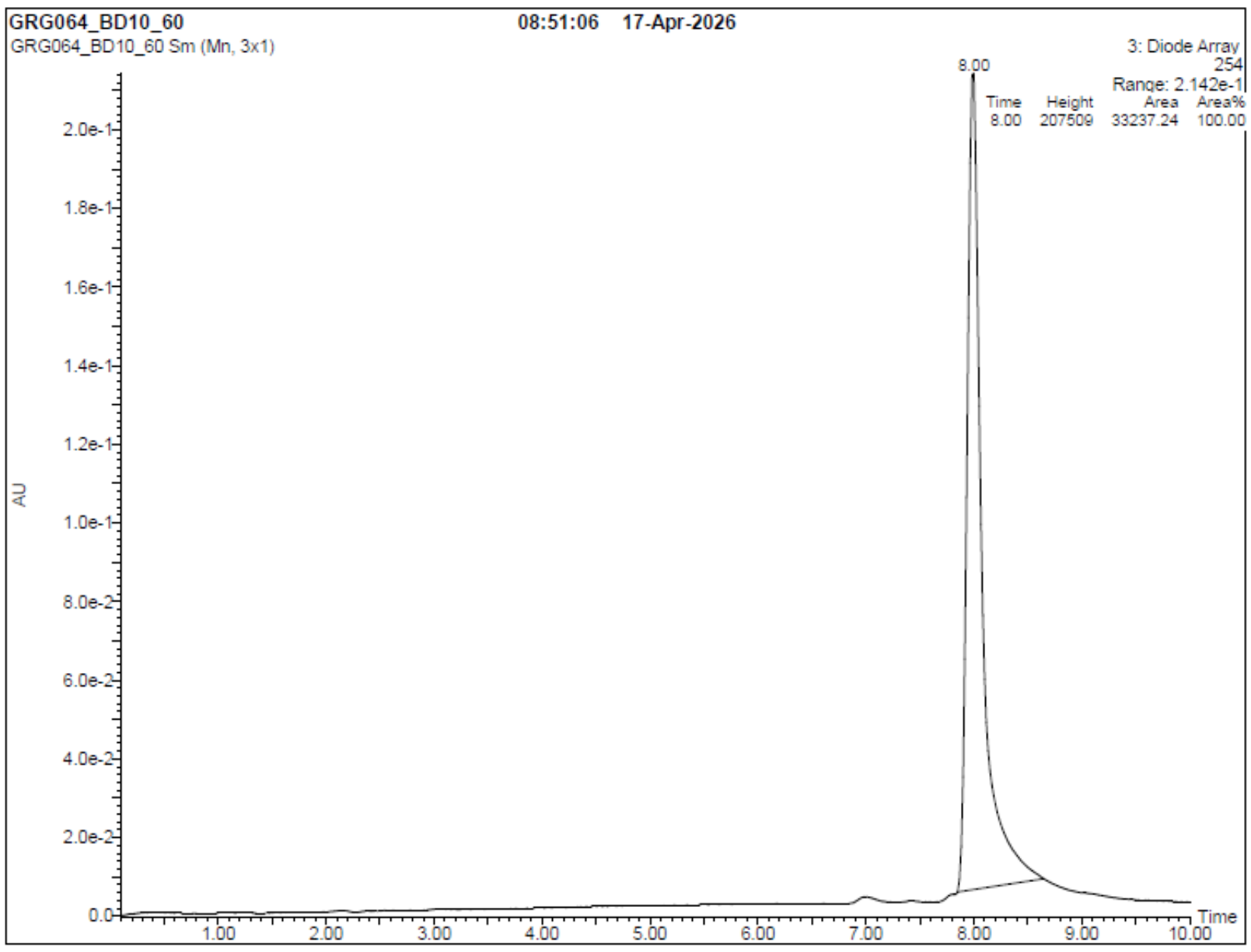

HPLC trace of compound **15** (instrument 1, method C).

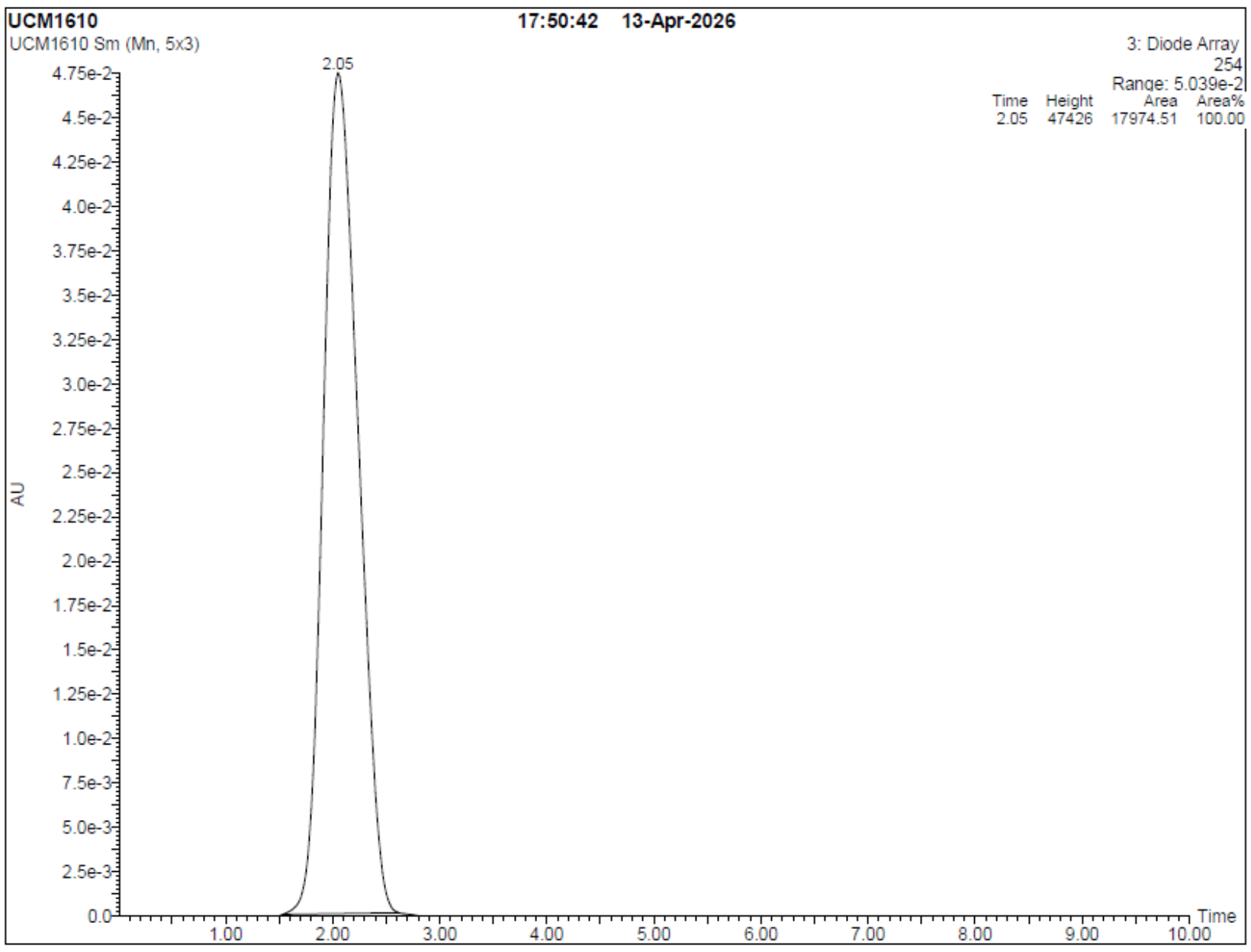

HPLC trace of compound **16** (instrument 1, method A).

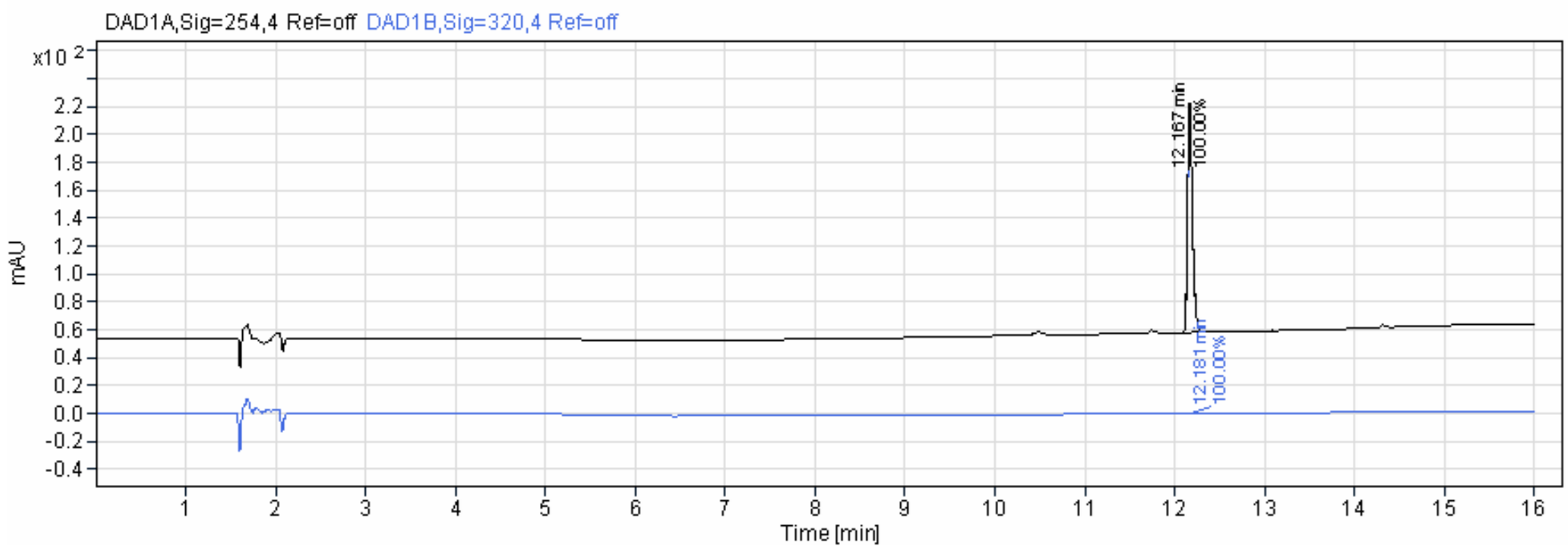

HPLC trace of compound **17** (instrument 2, method D).

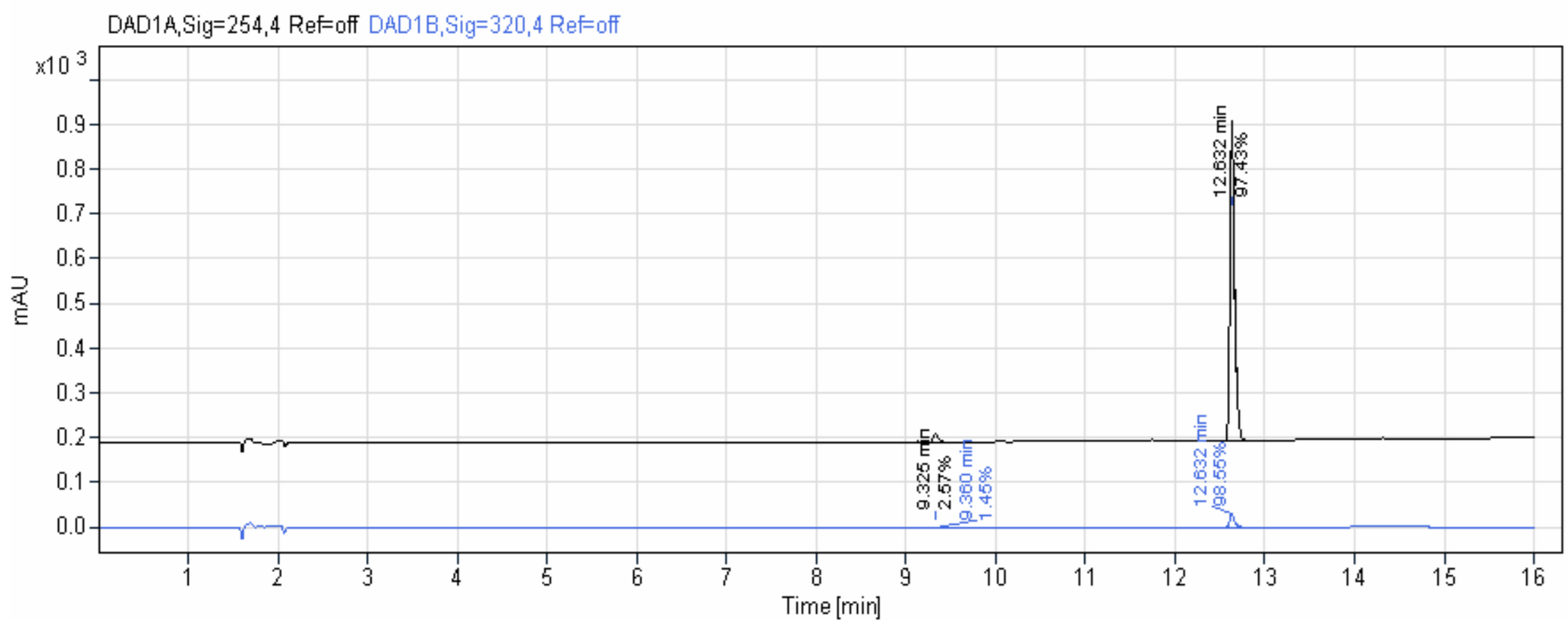

HPLC trace of compound **18** (instrument 2, method D).

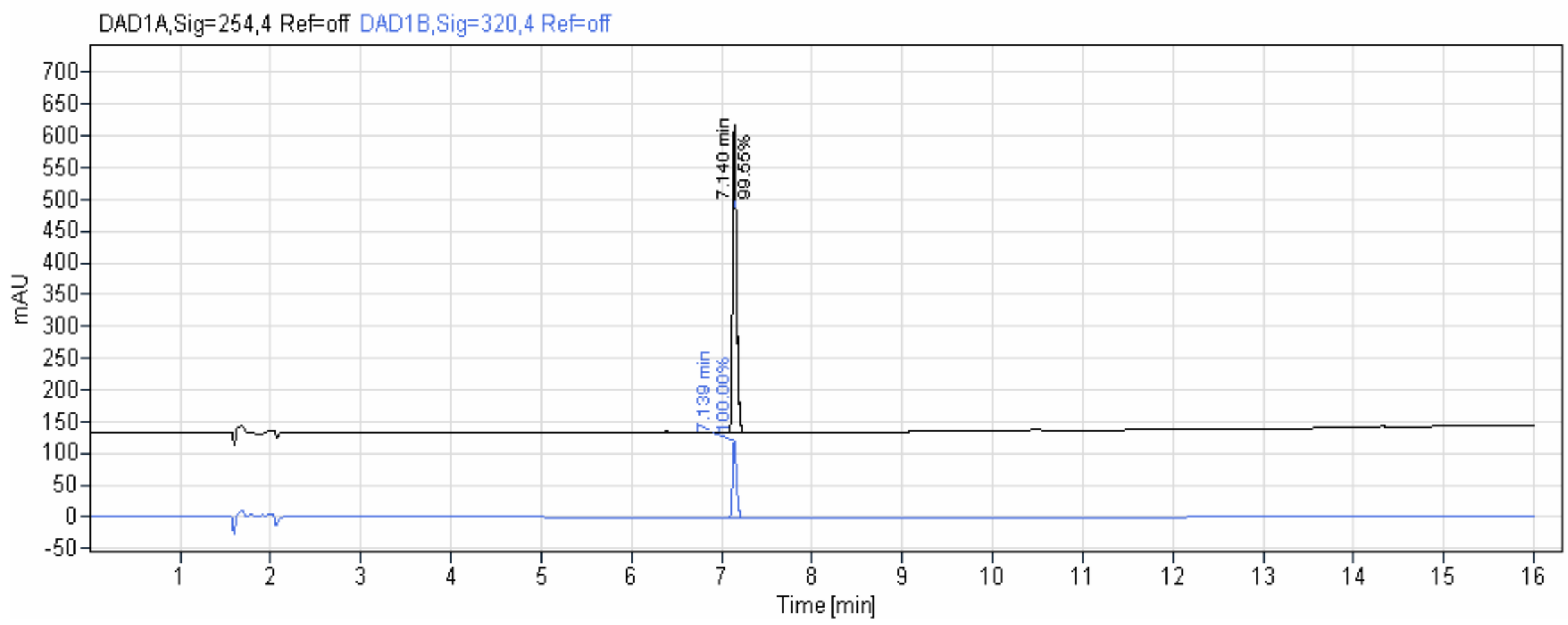

HPLC trace of compound **19** (instrument 2, method D).

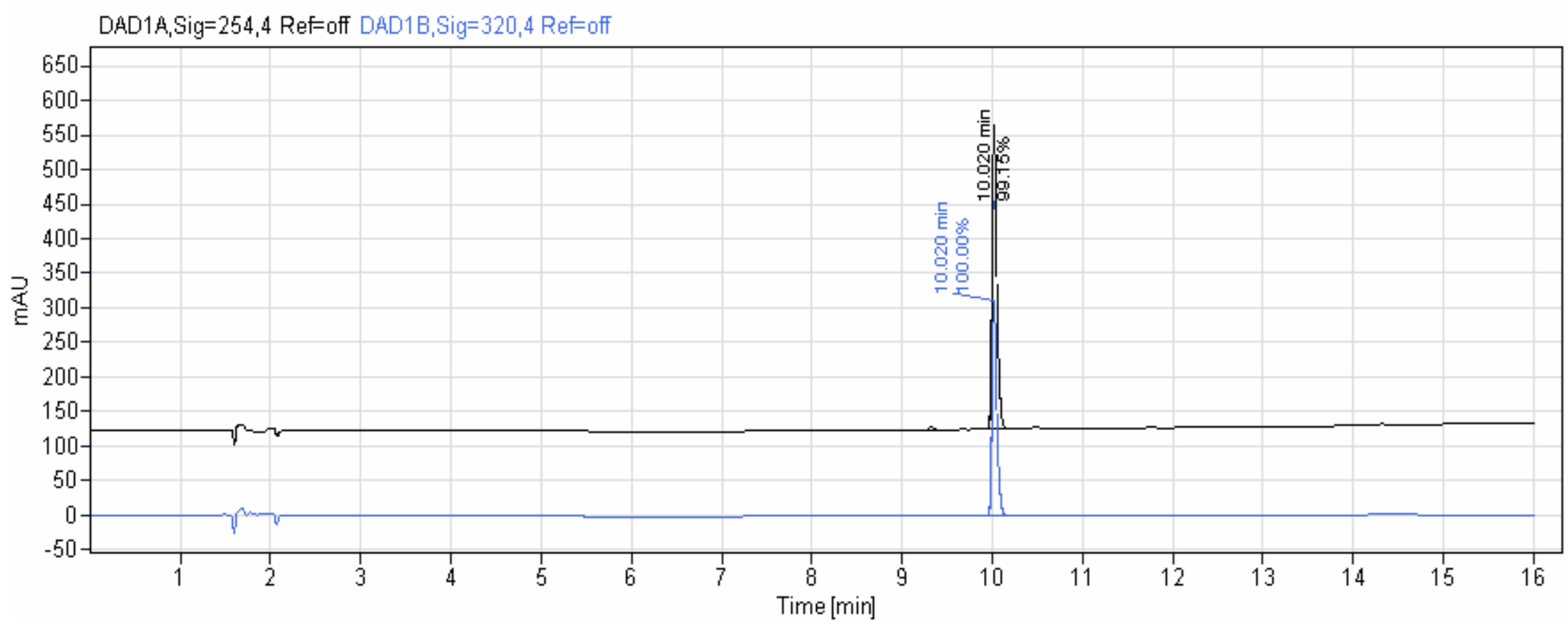

HPLC trace of compound **20** (instrument 2, method D).

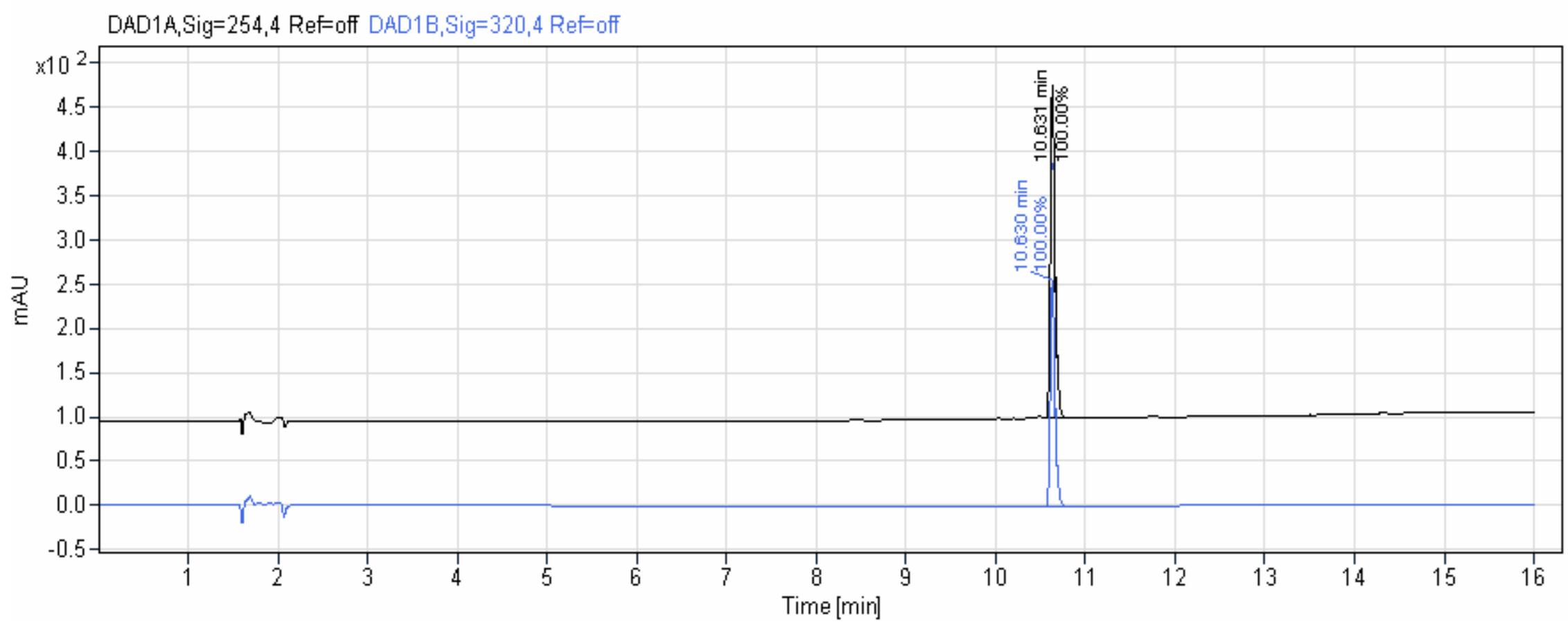

HPLC trace of compound **21** (instrument 2, method D).

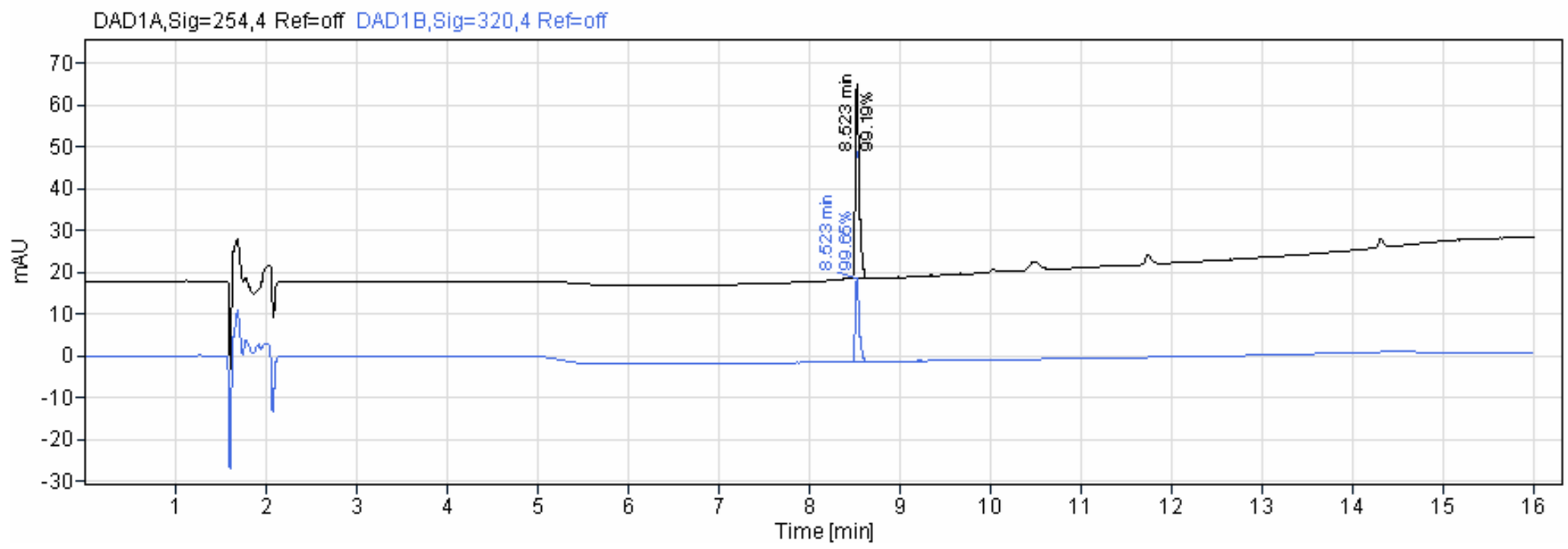

HPLC trace of compound **22** (instrument 2, method D).

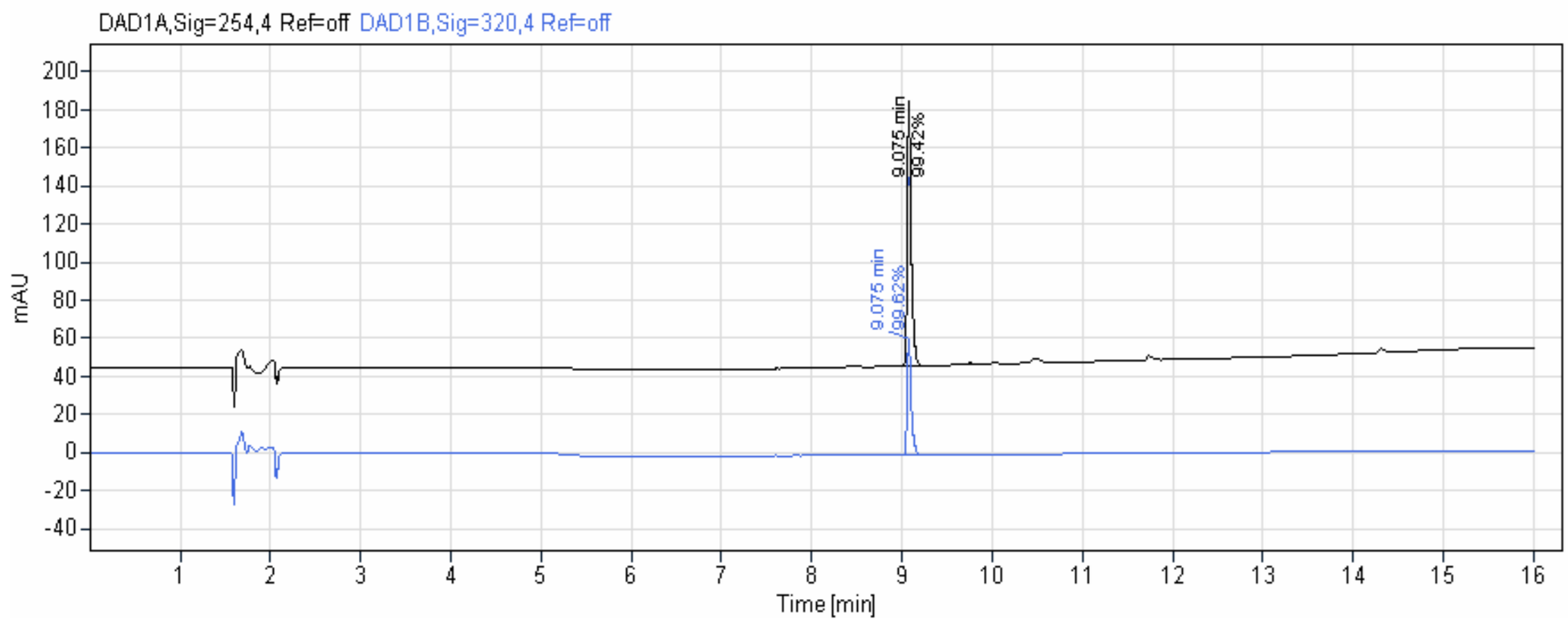

HPLC trace of compound **23** (instrument 2, method D).
